# Palladium-Catalyzed Oxidative Cyclization of *O*-Aryl Cyclic Vinylogous Esters: Synthesis of Benzofuran-Fused Cyclohexenones

**DOI:** 10.1021/acs.joc.4c02167

**Published:** 2024-11-29

**Authors:** Ko-Wang Yen, Chia-Chen Chein, Shih-Hsun Wung, Li-Ching Shen, Yen-Ku Wu

**Affiliations:** Department of Applied Chemistry, National Yang Ming Chiao Tung University, 1001 University Road, Hsinchu 30010, Taiwan

## Abstract

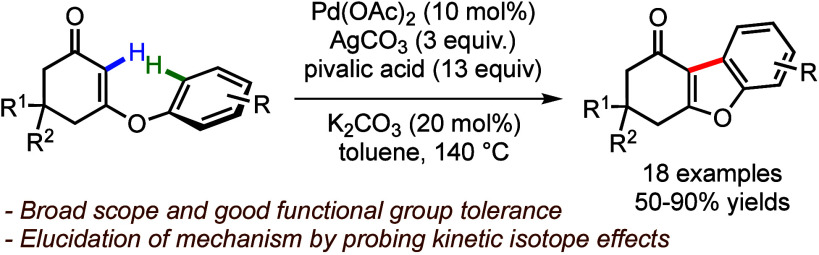

This study presents a method for synthesizing functionalized hydrodibenzofuran derivatives. Using palladium catalysis, *O*-aryl cyclic vinylogous esters undergo dehydrogenative intramolecular arylation at the vinylic carbon. Preliminary kinetic isotope effect studies suggest that the C(aryl)–H bond cleavage may be the rate-determining step.

The benzofuran skeleton is one of the fundamental heterocyclic motifs in natural products, small-molecule drugs, and organic materials.^1^ In recent decades, burgeoning progress has been made in developing transition-metal-catalyzed approaches to functionalized benzofurans and benzofuran-fused polycyclic compounds.^2^ In particular, the palladium(II)-mediated oxidative cyclization of carbonyl-functionalized precursors has appeared as a promising method to assemble biomedicinally relevant scaffolds. For instance, researchers have shown that benzofuran-fused quinones were prepared in modest yields through a dehydrogenative cyclization (Scheme 1a).^3^ More recently, McGlacken and co-workers converted aryloxy-substituted coumarins and pyrones to a series of benzofuran-fused frameworks (Scheme 1a).^4^ Wang and co-workers demonstrated oxidative cyclization reactions of 3-aryloxy-substituted acrylates and acrylonitriles (Scheme 1b).^5^ However, these processes mainly offered planar heteroaromatic frameworks. Inspired by the above-mentioned chemistry and an emerging interest in exploring C(sp^3^)-rich compounds for small-molecule drug discovery,^6^ we evaluated alternative approaches to saturated or partially saturated carbocycle-fused benzofurans.

**Scheme 1:**
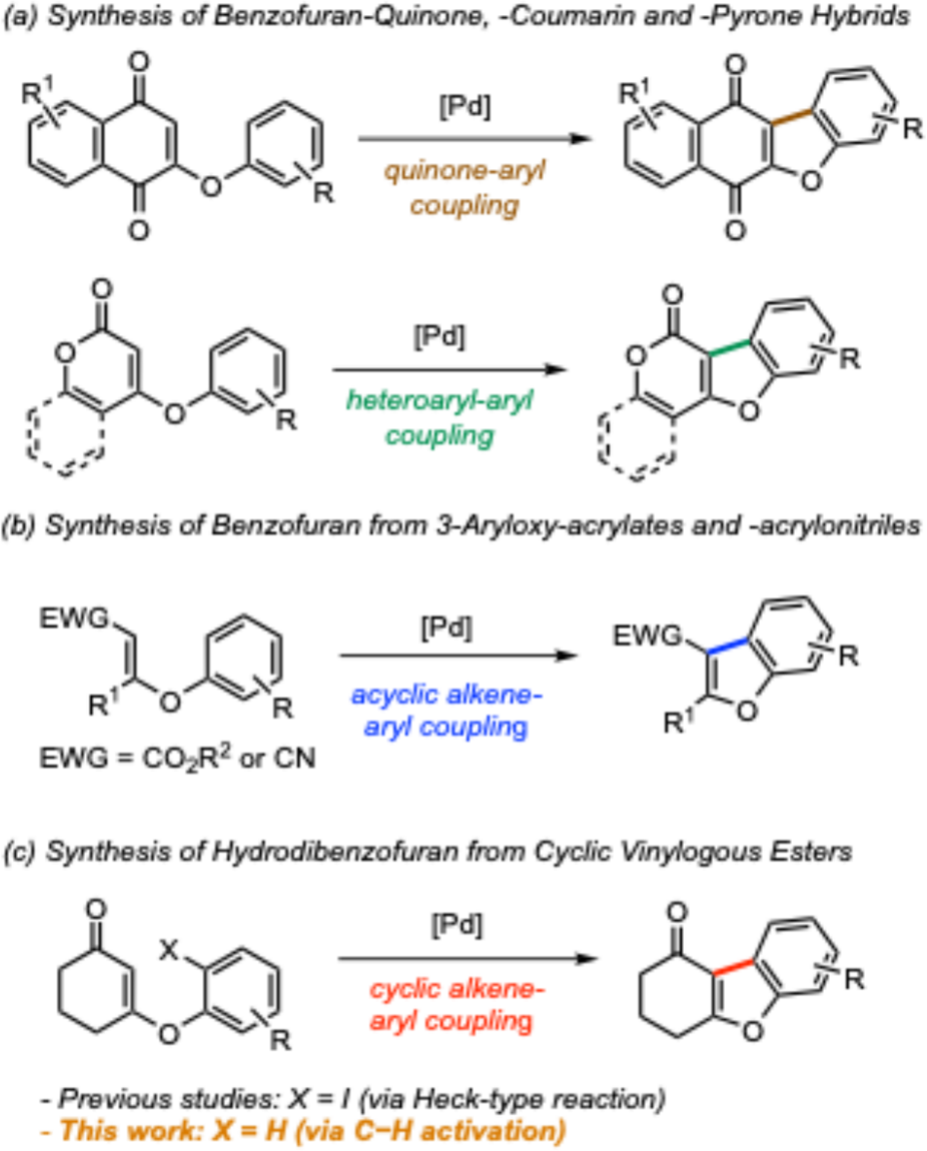
Comparison of Related Strategies for Benzofuran Formation

Cyclic vinylogous esters have long been exploited as versatile intermediates en route to various carbocyclic products.^7^ Recent developments in the catalytic monoarylation and polyarylation of cyclic vinylogous esters have offered a unique opportunity to rapidly assemble nonplanar (poly)aromatic structures.^8^ These deprotonative arylation reactions occur at the relatively acidic α- and/or γ′-carbons of cyclic vinylogous esters, and the regioselectivity of these processes could be precisely controlled under customized conditions. On the other hand, Ma, Banwell, and co-workers showed that when a 2-haloaryl group was tethered to the enol ether moiety of cyclic vinylogous esters, a palladium-catalyzed Heck reaction of these substrates delivered 3,4-dihydrodibenzo[*b*,*d*]furan-1(*2H*)-ones (Scheme 1c; X = I).^9^ However, this classic bond-forming event necessitates a labile halogen atom in the starting materials. To transcend the conventional reactivity paradigm, we sought to establish a dual C–H functionalization/cyclization of *O*-aryl cyclic vinylogous esters (Scheme 1c; X = H). This methodology would be empowered by double C–H functionalization, thus displaying notable strategic and economic merits in synthesis planning. However, several factors contribute to the difficulty of realizing such a process: (1) the lack of general access to the precursors and (2) the cyclic enone moiety is prone to aromatization under oxidative conditions. Despite the challenges, we present our research efforts in this domain.

Although acid-mediated condensation of cyclic 1,3-diketones with aliphatic alcohols is generally straightforward, the analogous reaction with less nucleophilic phenols was found to be low-yielding.^10^ In fact, there have been sporadic studies on the preparation of *O*-aryl cyclic vinylogous esters. Ma’s and Liu’s procedures appeared to be viable choices,^9a,11^ but only three cases and one case of making *O*-aryl cyclic vinylogous esters were disclosed in their reports, respectively. Ma’s transformation features the copper-catalyzed Ullmann–Goldberg cross-coupling reaction of 3-iodo-2-cyclohexen-1-one and phenols.^9a^ In our study, it is compatible with electron-rich and electron-neutral phenols (Method A, Scheme 2a); however, the efficiency was compromised with those phenols bearing a strong electron-withdrawing group. On the other hand, we found that Liu’s conditions allowed practical preparations of electron-deficient and electron-neutral congeners (Method B, Scheme 2b).^11^ These two protocols complement each other according to the electronic nature of the aryl donor. (Note: yield of **2g** by Method A is 48%, that by Method B is 12%; yield of **2i** by Method A is 15%, that by Method B is 52%). Expanding upon the prior art, we have established reliable access to a collection of *O*-aryl cyclic vinylogous esters.^12^

**Scheme 2:**
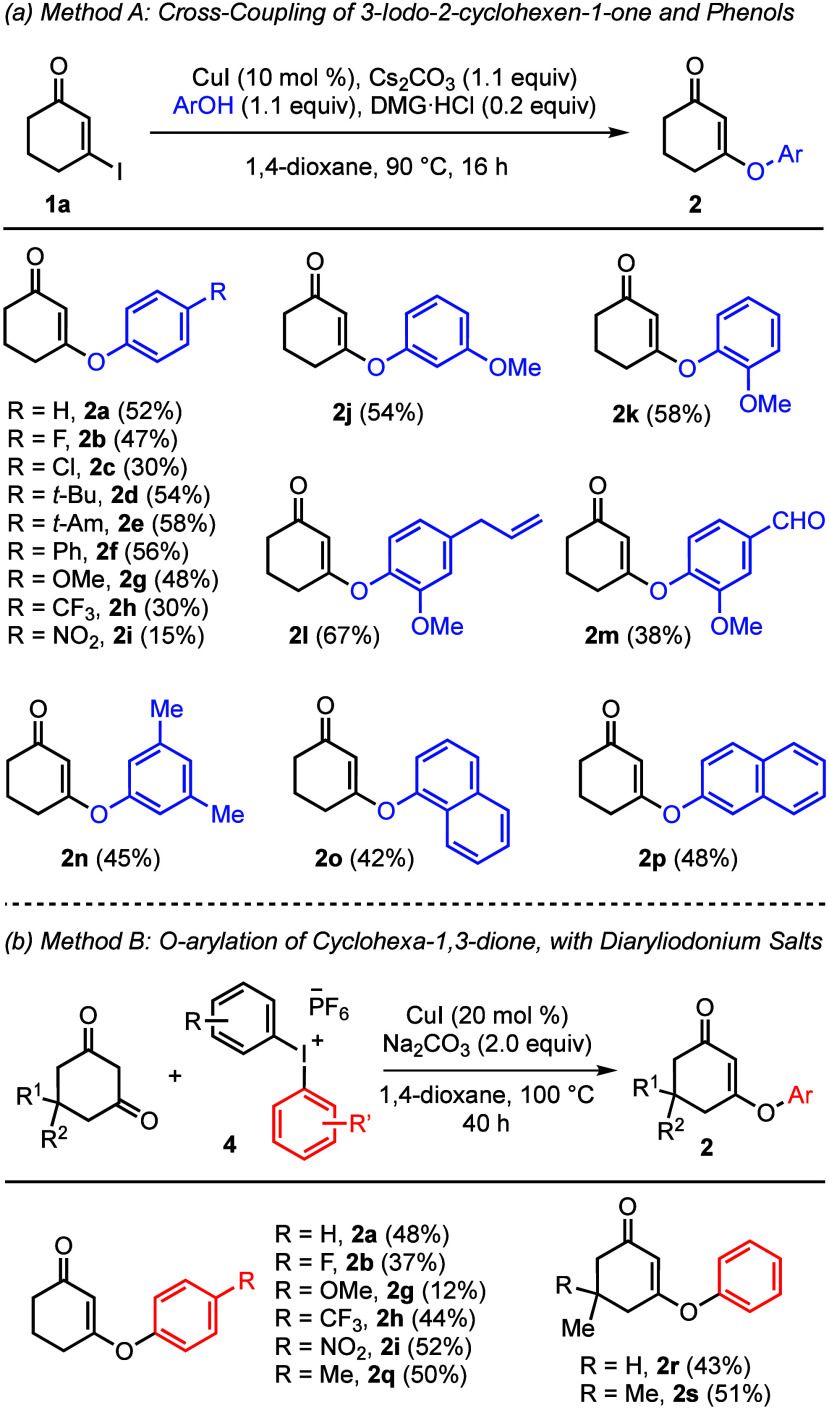
Syntheses of *O*-Aryl Cyclic Vinylogous Esters

Subsequently, we embarked on systematic studies of reaction parameters for the model oxidative cyclization (Table 1). At an early stage, Fagnou’s conditions for intramolecular oxidative biaryl coupling served as an inspiration for our optimization studies.^13^ The initial tests on a variety of oxidants (O_2_, AgOAc, Ag_2_O, Ag_2_CO_3_, and Cu(OAc)_2_) revealed that silver carbonate is the effective choice for this transformation.^14^ The reaction also proceeded with a substoichiometric amount of silver carbonate if copper(II) acetate was applied as a terminal oxidant (entry 5). We briefly examined the combination of silver carbonate with Cu(OAc)_2_ in different ratios, but no appreciable improvement was observed. In the absence of an oxidant, the control experiment delivered **3a** in less than 10% yield, indicating that the oxidant is critical for sustaining the dehydrogenative cyclization. Regarding the performance of selected palladium catalysts, palladium(II) acetate displayed better reactivity (cf. entries 2 and 7–9). Adding a suitable amount of potassium carbonate benefitted the model reaction (cf. entries 2 and 10–11). Increasing the reaction temperature and using 3 equiv of silver carbonate slightly improved the yields (entries 12–14). When the model reactions were conducted in polar aprotic solvents (DMSO, DMF, DMAc, and MeCN) and a protic solvent (1,1,1,3,3,3-hexafluoro-2-propanol), only a trace amount of **3a** was produced. Nonpolar aromatic solvents appeared to be superior reaction media, wherein toluene worked slightly better than *p*-xylene and benzene. Importantly, the cyclization failed to progress in the absence of pivalic acid, and the yield was higher when 13 equiv of this additive was applied (cf. entries 15–19). Preliminary attempts at reducing the loadings of Pd(OAc)_2_ unfortunately gave inferior reaction yields.^15^ The reaction on a larger scale is comparably efficient to the standard conditions (entry 18).

**Table 1:**
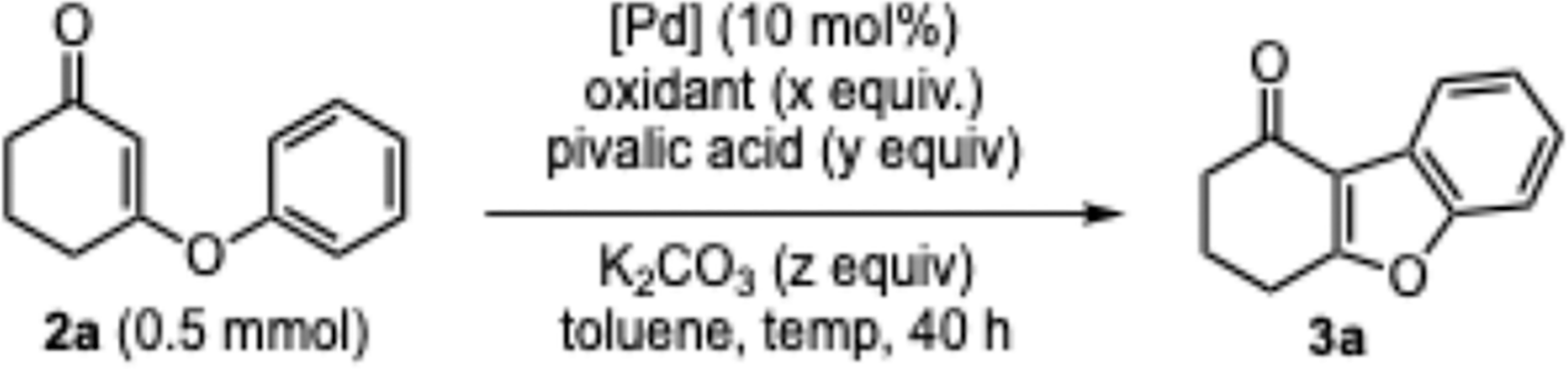
Screening of Conditions for the Dehydrogenative Cyclization

entry	[Pd]	oxidant (equiv)	temp (°C)	equiv of PivOH/K_2_CO_3_	yield (%)^a^
1	Pd(OAc)_2_	O_2_ (1 atm)	120	9/0.2	9
2	Pd(OAc)_2_	Ag_2_CO_3_ (1.5)	120	9/0.2	57
3	Pd(OAc)_2_	AgOAc (1.5)	120	9/0.2	38
4	Pd(OAc)_2_	Ag_2_O (1.5)	120	9/0.2	49
5	Pd(OAc)_2_	Cu(OAc)_2_ (1.4)	120	9/0.2	38
6	Pd(OAc)_2_	Ag_2_CO_3_ (0.1)/ Cu(OAc)_2_ (1.4)	120	9/0.2	48
7	Pd(TFA)_2_	Ag_2_CO_3_ (1.5)	120	9/0.2	51
8	Pd(OPiv)_2_	Ag_2_CO_3_ (1.5)	120	9/0.2	53
9	Pd(acac)_2_	Ag_2_CO_3_ (1.5)	120	9/0.2	47
10	Pd(OAc)_2_	Ag_2_CO_3_ (1.5)	120	9/–	46
11	Pd(OAc)_2_	Ag_2_CO_3_ (1.5)	120	9/1	38
12	Pd(OAc)_2_	Ag_2_CO_3_ (1.5)	130	9/0.2	58
13	Pd(OAc)_2_	Ag_2_CO_3_ (1.5)	140	9/0.2	64
14	Pd(OAc)_2_	Ag_2_CO_3_ (3)	140	9/0.2	65
15	Pd(OAc)_2_	Ag_2_CO_3_ (3)	140	0/0.2	trace
16	Pd(OAc)_2_	Ag_2_CO_3_ (3)	140	3/0.2	41
17	Pd(OAc)_2_	Ag_2_CO_3_ (3)	140	6/0.2	54
**18**	**Pd(OAc)**_**2**_	**Ag**_**2**_**CO**_**3**_**(3)**	**140**	13/0.2	74 (70)^b^
19	Pd(OAc)_2_	Ag_2_CO_3_ (3)	140	16/0.2	64

a Isolated yield. Please refer to the main text for comments on the additional survey of conditions.

b The reaction was conducted on 1 mmol scale.

With the optimized cyclization conditions in hand (Table 1, entry 18), the substrate scope was investigated (Scheme 3a). Substrates with various electron-donating and electron-withdrawing groups were successfully converted into the corresponding benzofuran products. Characteristic functionalities like nitro, formyl, methoxy, fluoro, chloro, and trifluoromethyl were compatible with these conditions. The reaction of an allyl-substituted substrate **2l** gave a complex mixture in which the cyclization product **3l** was not found. The presence of an *ortho*-substituent on the aryl moiety was well tolerated. The 1-naphthyl-substituted substrate **2o** possessing C2–H and C8–H bonds underwent highly *ortho*-selective C–H cyclization, and the *peri*-C–H functionalization did not occur to a noticeable extent.^16^ In the cases of *meta*-substituted precursor **2j** and 2-naphthanol-derived substrate **2p**, it is intriguing to observe exclusive formations of single regioisomers **3j** and **3p**, respectively. In the case of **3j**, the selectivity might be attributed to steric factors. On the other hand, the electronic factors of the 2-naphthyl core are responsible for the level of regioselectivity seen with **3p**.^17^ The oxidative cyclization of methyl- and *gem*-dimethyl-substituted cyclic vinylogous esters **2r** and **2s** proceeded smoothly to deliver the corresponding benzofuran products **3r** and **3s** in good yields. Intriguingly, the application of microwave irradiation in lieu of classical heating greatly improved the kinetic profile of the oxidative cyclization reaction (Scheme 3b); further investigation toward understanding the effects of microwaves^18^ on this transformation will be reported in due course.

**Scheme 3:**
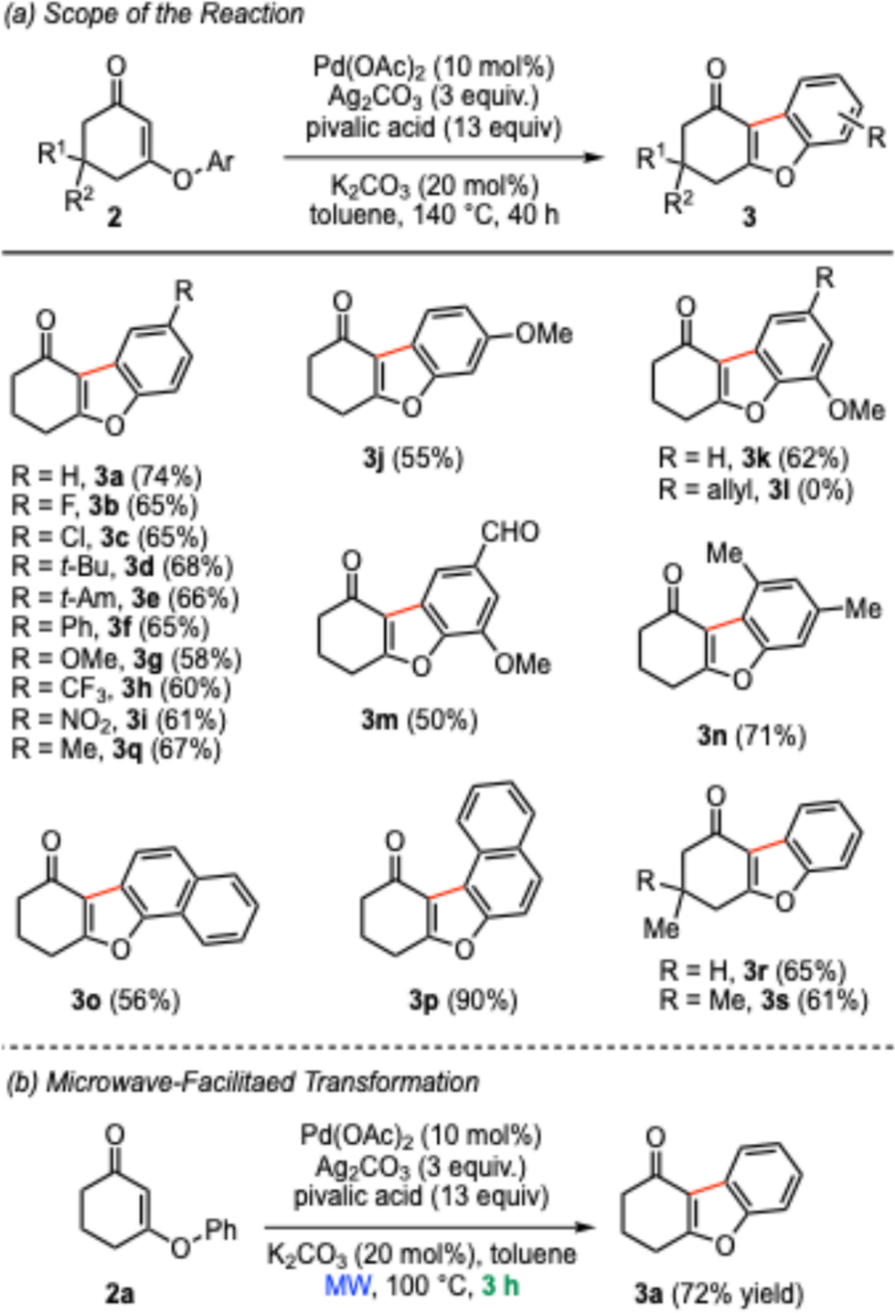
Synthesis of Benzofuran-Fused Cyclohexenones

We then shifted our focus to probe the reaction mechanism. Compound **2a** and its pentadeuterated analogue **2a-D**_**5**_ were employed for isotope effect studies (Scheme 4).^19^ By applying the initial rate protocol,^20^ the kinetic isotope effect (KIE) was determined to be 1.95; alternatively, the KIE obtained through an intermolecular competition experiment was 2.70 (see SI for further details, including H/D scrambling tests). These diagnostic results suggested that the cleavage of the C(aryl)–H bond was involved in the rate-determining step. Furthermore, because carboxylate/carbonate bases were essential to the process, a concerted metalation–deprotonation (CMD) mechanism^21^ was presumed to operate in the process. In accordance with the above-mentioned data, a plausible catalytic cycle featuring sequential electrophilic palladation at the vinyl position and CMD-type activation of the C–H bond was proposed and is shown in Scheme 5.

**Scheme 4:**
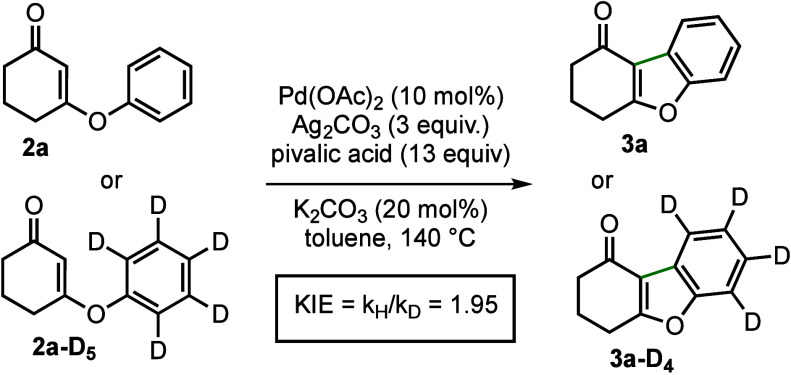
KIE Determined from Two Parallel Reactions

**Scheme 5:**
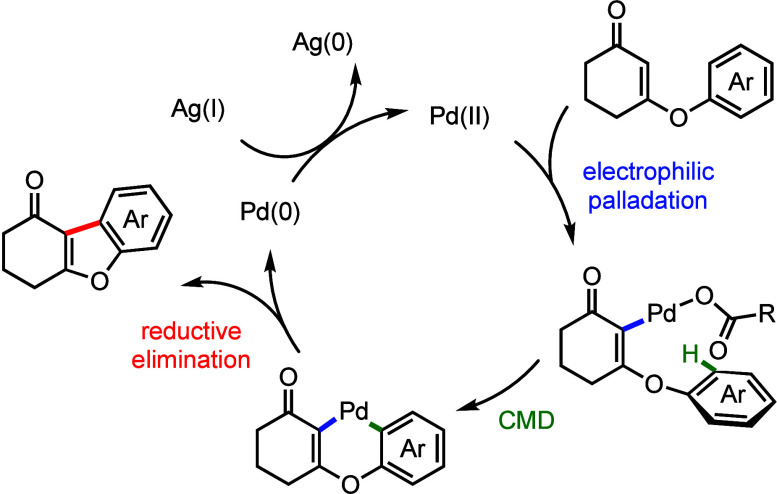
A Plausible Catalytic Cycle for the Oxidative Cyclization

In summary, we developed an intramolecular coupling of the proximal aryl and vinyl carbons in *O*-aryl cyclic vinylogous esters under palladium catalysis. The arylation sequence exploited the C–H functionalization strategy for the modular assembly of versatile benzofuran frameworks.

## Data Availability

The data underlying this study are available in the published article and its Supporting Information.

## Supporting Information Available

Experimental procedures, characterization data, and ^1^H and ^13^C NMR spectra (PDF)
