# Supplementary material for: Palladium-Catalyzed Oxidative Cyclization of O-Aryl Cyclic Vinylogous Esters: Synthesis of Benzofuran-Fused Cyclohexenones
Source: J Org Chem. 2024 Nov 29;89(24):18679–83. doi: 10.1021/acs.joc.4c02167 (PMC11667968; doi:10.1021/acs.joc.4c02167)

Palladium-Catalyzed Oxidative Cyclization of *O*-Aryl Cyclic Vinylogous Esters: Synthesis of Benzofuran-Fused Cyclohexenones

Ko-Wang Yen, Chia-Chen Chein, Shih-Hsun Wung, Li-Ching Shen and Yen-Ku Wu*

Department of Applied Chemistry, National Yang Ming Chiao Tung University, 1001 University Road, Hsinchu 30010, Taiwan

**E-mail : yenkuwu@nycu.edu.tw*

Supporting Information

Table of Contents

General Information S1

Synthesis and Characterization Data S2

Determination of Kinetic Isotope Effects S26

^{1}H and ^{13}C NMR Spectra S29

General Information

All reactions were conducted under N_2 if not specifically stated. Acetonitrile, 1,4-dioxane, toluene, and dichloromethane were purified by passage over activated alumina using a commercial solvent purification system. All other solvents (ACS grade) and commercially obtained reagents were used as received. Reactions were monitored by thin layer chromatography (TLC) on silica gel 60 Å F254 plates, visualized by UV (254 nm) and $KMnO_4$ (or phosphomolybdic acid) staining solution. Flash chromatography was performed on silica gel (230-400 mesh) with indicated eluents. Melting points were measured by Melting Point Apparatus (MP-2D) and were reported in Celsius (°C). Nuclear Magnetic resonance (NMR) spectra were recorded on Agilent 400-MR DD2 and JEOL JNM-ECZ400S/L1 in indicated deuterated solvents. Chemical shifts were recorded in ppm relative to residual non-deuterated solvent (δ 7.26 ppm for ^{1}H NMR and 77.16 for ^{13}C NMR in $CDCl_3$). Coupling constants were recorded in hertz (Hz) and multiplicities were abbreviated as follows: s (singlet), d (doublet), t (triplet), q (quartet), m (multiplet), br (broad). IR spectra were recorded on Thermo Nicolet iS5 FT-IR spectrometer with ATR sampling technique and were reported in wave number (cm^{-1}). High-resolution mass spectroscopy was performed on TOF instrument with EI and ESI. The microwave irradiation reaction was performed with a commercial microwave reactor (Discover Labmate, CEM Corporation), and the reaction vessel was also supplied by CEM cooperation.

Synthesis and Characterization Data

General Procedure A for the Synthesis of Diaryliodonium Hexafluorophosphate Salts

A round-bottom flask containing iodobenzene derivatives (4.5 mmol, 1 equiv.), 1,3,5-trimethyl-benzene (4.5 mmol, 1 equiv.), and *m*-chloroperoxybenzoic acid (*m*-CPBA; 4.95 mmol, 1.1 equiv.) in dichloromethane (40 mL, 0.1 M) was cooled down to 0 °C. Trifluoromethanesulfonic acid (TfOH; 13.5 mmol, 3 equiv.) was slowly added to the flask. The resulting mixture was stirred for 20 hours at room temperature. The solvent was removed under reduced pressure, and Et_2O was added. The mixture was placed in an ice/water bath. The resulting precipitate was collected by filtration. The crude salt was dissolved in dichloromethane and then extracted with an aqueous KPF_6 (22.5 mmol, 5 equiv.) solution five times. The combined organic layers were dried over anhydrous $MgSO_4$, filtered, and concentrated under reduced pressure to give the corresponding diaryliodonium hexafluorophosphates.

General Procedure B for the Synthesis of *O*-Aryl Cyclic Vinylogous Esters

A round-bottom flask containing 3-iodocyclohex-2-enone (1.0 mmol, 1 equiv.), phenol derivatives (1.1 mmol, 1.1 equiv.), copper(I) iodide (0.1 mmol, 0.1 equiv.), cesium carbonate (0.1 mmol, 0.1 equiv.), and DMG·HCl (*N*,*N*-dimethylglycine (DMG) hydrochloride; 0.2 mmol, 0.2 equiv.) in 1,4-dioxane (5 mL, 0.2 M) was stirred at 90 °C (in an oil bath) for 16 hours. The reaction mixture was cooled to room temperature, diluted with dichloromethane, and extracted with water. The combined organic layers were dried over anhydrous $MgSO_4$, filtered, and concentrated under reduced pressure. The crude thus obtained was purified by flash column chromatography to give the corresponding *O*-aryl cyclic vinylogous esters.

General Procedure C for the Synthesis of *O*-Aryl Cyclic Vinylogous Esters

A sealed tube containing 1,3-cyclohexanedione derivatives (3.0 mmol, 1.2 equiv.), diaryliodonium salts (2.5 mmol, 1 equiv.), copper(I) iodide (0.5 mmol, 0.2 equiv.), and sodium carbonate (5.0 mmol, 2 equiv.) in 1,4-dioxane (5 mL, 0.5 M) was stirred at 100 °C (in an oil bath) for 40 hours. The reaction mixture was cooled to room temperature, diluted with dichloromethane, and extracted with water. The combined organic layers were dried over anhydrous $MgSO_4$, filtered, and concentrated under reduced pressure. The crude thus obtained was purified by flash column chromatography to give the corresponding products.

General Procedure D for Palladium-Catalyzed Oxidative Cyclization of *O*-Aryl Cyclic Vinylogous Esters

A sealed tube containing *O*-aryl cyclic vinylogous esters (0.5 mmol, 1 equiv.), silver carbonate (1.5 mmol, 3 equiv.), potassium carbonate (0.1 mmol, 0.2 equiv.), pivalic acid (6.5 mmol, 13 equiv.) and palladium acetate (0.05 mmol, 0.1 equiv.) in toluene (5 mL, 0.1 M) was stirred at 140 °C (in an oil bath) for 40 hours. After the limiting reagent was consumed, as indicated by TLC analysis, the reaction was cooled to room temperature and quenched with a saturated aqueous sodium bicarbonate solution. The mixture was diluted with dichloromethane and extracted with water. The combined organic layers were dried over anhydrous $MgSO_4$, filtered, and concentrated under reduced pressure. The crude thus obtained was purified by flash column chromatography to give the corresponding products.

3-Iodocyclohex-2-en-1-one (1a)[1]

[1] Khan, F.; Dlugosch, M.; Liu, X.; Khan, M.; Banwell, M. G.; Ward, J. S.; Carr, P. D. *Org. Lett.* **2018**, *20*, 2770-2773.

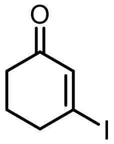

To a round-bottom flask containing triphenylphosphine (11 mmol, 2.89 g) and iodine (11 mmol, 2.79 g) was added acetonitrile (50 mL). After stirring for 30 minutes at room temperature, triethylamine (1.53 mL, 11 mmol) and 1,3-cyclohexanedione (10 mmol, 1.12 g) were added, and the mixture was refluxed (in an oil bath) for 16 hours. After the limiting reagent was consumed, as confirmed by TLC analysis, the reaction mixture was cooled to room temperature, and the solvent was removed under reduced pressure. Et_2O (30 mL) was added, and then the solid was filtered out; this step was repeated. The combined filtrates were diluted with petroleum ether (50 mL) and then filtered with a short pad of celite/silica gel mixture. The filtrate was concentrated under reduced pressure to afford **1a** (1.7 g, 77 %) as pale yellow oil. R_f: 0.45 (hexanes/EtOAc = 4/1). **^{1}H NMR** (400 MHz, $CDCl_3$): δ 6.80 (s, 1H), 2.90 (t, *J* = 6.0 Hz, 2H), 2.42 (t, *J* = 6.8 Hz, 2H), 2.02 (m, 2H).

4-Fluorophenyl(mesityl)iodonium hexafluorophosphate (1b)[2]

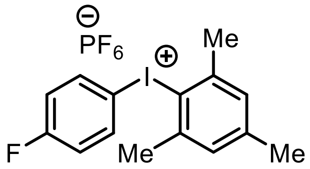

Following General Procedure A, the reaction was conducted with 1-fluoro-4-iodobenzene (4.49 mmol, 1.00 g) to give **1b** (875 mg, 40%) as brown solid. m.p. 177-178 °C; **^{1}H NMR** (400 MHz, CD_3OD) δ 7.97 (m, 2H), 7.27 (t, *J* = 8.7 Hz, 2H), 7.23 (s, 2H), 2.67 (s, 6H), 2.34 (s, 3H).

Bis(4-methoxyphenyl)iodonium hexafluorophosphate (1c)[3]

---

[2] Harvey, J. S.; Simonovich, S. P.; Jamison, C. R.; MacMillan, D. W. *J. Am. Chem. Soc.* **2011**, *133*, 13782–13785.

[3] Xu, Z.-J.; Liu, X.-Y.; Zhu, M.-Z.; Xu, Y.-L.; Yu, Y.; Xu, H.-R.; Cheng, A.-X.; Lou, H.-X. *Org. Lett.*

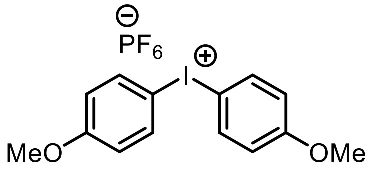

To a round-bottom flask containing methoxybenzene (5.8 mmol, 625 mg), iodine (I_2; 2.9 mmol, 736 mg), and *m*-chloroperoxybenzoic acid (*m*-CPBA; 11.6 mmol, 2.00 g) in dichloromethane (80 mL) was added TsOH·H_2O (11.6 mmol, 2.20 g), and the mixture was stirred at room temperature for 20 hours. TfOH (5.8 mmol, 870 mg) was slowly added to the solution. After stirring for an hour at room temperature, the solvent was removed under reduced pressure, and Et_2O was added. The mixture was placed in an ice/water bath. The resulting precipitate was collected by filtration. The crude salt was dissolved in dichloromethane and then extracted with an aqueous KPF_6 (29 mmol, 5.33 g) solution five times. The combined organic layers were dried over anhydrous $MgSO_4$, filtered, and concentrated under reduced pressure to give **1c** (1998 mg, 71%) as brown solid. m.p. 198-199 °C; **^{1}H NMR** (400 MHz, CD_3OD) δ 8.04 (d, J = 9.2 Hz, 4H), 7.03 (d, J = 9.1 Hz, 4H), 3.83 (s, 6H).

Mesityl(4-(trifluoromethyl)phenyl)iodonium hexafluorophosphate (1d)[4]

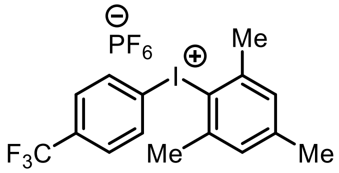

Following General Procedure A, the reaction was conducted with 1-iodo-4-(trifluoromethyl)benzene (4.5 mmol, 1224 mg) to give **1d** (700 mg, 29%) as yellow solid. m.p. 174-175 °C; **^{1}H NMR** (400 MHz, CD_3OD) δ 8.08 (d, J = 8.4 Hz, 2H), 7.81 (d, J = 8.4 Hz, 2H), 7.27 (s, 2H), 2.66 (s, 6H), 2.37 (s, 3H).

2021, *23*, 9073–9077.

[4] Lukamto, D. H.; Gaunt, M. J. *J. Am. Chem. Soc.* **2017**, *139*, 9160–9163.

Mesityl(4-nitrophenyl)iodonium hexafluorophosphate (1e)

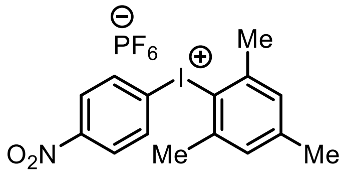

Following General Procedure A, the reaction was conducted with 1-iodo-4-nitrobenzene (4.7 mmol, 1171 mg) to give **1e** (700 mg, 29%) as yellow solid. m.p. 168-169 °C; **IR** (cast):1602, 1570, 1531, 1470, 1352, 1302, 1001, 838, 735 cm^{-1}; **^{1}H NMR** (400 MHz, CD_3OD) δ 8.31 (d, *J* = 9.1 Hz, 2H), 8.11 (d, *J* = 9.1 Hz, 2H), 7.29 (s, 2H), 2.67 (s, 6H), 2.38 (s, 3H); **^{13}C{^{1}H} NMR** (100 MHz, CD_3OD) δ 151.4, 146.5, 143.7, 136.2, 131.6, 127.6, 122.4, 119.9, 27.1, 21.1; **HRMS** (ESI, [M^+]) for $C_{15}H_{15}INO_2$ calcd. 368.0142, found: 368.0139.

Mesityl(*p*-tolyl)iodonium hexafluorophosphate (1f)[4]

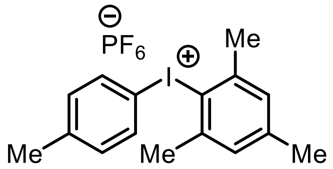

Following General Procedure A, the reaction was conducted with 1-iodo-4-methylbenzene (4.5 mmol, 980 mg) to give **1f** (1150 mg, 53%) as white solid. m.p. 199-200 °C; **^{1}H NMR** (400 MHz, CD_3OD) δ 7.78 (d, *J* = 8.4 Hz, 2H), 7.33 (d, *J* = 8.5 Hz, 2H), 7.23 (s, 2H), 2.66 (s, 6H), 2.39 (s, 3H), 2.35 (s, 3H).

3-Phenoxycyclohex-2-en-1-one (2a)[5]

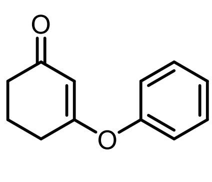

Following General Procedure B, the reaction was conducted with **1a** (1.0 mmol, 222 mg)

---

[5] Trost, B. M.; Bream, R. N.; Xu, J. *Angew. Chem. Int. Ed.* **2006**, *118*, 3181–3184.

and phenol (1.1 mmol, 104 mg). The crude product was purified by flash column chromatography (hexanes/EtOAc = 8/1 to 6/1) to afford **2a** (98 mg, 48%) as yellow oil [Note: The reaction by General Procedure C gave **2a** in 15% yield]. R_f: 0.33 (hexanes/EtOAc = 4/1); **^{1}H NMR** (400 MHz, $CDCl_3$) δ 7.37 (dd, *J* = 7.9,7.4 Hz, 2H), 7.22 (t, *J* = 7.4 Hz, 1H), 7.01 (d, *J* = 7.9 Hz, 2H), 5.10 (s, 1H), 2.64 (t, *J* = 6.4 Hz, 2H), 2.35 (t, *J* = 6.4 Hz, 2H), 2.06 (tt, *J* = 6.4, 6.4 Hz, 2H).

3-(4-Fluorophenoxy)cyclohex-2-en-1-one (2b)

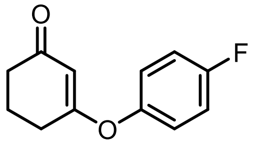

Following General Procedure B, the reaction was conducted with **1a** (1.0 mmol, 222 mg) and 4-fluorophenol (1.1 mmol, 123 mg). The crude product was purified by flash column chromatography (hexanes/EtOAc = 8/1 to 6/1) to afford **2b** (97 mg, 47%) as yellow oil [Note: The reaction by General Procedure C gave **2b** in 37% yield]. R_f: 0.32 (hexanes/EtOAc = 4/1); **IR** (film): 2956, 1575, 1507, 1219, 1183, 1139, 830, 772, 747 cm^{-1}; **^{1}H NMR** (400 MHz, $CDCl_3$) δ 7.12-6.95 (m, 4H), 5.07 (s, 1H), 2.63 (t, *J* = 6.4 Hz, 2H), 2.36 (t, *J* = 6.4 Hz, 2H), 2.07 (tt, *J* = 6.4, 6.4 Hz, 2H); **^{13}C{^{1}H} NMR** (100 MHz, $CDCl_3$) δ 199.3, 178.2, 160.2 (d, *J* = 245.2 Hz), 148.4 (d, *J* = 3.0 Hz), 122.7 (d, *J* = 8.8 Hz), 116.6 (d, *J* = 23.6 Hz), 105.9, 36.5, 28.3, 21.0; **HRMS** (EI, $[M]^+$) for $C_{12}H_{11}FO_2$ calcd. 206.0749, found: 206.0742.

3-(4-Chlorophenoxy)cyclohex-2-en-1-one (2c)

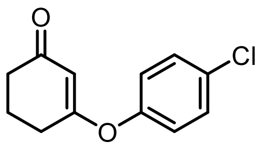

Following General Procedure B, the reaction was conducted with **1a** (1.0 mmol, 222 mg) and 4-chlorophenol (1.1 mmol, 141 mg). The crude product was purified by flash column

chromatography (hexanes/EtOAc = 8/1 to 6/1) to afford **2c** (67 mg, 30%) as yellow oil. R_f: 0.34 (hexanes/EtOAc = 4/1); **IR** (film): 2953, 2923, 1667, 1654, 1613, 1586, 1486, 1373, 1210, 1152, 1133, 1095, 1013, 835, 672 cm^{-1}; **^{1}H NMR** (400 MHz, $CDCl_3$) δ 7.35 (d, *J* = 8.8 Hz, 2H), 6.98 (d, *J* = 8.8 Hz, 2H), 5.10 (s, 1H), 2.64 (t, *J* = 6.4 Hz, 2H), 2.37 (t, *J* = 6.4 Hz, 2H), 2.07 (tt, *J* = 6.4, 6.4 Hz, 2H); **^{13}C{^{1}H} NMR** (100 MHz, $CDCl_3$) δ 199.4, 177.8, 151.1, 131.5, 130.1, 122.8, 106.2, 36.6, 28.4, 21.1; **HRMS** (EI, $[M]^+$) for $C_{12}H_{11}ClO_2$ calcd. 222.0453, found: 222.0451.

3-(4-(*tert*-Butyl)phenoxy)cyclohex-2-en-1-one (2d)

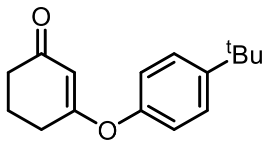

Following General Procedure B, the reaction was conducted with **1a** (1.0 mmol, 222 mg) and 4-(*tert*-butyl)phenol (1.1 mmol, 165 mg). The crude product was purified by flash column chromatography (hexanes/EtOAc = 8/1 to 6/1) to afford **2d** (132 mg, 54%) as yellow oil. R_f: 0.41 (hexanes/EtOAc = 4/1); **IR** (film): 2959, 1668, 1653, 1615, 1597, 1507, 1374, 1211, 1178, 1133, 772 cm^{-1}; **^{1}H NMR** (400 MHz, $CDCl_3$) δ 7.37 (d, *J* = 8.7 Hz, 2H), 6.93 (d, *J* = 8.7 Hz, 2H), 5.11 (s, 1H), 2.64 (t, *J* = 6.4 Hz, 2H), 2.36 (t, *J* = 6.4 Hz, 2H), 2.07 (tt, *J* = 6.4, 6.4 Hz, 2H), 1.31 (s, 9H); **^{13}C{^{1}H} NMR** (100 MHz, $CDCl_3$) δ 199.7, 178.6, 150.3, 149.1, 126.8, 120.7, 105.9, 36.6, 34.5, 31.4, 28.5, 21.2; **HRMS** (EI, $[M]^+$) for $C_{16}H_{20}O_2$ calcd. 244.1469, found: 244.1471.

3-(4-(*tert*-Pentyl)phenoxy)cyclohex-2-en-1-one (2e)

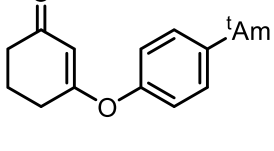

Following General Procedure B, the reaction was conducted with **1a** (1.0 mmol, 222 mg) and 4-(*tert*-pentyl)phenol (1.1 mmol, 180 mg). The crude product was purified by flash

column chromatography (hexanes/EtOAc = 8/1 to 6/1) to afford **2e** (150 mg, 58%) as brown oil. R*f*: 0.44 (hexanes/EtOAc = 4/1); **IR** (film): 2962, 1667, 1653, 1615, 1597, 1506, 1373, 1176, 1133, 836, 772 cm^{-1}; **^{1}H NMR** (400 MHz, $CDCl_3$) δ 7.30 (d, *J* = 8.8 Hz, 2H), 6.92 (d, *J* = 8.8 Hz, 2H), 5.11 (s, 1H), 2.62 (t, *J* = 6.3 Hz, 2H), 2.34 (t, *J* = 6.3 Hz, 2H), 2.05 (dd, *J* = 6.3, 6.3 Hz, 2H), 1.61 (q, *J* = 7.5 Hz, 2H), 1.25 (s, 6H), 0.66 (t, *J* = 7.5 Hz, 3H); **^{13}C{^{1}H} NMR** (100 MHz, $CDCl_3$) δ 199.8, 178.7, 150.2, 147.4, 127.4, 120.6, 105.8, 37.7, 36.8, 36.6, 28.5, 28.5, 21.2, 9.1; **HRMS** (EI, $[M]^+$) for $C_{17}H_{22}O_2$ calcd. 258.1625, found: 258.1626.

3-([1,1'-Biphenyl]-4-yloxy)cyclohex-2-en-1-one (2f)

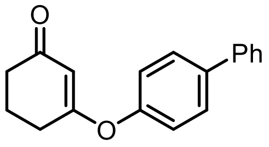

Following General Procedure B, the reaction was conducted with **1a** (1.0 mmol, 222 mg) and [1,1'-biphenyl]-4-ol (1.1 mmol, 187 mg). The crude product was purified by flash column chromatography (hexanes/EtOAc = 8/1 to 6/1) to afford **2f** (148 mg, 56%) as yellow oil. R*f*: 0.33 (hexanes/EtOAc = 4/1); **IR** (film): 1648, 1615, 1486, 1368, 1217, 1197, 1170, 1126, 769 cm^{-1}; **^{1}H NMR** (400 MHz, $CDCl_3$) δ 7.62-7.52 (m, 4H), 7.48-7.42 (m, 2H), 7.39-7.33 (m, 1H), 7.10 (d, *J* = 8.6 Hz, 2H), 5.20 (s, 1H), 2.68 (t, *J* = 6.3 Hz, 2H), 2.39 (t, *J* = 6.3 Hz, 2H), 2.10 (tt, *J* = 6.3, 6.3 Hz, 2H); **^{13}C{^{1}H} NMR** (100 MHz, $CDCl_3$) δ 199.5, 178.2, 152.0, 140.1, 139.3, 128.8, 128.7, 127.5, 127.1, 121.6, 106.1, 36.6, 28.5, 21.1; **HRMS** (EI, $[M]^+$) for $C_{18}H_{16}O_2$ calcd. 264.1156, found: 264.1150.

3-(4-Methoxyphenoxy)cyclohex-2-en-1-one (2g)

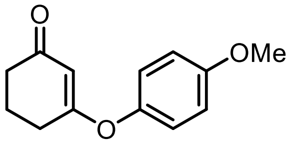

Following General Procedure B, the reaction was conducted with **1a** (1.0 mmol, 222 mg)

and 4-methoxyphenol (1.1 mmol, 136.5 mg). The crude product was purified by flash column chromatography (hexanes/EtOAc = 8/1 to 6/1) to afford **2g** (105 mg, 48%) as yellow oil [Note: The reaction by General Procedure C gave **2g** in 12% yield]. R_f: 0.25 (hexanes/EtOAc = 4/1); **IR** (film): 2949, 1599, 1505, 1456, 1426, 1376, 1330, 1298, 1247, 1208, 1192, 1135, 1101, 1033, 829, 747, 734 cm^{-1}; **^{1}H NMR** (400 MHz, $CDCl_3$) δ 6.96-6.84 (m, 4H), 5.10 (s, 1H), 3.79 (s, 3H), 2.62 (t, *J* = 6.3 Hz, 2H), 2.35 (t, *J* = 6.3 Hz, 2H), 2.06 (tt, *J* = 6.3, 6.3 Hz, 2H); **^{13}C{^{1}H} NMR** (100 MHz, $CDCl_3$) δ 199.6, 178.8, 157.4, 146.0, 122.1, 114.9, 105.7, 55.6, 36.6, 28.4, 21.1; **HRMS** (EI, $[M]^+$) for $C_{13}H_{14}O_3$ calcd. 218.0948, found: 218.0950.

3-(4-(Trifluoromethyl)phenoxy)cyclohex-2-en-1-one (2h)

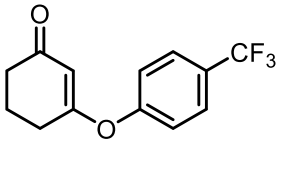

Following General Procedure C, the reaction was conducted with cyclohexane-1,3-dione (3.0 mmol, 336 mg) and **1d** (2.5 mmol, 1340 mg). The crude product was purified by flash column chromatography (hexanes/EtOAc = 8/1 to 6/1) to afford **2h** (282 mg, 44%) as brown oil [Note: The reaction by General Procedure B gave **2h** in 30% yield]. R_f: 0.36 (hexanes/EtOAc = 2/1); **IR** (film): 22954, 1661, 1622, 1604, 1373, 1321, 1214, 1171, 1123, 1102, 1063, 772 cm^{-1}; **^{1}H NMR** (400 MHz, $CDCl_3$) δ 7.66 (d, *J* = 8.3 Hz, 2H), 7.15 (d, *J* = 8.3 Hz, 2H), 5.08 (s, 1H), 2.66 (t, *J* = 6.4 Hz, 2H), 2.37 (t, *J* = 6.4 Hz, 2H), 2.08 (tt, *J* = 6.4, 6.4 Hz, 2H); **^{13}C{^{1}H} NMR** (100 MHz, $CDCl_3$) δ 199.2, 177.3, 155.2, 128.4 (q, *J* = 33.0 Hz), 127.4 (q, *J* = 3.7 Hz), 123.6 (q, *J* = 272.0 Hz), 121.9, 106.5, 36.5, 28.3, 21.0; **HRMS** (EI, $[M]^+$) for $C_{13}H_{11}F_3O_2$ calcd. 256.0717, found: 256.0710.

3-(4-Nitrophenoxy)cyclohex-2-en-1-one (2i)

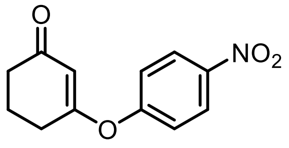

Following General Procedure C, the reaction was conducted with cyclohexane-1,3-dione (3.0 mmol, 336 mg) and **1e** (2.5 mmol, 1283 mg). The crude product was purified by flash column chromatography (hexanes/EtOAc = 8/1 to 6/1) to afford **2i** (303 mg, 52%) as brown oil [Note: The reaction by General Procedure B gave **2i** in 15% yield]. R*f*: 0.59 (hexanes/EtOAc = 2/1); **IR** (film): 2945, 2883, 1652, 1614, 1584, 1522, 1341, 1203, 1162, 1149, 1126, 863, 771 cm^{-1}; **^{1}H NMR** (400 MHz, CDCl3) δ 8.29 (d, *J* = 9.2 Hz, 2H), 7.21 (d, *J* = 9.2 Hz, 2H), 5.15 (br s, 1H), 2.68 (t, *J* = 6.3 Hz, 2H), 2.40 (t, *J* = 6.3 Hz, 2H), 2.11 (tt, *J* = 6.3, 6.3 Hz, 2H); **^{13}C{^{1}H} NMR** (100 MHz, $CDCl_3$) δ 198.9, 176.4, 157.5, 145.4, 125.9, 122.1, 107.3, 36.5, 28.3, 21.0; **HRMS** (EI, $[M]^+$) for $C_{12}H_{11}NO_4$ calcd. 233.0694, found: 233.0692.

3-(3-Methoxyphenoxy)cyclohex-2-en-1-one (2j)

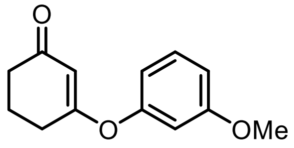

Following General Procedure B, the reaction was conducted with **1a** (1.0 mmol, 222 mg) and 3-methoxyphenol (1.1 mmol, 136 mg) following General Procedure B. The crude product was purified by flash column chromatography (hexanes/EtOAc = 8/1 to 6/1) to afford **2j** (118 mg, 54%) as yellow oil. R*f*: 0.30 (hexanes/EtOAc = 4/1); **IR** (film): 2947, 2838, 1655, 1607, 1588, 1487, 1454, 1429, 1374, 1327, 1310, 1203, 1189, 1171, 1135, 1040, 943, 865, 834, 800, 694 cm^{-1}; **^{1}H NMR** (400 MHz, $CDCl_3$) δ 7.31-7.24 (m, 1H), 6.78 (dd, *J* = 8.1, 1.8 Hz, 1H), 6.65 – 6.59 (m, 1H), 6.57 (s, 1H), 5.17 (s, 1H), 3.79 (s, 3H), 2.64 (t, *J* = 6.6 Hz, 2H), 2.37 (t, *J* = 6.6 Hz, 2H), 2.08 (tt, *J* = 6.6, 6.6 Hz, 2H); **^{13}C{^{1}H} NMR** (100 MHz, $CDCl_3$) δ 199.6, 178.1, 161.0, 153.6, 130.4, 113.4, 111.8, 107.4, 106.1, 55.5, 36.6, 28.5, 21.2; **HRMS** (EI, $[M]^+$) for $C_{13}H_{14}O_3$ calcd. 218.0948,

found: 218.0948.

3-(2-Methoxyphenoxy)cyclohex-2-en-1-one (2k)

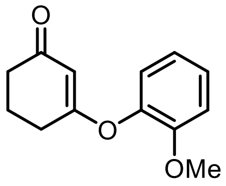

Following General Procedure B, the reaction was conducted with **1a** (1.0 mmol, 222 mg) and 2-methoxyphenol (1.1 mmol, 136 mg). The crude product was purified by flash column chromatography (hexanes/EtOAc = 8/1 to 6/1) to afford **2k** (127 mg, 58%) as yellow oil. R_f: 0.28 (hexanes/EtOAc = 4/1); **IR** (film): 2952, 2925, 2849, 1653, 1615, 1584, 1499, 1456, 1374, 1349, 1327, 1264, 1250, 1203, 1176, 1159, 1133, 1109, 1041, 1023, 765, 750 cm^{-1}; **^{1}H NMR** (400 MHz, $CDCl_3$) δ 7.19 (ddd, *J* = 8.0, 7.6, 1.8 Hz, 1H), 7.06-6.88 (m, 3H), 5.05 (s, 1H), 3.80 (s, 3H), 2.67 (t, *J* = 6.5 Hz, 2H), 2.36 (t, *J* = 6.5 Hz, 2H), 2.07 (tt, *J* = 6.5, 6.5 Hz, 2H); **^{13}C{^{1}H} NMR** (100 MHz, $CDCl_3$) δ 199.9, 178.0, 151.0, 141.2, 127.1, 122.7, 121.1, 112.8, 105.4, 55.8, 36.6, 28.2, 21.2; **HRMS** (EI, $[M]^+$) for $C_{13}H_{14}O_3$ calcd. 218.0948, found: 218.0946.

3-(4-Allyl-2-methoxyphenoxy)cyclohex-2-en-1-one (2l)

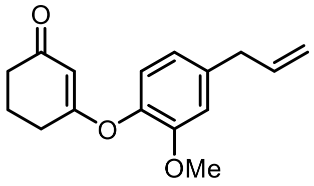

Following General Procedure B, the reaction was conducted with **1a** (1.0 mmol, 222 mg) and eugenol (1.1 mmol, 180.6 mg). The crude product was purified by flash column chromatography (hexanes/EtOAc = 8/1 to 6/1) to afford **2l** (173 mg, 67%) as yellow oil. R_f: 0.25 (hexanes/EtOAc = 4/1); **IR** (film): 2926, 1613, 1595, 1508, 1456, 1424, 1376, 1330, 1270, 1207, 1234, 1185, 1167, 1136, 1034, 913, 883, 836 cm^{-1}; **^{1}H NMR** (400 MHz, $CDCl_3$) δ 6.91 (d, *J* = 8.0 Hz, 1H), 6.79-6.72 (m, 2H), 5.94 (m, 1H), 5.15-5.02 (m, 3H), 3.78 (s, 3H), 3.36 (d, *J* = 6.7 Hz, 2H), 2.65 (t, *J* = 6.3 Hz, 2H), 2.36 (t, *J* = 6.3 Hz,

2H), 2.06 (tt, J = 6.3, 6.3 Hz, 2H); **$^{13}C\{^1H\}$ NMR** (100 MHz, $CDCl_3$) δ 199.9, 178.2, 150.8, 139.4, 139.3, 136.9, 122.4, 121.0, 116.3, 113.0, 105.4, 55.7, 40.0, 36.6, 28.2, 21.2; **HRMS** (EI, $[M]^+$) for $C_{16}H_{18}O_3$ calcd. 258.1261, found: 258.1265.

3-Methoxy-4-((3-oxocyclohex-1-en-1-yl)oxy)benzaldehyde (2m)

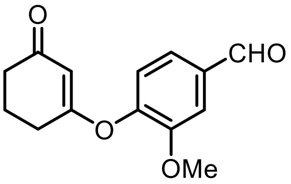

Following General Procedure B, the reaction was conducted with **1a** (1.0 mmol, 222 mg) and vanillin (1.1 mmol, 167 mg). The crude product was purified by flash column chromatography (hexanes/EtOAc = 8/1 to 6/1) to afford **2m** (94 mg, 38%) as yellow oil. R_f: 0.24 (hexanes/EtOAc = 2/1); **IR** (film): 2951, 2852, 2731, 1696, 1652, 1617, 1592, 1501, 1463, 1421, 1373, 1273, 1205, 1158, 1133, 1029, 865, 831, 780, 732 cm^{-1}; **^1H NMR** (400 MHz, $CDCl_3$) δ 9.95 (s, 1H), 7.52-7.46 (m, 2H), 7.20 (d, J = 7.9 Hz, 1H), 5.03 (s, 1H), 3.89 (s, 3H), 2.69 (t, J = 6.4 Hz, 2H), 2.38 (t, J = 6.4 Hz, 2H), 2.09 (tt, J = 6.4, 6.4 Hz, 2H); **$^{13}C\{^1H\}$ NMR** (100 MHz, $CDCl_3$) δ 199.5, 190.7, 177.0, 151.9, 146.3, 135.3, 124.9, 123.2, 111.3, 106.0, 56.1, 36.6, 28.0, 21.1; **HRMS** (EI, $[M]^+$) for $C_{14}H_{14}O_4$ calcd. 246.0898, found: 246.0896.

3-(3,5-Dimethylphenoxy)cyclohex-2-en-1-one (2n)

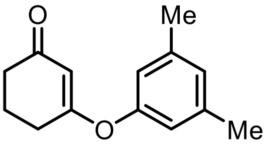

Following General Procedure B, the reaction was conducted with **1a** (1.0 mmol, 222 mg) and 3,5-dimethylphenol (1.1 mmol, 134 mg). The crude product was purified by flash column chromatography (hexanes/EtOAc = 8/1 to 6/1) to afford **2n** (96 mg, 45%) as yellow oil. R_f: 0.41 (hexanes/EtOAc = 4/1); **IR** (film): 2948, 2920, 2872, 1638, 1615, 1595, 1584, 1455, 1373, 1317, 1291, 1208, 1174, 1153, 1135, 1020, 945, 865, 833, 690,

656 cm^{-1}; **^{1}H NMR** (400 MHz, $CDCl_3$) δ 6.85 (s, 1H), 6.63 (s, 2H), 5.13 (s, 1H), 2.62 (t, *J* = 6.4 Hz, 2H), 2.36 (t, *J* = 6.4 Hz, 2H), 2.29 (s, 6H), 2.06 (tt, *J* = 6.4, 6.4 Hz, 2H); **^{13}C{^{1}H} NMR** (100 MHz, $CDCl_3$) δ 199.7, 178.4, 152.5, 139.8, 127.6, 118.8, 105.9, 36.6, 28.5, 21.2, 21.1; **HRMS** (EI, $[M]^+$) for $C_{14}H_{16}O_2$ calcd. 216.1156, found: 216.1153.

3-(Naphthalen-1-yloxy)cyclohex-2-en-1-one (2o)

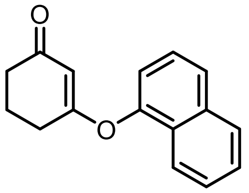

Following General Procedure B, the reaction was conducted with **1a** (1.0 mmol, 222 mg) and naphthalen-1-ol (1.1 mmol, 159 mg). The crude product was purified by flash column chromatography (hexanes/EtOAc = 8/1 to 6/1) to afford **2o** (100 mg, 42%) as brown solid. m.p. 85-86 °C; R_f: 0.39 (hexanes/EtOAc = 4/1); **IR** (film): 1652, 1614, 1372, 1221, 1170, 773 cm^{-1}; **^{1}H NMR** (400 MHz, $CDCl_3$) δ 7.93-7.85 (m, 1H), 7.82-7.72 (m, 2H), 7.57-7.40 (m, 3H), 7.17 (d, *J* = 7.5 Hz, 1H), 5.02 (s, 1H), 2.83 (t, *J* = 6.3 Hz, 2H), 2.39 (t, *J* = 6.6 Hz, 2H), 2.15 (tt, *J* = 6.4, 6.4 Hz, 2H); **^{13}C{^{1}H} NMR** (100 MHz, $CDCl_3$) δ 199.5, 178.3, 148.6, 135.0, 128.2, 126.8, 126.7, 126.4, 126.3, 125.5, 121.1, 117.8, 106.4, 36.6, 28.3, 21.3; **HRMS** (EI, $[M]^+$) for $C_{16}H_{14}O_2$ calcd. 238.0999, found: 238.0999.

3-(Naphthalen-2-yloxy)cyclohex-2-en-1-one (2p)

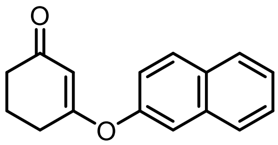

Following General Procedure B, the reaction was conducted with **1a** (1 mmol, 222 mg) and naphthalen-2-ol (1.1 mmol, 159 mg). The crude product was purified by flash column chromatography (hexanes/EtOAc = 8/1 to 6/1) to afford **2p** (114 mg, 48%) as yellow solid. m.p. 70-71 °C; R_f: 0.39 (hexanes/EtOAc = 4/1); **IR** (film): 2953, 2922,

2869, 1654, 1611, 1597, 1373, 1211, 1173, 1133, 959, 753 cm^{-1}; **^{1}H NMR** (400 MHz, $CDCl_3$) δ 7.90-7.82 (m, 2H), 7.81-7.76 (m, 1H), 7.54-7.46 (m, 3H), 7.18 (dd, *J* = 8.9, 2.3 Hz, 1H), 5.16 (s, 1H), 2.71 (t, *J* = 6.3 Hz, 2H), 2.39 (t, *J* = 6.3 Hz, 2H), 2.12 (tt, *J* = 6.3, 6.3 Hz, 2H); **^{13}C{^{1}H} NMR** (100 MHz, $CDCl_3$) δ 199.5, 178.2, 150.2, 133.9, 131.5, 130.2, 127.8, 127.5, 126.9, 126.0, 120.6, 118.4, 106.4, 36.6, 28.6, 21.2; **HRMS** (EI, $[M]^+$) for $C_{16}H_{14}O_2$ calcd. 238.0999, found: 238.0992.

3-(*p*-Tolyloxy)cyclohex-2-en-1-one (2q)

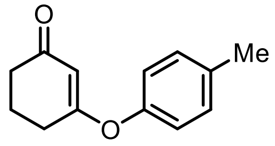

Following General Procedure C, the reaction was conducted with cyclohexane-1,3-dione (3.0 mmol, 336 mg) and **1f** (2.5 mmol, 1205 mg). The crude product was purified by flash column chromatography (hexanes/EtOAc = 8/1 to 6/1) to afford **2q** (253 mg, 50%) as yellow oil. R_f: 0.36 (hexanes/EtOAc = 4/1); **IR** (film): 1654, 1617, 1506, 1374, 1218, 772 cm^{-1}; **^{1}H NMR** (400 MHz, $CDCl_3$) δ 7.15 (d, *J* = 8.3 Hz, 2H), 6.88 (d, *J* = 8.3 Hz, 2H), 5.10 (s, 1H), 2.61 (t, *J* = 6.4 Hz, 2H), 2.35 (t, *J* = 6.4 Hz, 2H), 2.32 (s, 3H), 2.04 (tt, *J* = 6.4, 6.4 Hz, 2H); **^{13}C{^{1}H} NMR** (100 MHz, $CDCl_3$) δ 199.5, 178.4, 150.3, 135.7, 130.3, 120.9, 105.8, 36.5, 28.4, 21.1, 20.7; **HRMS** (EI, $[M]^+$) for $C_{13}H_{14}O_2$ calcd. 202.0999, found: 202.0990.

5-Methyl-3-phenoxycyclohex-2-en-1-one (2r)

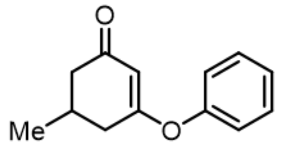

Following General Procedure C, the reaction was conducted with 5-methylcyclohexane-1,3-dione (3.0 mmol, 378 mg) and diphenyliodonium hexafluorophosphate (2.5 mmol, 1065 mg). The crude product was purified by flash column chromatography

(hexanes/EtOAc = 8/1 to 6/1) to afford **2r** (217 mg, 43%) as yellow oil. R*f*: 0.45 (hexanes/EtOAc = 4/1); **IR** (film): 2955, 2924, 1654, 1613, 1586, 1488, 1456, 1376, 1337, 1203, 1135, 768, 694 cm^{-1}; **^{1}H NMR** (400 MHz, $CDCl_3$) δ 7.38 (dd, *J* = 7.9, 7.9 Hz, 2H), 7.23 (t, *J* = 7.4 Hz, 1H), 7.05-6.99 (m, 2H), 5.10 (s, 1H), 2.72-2.62 (m, 1H), 2.49-2.26 (m, 3H), 2.13-2.02 (m, 1H), 1.14 (d, *J* = 6.3 Hz, 3H); **^{13}C{^{1}H} NMR** (100 MHz, $CDCl_3$) δ 199.5, 177.7, 152.8, 130.0, 126.1, 121.3, 105.7, 45.0, 36.6, 28.9, 20.9; **HRMS** (EI, $[M]^+$) for $C_{13}H_{14}O_2$ calcd. 202.0999, found: 202.0996.

5,5-Dimethyl-3-phenoxycyclohex-2-en-1-one (2s)[6]

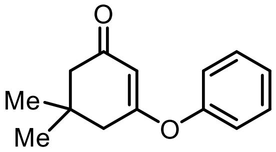

Following General Procedure C, the reaction was conducted with 5,5-dimethylcyclohexane-1,3-dione (3.0 mmol, 420 mg) and diphenyliodonium hexafluorophosphate (2.5 mmol, 1065 mg). The crude product was purified by flash column chromatography (hexanes/EtOAc = 8/1 to 6/1) to afford **2s** (275 mg, 51%) as yellow oil. R*f*: 0.5 (hexanes/EtOAc = 4/1); **^{1}H NMR** (400 MHz, $CDCl_3$) δ 7.37 (dd, *J* = 7.7, 7.7 Hz, 2H), 7.22 (t, *J* = 7.7 Hz, 1H), 7.01 (d, *J* = 7.7 Hz, 2H), 5.11 (s, 1H), 2.51 (s, 2H), 2.23 (s, 2H), 1.14 (s, 6H).

3,4-Dihydrodibenzo[b,d]furan-1(2H)-one (3a)[7]

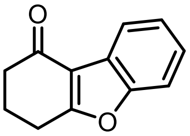

Following General Procedure D, the reaction was conducted with **2a** (0.5 mmol, 94 mg). The crude product was purified by flash column chromatography (hexanes/EtOAc = 10/1

[6] Xu, Z.-F.; Cai, C.-X.; Jiang, M.; Liu, J.-T. *Org. Lett.* **2014**, *16*, 3436–3439.

[7] Ma, D.; Cai, Q.; Xie, X. *Synlett.* **2005**, *11*, 1767–1770.

to 8/1) to afford **3a** (69 mg, 74%) as brown oil. R_f: 0.38 (hexanes/EtOAc = 4/1); **^{1}H NMR** (400 MHz, $CDCl_3$) δ 8.12-7.97 (m, 1H), 7.50-7.40 (m, 1H), 7.36-7.27 (m, 2H), 3.02 (t, *J* = 6.3 Hz, 2H), 2.60 (t, *J* = 6.3 Hz, 2H), 2.27 (tt, *J* = 6.3, 6.3 Hz, 2H). For comparison, the reaction with 1.0 mmol of **2a** (188 mg) gave **3a** (130 mg) in 70% yield (Note: The molar equivalents of reagents, the solvent concentration, reaction temperature, and the reaction time follow the General Procedure D). In the microwave-irradiated experiment (see Scheme 3b), the reaction with 0.5 mmol of **2a** was conducted in a sealed vessel and stirred at 100 °C for 3 hours (Note: the reaction temperature was monitored by an internal probe).

8-Fluoro-3,4-dihydrodibenzo[b,d]furan-1(2H)-one (3b)

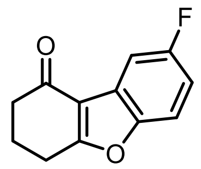

Following General Procedure D, the reaction was conducted with **2b** (0.5 mmol, 103 mg). The crude product was purified by flash column chromatography (hexanes/EtOAc = 10/1 to 8/1) to afford **3b** (66 mg, 65%) as brown solid. m.p. 84-85 °C; R_f: 0.34 (hexanes/EtOAc = 4/1); **IR** (cast): 2924, 2652, 1588, 1482, 1451, 1217, 1167, 771, 751 cm^{-1}; **^{1}H NMR** (400 MHz, $CDCl_3$) δ 7.72 (dd, *J* = 8.3, 2.7 Hz, 1H), 7.40 (dd, *J* = 9.0, 4.0 Hz, 1H), 7.02 (ddd, *J* = 9.0, 9.0, 2.7 Hz, 1H), 3.04 (t, *J* = 6.4 Hz, 2H), 2.61 (t, *J* = 6.4 Hz, 2H), 2.28 (tt, *J* = 6.4, 6.4 Hz, 2H); **^{13}C{^{1}H} NMR** (100 MHz, $CDCl_3$) δ 194.4, 172.2, 160.2 (d, *J* = 240.7 Hz), 150.7, 124.7 (d, *J* = 11.4 Hz), 116.7, 112.5 (d, *J* = 26.4 Hz), 111.8 (d, *J* = 9.6 Hz), 107.8 (d, *J* = 26.0 Hz), 37.7, 23.9, 22.4; **HRMS** (EI, $[M]^+$) for $C_{12}H_9FO_2$ calcd. 204.0592, found: 204.0590.

8-Chloro-3,4-dihydrodibenzo[b,d]furan-1(2H)-one (3c)

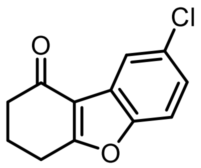

Following General Procedure D, the reaction was conducted with **2c** (0.5 mmol, 111 mg). The crude product was purified by flash column chromatography (hexanes/EtOAc = 10/1 to 8/1) to afford **3c** (72 mg, 65%) as brown solid. m.p. 98-99 °C; R_f: 0.36 (hexanes/EtOAc = 4/1); **IR** (cast): 3505, 2954, 1652, 1618, 1588, 1462, 1442, 1430, 1395, 1355, 1316, 1248, 1189, 1168, 1058, 1044, 1010, 883, 815, 752, 728, 619 cm^{-1}; **^{1}H NMR** (400 MHz, $CDCl_3$) δ 8.03 (dd, *J* = 2.2, 0.6 Hz, 1H), 7.38 (dd, *J* = 8.8, 0.6 Hz, 1H), 7.27 (dd, *J* = 8.8, 2.2 Hz, 1H), 3.04 (t, *J* = 6.4 Hz, 2H), 2.60 (t, *J* = 6.4 Hz, 2H), 2.28 (tt, *J* = 6.4, 6.4 Hz, 2H); **^{13}C{^{1}H} NMR** (100 MHz, $CDCl_3$) δ 194.3, 171.9, 152.9, 130.2, 125.2, 125.1, 121.5, 116.2, 112.0, 37.7, 23.8, 22.3; **HRMS** (EI, $[M]^+$) for $C_{12}H_9ClO_2$ calcd. 220.0297, found: 220.0295.

8-(*tert*-Butyl)-3,4-dihydrodibenzo[b,d]furan-1(2H)-one (3d)

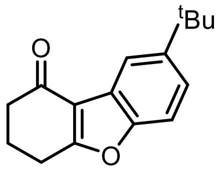

Following General Procedure D, the reaction was conducted with **2d** (0.5 mmol, 122 mg). The crude product was purified by flash column chromatography (hexanes/EtOAc = 10/1 to 8/1) to afford **3d** (82 mg, 68%) as brown solid. m.p. 58-59 °C; R_f: 0.46 (hexanes/EtOAc = 4/1); **IR** (cast): 2956, 1673, 1653, 1558, 1540, 1507, 1457, 1394, 1363, 1219, 1192, 1174, 772 cm^{-1}; **^{1}H NMR** (400 MHz, $CDCl_3$) δ 8.07 (s, 1H), 7.37 (s, 2H), 3.01 (t, *J* = 6.3 Hz, 2H), 2.59 (t, *J* = 6.3 Hz, 2H), 2.26 (tt, *J* = 6.3, 6.3 Hz, 2H), 1.38 (s, 9H); **^{13}C{^{1}H} NMR** (100 MHz, $CDCl_3$) δ 194.9, 171.0, 152.7, 147.8, 123.4, 122.6, 118.1, 116.5, 110.2, 37.9, 34.9, 31.8, 23.9, 22.5; **HRMS** (EI, $[M]^+$) for $C_{16}H_{18}O_2$ calcd. 242.1312, found: 242.1313.

8-(*tert*-Pentyl)-3,4-dihydrodibenzo[b,d]furan-1(2H)-one (3e)

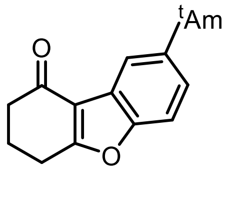

Following General Procedure D, the reaction was conducted with **2e** (0.5 mmol, 129 mg). The crude product was purified by flash column chromatography (hexanes/EtOAc = 10/1 to 8/1) to afford **3e** (85 mg, 66%) as brown solid. m.p. 69-70 °C; R*f*: 0.47 (hexanes/EtOAc = 4/1); **IR** (cast): 2962, 1674, 1591, 1461, 1395, 1193, 1173, 1056, 1006, 811, 774 cm^{-1}; **^{1}H NMR** (400 MHz, $CDCl_3$) δ 8.01 (s, 1H), 7.38 (d, *J* = 8.7 Hz, 1H), 7.32-7.27 (m, 1H), 3.01 (t, *J* = 6.3 Hz, 2H), 2.60 (t, *J* = 6.3 Hz, 2H), 2.26 (tt, *J* = 6.3, 6.3 Hz, 2H), 1.70 (q, *J* = 7.4 Hz, 2H), 1.35 (s, 6H), 0.66 (t, *J* = 7.4 Hz, 3H). **^{13}C{^{1}H} NMR** (100 MHz, $CDCl_3$) δ 194.9, 171.0, 152.7, 146.1, 123.4, 123.2, 119.0, 116.6, 110.2, 38.1, 37.9, 37.2, 29.0, 23.9, 22.5, 9.2; **HRMS** (EI, $[M]^+$) for $C_{17}H_{20}O_2$ calcd. 256.1469, found: 256.1464.

8-Phenyl-3,4-dihydrodibenzo[b,d]furan-1(2H)-one (3f)

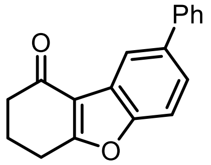

Following General Procedure D, the reaction was conducted with **2f** (0.5 mmol, 132 mg). The crude product was purified by flash column chromatography (hexanes/EtOAc = 10/1 to 8/1) to afford **3f** (85 mg, 65%) as yellow solid. m.p. 143-144 °C; R*f*: 0.37 (hexanes/EtOAc = 4/1); **IR** (cast): 2954, 2923, 2869, 1671, 1591, 1458, 1396, 1377, 1192, 1165, 1004, 890, 765, 698 cm^{-1}; **^{1}H NMR** (400 MHz, $CDCl_3$) δ 8.31-8.24 (m, 1H), 7.65 (dd, *J* = 7.4, 7.4 Hz, 2H), 7.58-7.49 (m, 2H), 7.45 (dd, *J* = 7.4, 7.4 Hz, 2H), 7.35 (t, *J* = 7.4 Hz, 1H), 3.05 (t, *J* = 6.3 Hz, 2H), 2.63 (t, *J* = 6.3 Hz, 2H), 2.30 (tt, *J* = 6.3 Hz, 2H); **^{13}C{^{1}H} NMR** (100 MHz, $CDCl_3$) δ 194.7, 171.4, 154.1, 141.0, 138.1, 128.7, 127.5, 127.1, 124.4, 124.2, 120.2, 116.6, 111.1, 37.9, 23.9, 22.4; **HRMS** (EI, $[M]^+$) for $C_{18}H_{14}O_2$ calcd. 262.0999, found: 262.0997.

8-Methoxy-3,4-dihydrodibenzo[b,d]furan-1(2H)-one (3g)

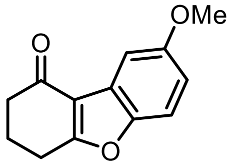

Following General Procedure D, the reaction was conducted with **2g** (0.5 mmol, 109 mg). The crude product was purified by flash column chromatography (hexanes/EtOAc = 10/1 to 8/1) to afford **3g** (63 mg, 58%) as brown solid. m.p. 86-87 °C; R*f*: 0.32 (hexanes/EtOAc = 4/1); **IR** (cast): 2931, 1669, 1619, 1587, 1590, 1482, 1459, 1435, 1398, 1274, 1252, 1227, 1173, 1154, 1136, 1058, 1029, 1008, 806, 785 cm^{-1}; **^{1}H NMR** (400 MHz, $CDCl_3$) δ 7.53 (d, *J* = 2.7 Hz, 1H), 7.35 (d, *J* = 9.0 Hz, 1H), 6.89 (dd, *J* = 9.0, 2.7 Hz, 1H), 3.87 (s, 3H), 3.02 (t, *J* = 6.3 Hz, 2H), 2.60 (t, *J* = 6.3 Hz, 2H), 2.27 (tt, *J* = 6.3 Hz, 2H); **^{13}C{^{1}H} NMR** (100 MHz, CDCl3) δ 194.9, 171.4, 157.2, 149.3, 124.4, 116.7, 113.9, 111.6, 103.8, 56.0, 37.9, 23.9, 22.5; **HRMS** (EI, $[M]^+$) for $C_{13}H_{12}O_3$ calcd. 216.0792, found: 216.0816.

8-(Trifluoromethyl)-3,4-dihydrodibenzo[b,d]furan-1(2H)-one (3h)

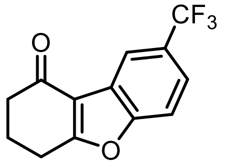

Following General Procedure D, the reaction was conducted with **2h** (0.5 mmol, 128 mg). The crude product was purified by flash column chromatography (hexanes/EtOAc = 10/1 to 8/1) to afford **3h** (76 mg, 60%) as brown solid. m.p. 109-110 °C; R*f*: 0.33 (hexanes/EtOAc = 4/1); **IR** (cast): 2962, 1671, 1326, 1314, 1152, 1116, 1007, 772 cm^{-1}; **^{1}H NMR** (400 MHz, $CDCl_3$) δ 8.34 (s, 1H), 7.63-7.47 (m, 2H), 3.07 (t, *J* = 6.3 Hz, 2H), 2.62 (t, *J* = 6.3 Hz, 2H), 2.30 (tt, *J* = 6.3, 6.3 Hz, 2H); **^{13}C{^{1}H} NMR** (100 MHz, CDCl3) δ 194.2, 172.2, 155.8, 127.1 (q, *J* = 32.4 Hz), 124.3 (q, *J* = 272.3 Hz), 124.0, 122.2 (q, *J* = 3.7 Hz), 119.5 (q, *J* = 4.1 Hz), 116.4, 111.5, 37.7, 23.7, 22.3; **HRMS** (EI, $[M]^+$) for $C_{13}H_9F_3O_2$ calcd. 254.0560, found: 2540553.

8-Nitro-3,4-dihydrodibenzo[b,d]furan-1(2H)-one (3i)

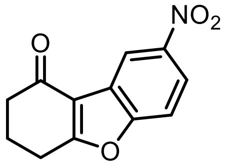

Following General Procedure D, the reaction was conducted with **2i** (0.5 mmol, 116 mg). The crude product was purified by flash column chromatography (hexanes/EtOAc = 10/1 to 8/1) to afford **3i** (71 mg, 61%) as brick red soild. m.p. 182-183 °C; R*f*: 0.18 (hexanes/EtOAc = 4/1); **IR** (cast): 1683, 1522, 1342, 1219, 772 cm^{-1}; **^{1}H NMR** (400 MHz, $CDCl_3$) δ 8.90 (d, *J* = 2.1 Hz, 1H), 8.24 (dd, *J* = 9.0, 2.1 Hz, 1H), 7.56 (d, *J* = 9.0 Hz, 1H), 3.10 (t, *J* = 6.3 Hz, 2H), 2.65 (t, *J* = 6.3 Hz, 2H), 2.33 (tt, *J* = 6.3, 6.3 Hz, 2H); **$^{13}C\{^1H\}$ NMR** (100 MHz, $CDCl_3$) δ 193.7, 173.3, 157.1, 145.3, 124.4, 120.9, 118.1, 116.7, 111.6, 37.6, 23.8, 22.2; **HRMS** (EI, $[M]^+$) for $C_{12}H_9NO_4$ calcd. 231.0537, found: 231.0531.

7-Methoxy-3,4-dihydrodibenzo[b,d]furan-1(2H)-one (3j)

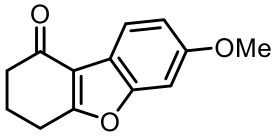

Following General Procedure D, the reaction was conducted with **2j** (0.5 mmol, 109 mg). The crude product was purified by flash column chromatography (hexanes/EtOAc = 10/1 to 8/1) to afford **3j** (59 mg, 55%) as brown solid. m.p. 76-77 °C; R*f*: 0.33 (hexanes/EtOAc = 4/1); **IR** (cast): 2947, 1665, 1625, 1590, 1496, 1455, 1426, 1407, 1363, 1274, 1136, 1101, 1113, 1056, 1043, 1028, 1002, 931, 825 cm^{-1}; **^{1}H NMR** (400 MHz, $CDCl_3$) δ 7.89 (d, *J* = 8.5 Hz, 1H), 7.00 (s, 1H), 6.93 (d, *J* = 8.5 Hz, 1H), 3.85 (s, 3H), 3.00 (t, *J* = 6.2 Hz, 2H), 2.58 (t, *J* = 6.2 Hz, 2H), 2.25 (tt, *J* = 6.2, 6.2 Hz, 2H); **$^{13}C\{^1H\}$ NMR** (100 MHz, $CDCl_3$) δ 194.8, 170.0, 158.3, 155.6, 121.8, 116.9, 116.5, 112.5, 96.3, 55.7, 37.8, 23.7, 22.5; **HRMS** (EI, $[M]^+$) for $C_{13}H_{12}O_3$ calcd. 216.0792, found: 216.0790.

6-Methoxy-3,4-dihydrodibenzo[b,d]furan-1(2H)-one (3k)

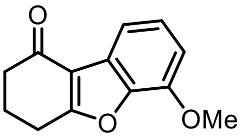

Following General Procedure D, the reaction was conducted with **2k** (0.5 mmol, 109 mg). The crude product was purified by flash column chromatography (hexanes/EtOAc = 10/1 to 8/1) to afford **3k** (67 mg, 62%) as brown solid. m.p. 43-44 °C; R_f: 0.32 (hexanes/EtOAc = 4/1); **IR** (cast): 2954, 2925, 1671, 1655, 1616, 1497, 1455, 1374, 1278, 1263, 1203, 1176, 1134, 1003, 766 cm^{-1}; **^{1}H NMR** (400 MHz, $CDCl_3$) δ 7.65 (d, *J* = 7.9 Hz, 1H), 7.25 (dd, *J* = 7.9, 8.0 Hz, 1H), 6.85 (d, *J* = 8.0 Hz, 1H), 4.02 (s, 3H), 3.06 (t, *J* = 6.4 Hz, 2H), 2.61 (t, *J* = 6.4 Hz, 2H), 2.28 (tt, *J* = 6.4, 6.4 Hz, 2H); **^{13}C{^{1}H} NMR** (100 MHz, $CDCl_3$) δ 194.8, 170.6, 144.9, 143.6, 125.4, 125.3, 116.8, 114.0, 107.3, 56.1, 37.9, 23.8, 22.5; **HRMS** (EI, $[M]^+$) for $C_{13}H_{12}O_3$ calcd. 216.0792, found: 216.0785.

4-Methoxy-9-oxo-6,7,8,9-tetrahydrodibenzo[b,d]furan-2-carbaldehyde (3m)

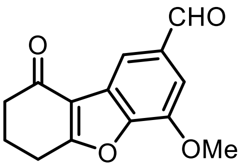

Following General Procedure D, the reaction was conducted with **2m** (0.5 mmol, 123 mg). The crude product was purified by flash column chromatography (hexanes/EtOAc = 10/1 to 8/1) to afford **3m** (61 mg, 50%) as white solid. m.p. 210-211 °C; R_f: 0.26 (hexanes/EtOAc = 4/1); **IR** (cast): 2952, 2922, 1694, 1675, 1620, 1595, 1485, 1460, 1438, 1403, 1295, 1127, 1000, 905 cm^{-1}; **^{1}H NMR** (400 MHz, $CDCl_3$) δ 10.04 (s, 1H), 8.16 (s, 1H), 7.43 (s, 1H), 4.07 (d, *J* = 0.0 Hz, 3H), 3.10 (t, *J* = 6.2 Hz, 2H), 2.65 (t, *J* = 6.3 Hz, 2H), 2.32 (p, *J* = 6.4 Hz, 2H); **^{13}C{^{1}H} NMR** (100 MHz, $CDCl_3$) δ 194.4, 191.7, 171.9, 147.0, 145.8, 134.8, 125.5, 120.2, 117.0, 104.8, 56.3, 37.8, 23.8, 22.3; **HRMS** (EI, $[M]^+$) for $C_{14}H_{12}O_4$ calcd. 244.0741, found: 244.0738.

7,9-Dimethyl-3,4-dihydrodibenzo[b,d]furan-1(2H)-one (3n)

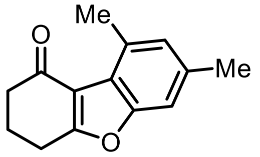

Following General Procedure D, the reaction was conducted with **2n** (0.5 mmol, 108 mg). The crude product was purified by flash column chromatography (hexanes/EtOAc = 10/1 to 8/1) to afford **3n** (76 mg, 71%) as brown solid. m.p. 61-62 °C; R_f: 0.50 (hexanes/EtOAc = 4/1); **IR** (cast): 2956, 2923, 1674, 1573, 1494, 1455, 1403, 1381, 1291, 1248, 1168, 1063, 1049, 1002, 834, 802 cm^{-1}; **^{1}H NMR** (400 MHz, $CDCl_3$) δ 7.07 (s, 1H), 6.91 (s, 1H), 3.00 (t, *J* = 6.4 Hz, 2H), 2.81 (s, 3H), 2.60 (t, *J* = 6.4 Hz, 2H), 2.41 (s, 3H), 2.22 (tt, *J* = 6.4, 6.4 Hz, 2H); **$^{13}C\{^1H\}$ NMR** (100 MHz, $CDCl_3$) δ 193.6, 170.3, 155.3, 135.4, 133.0, 127.4, 120.7, 117.6, 108.6, 38.8, 24.2, 22.1, 22.0, 21.4 **HRMS** (EI, $[M]^+$) for $C_{14}H_{14}O_2$ calcd. 214.0993, found: 214.0995.

9,10-Dihydronaphtho[1,2-b]benzofuran-7(8H)-one (3o)

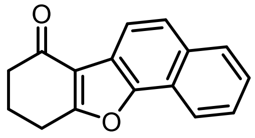

Following General Procedure D, the reaction was conducted with **2o** (0.5 mmol, 119 mg). The crude product was purified by flash column chromatography (hexanes/EtOAc = 10/1 to 8/1) to afford **3o** (66 mg, 56%) as brown solid. m.p. 138-139 °C; R_f: 0.63 (hexanes/EtOAc = 4/1); **IR** (cast): 2953, 2923, 2868, 1672, 1585, 1526, 1465, 1406, 1371, 1163, 1080, 1057, 1004, 815, 748, 682 cm^{-1}; **^{1}H NMR** (400 MHz, $CDCl_3$) δ 8.26 (d, *J* = 8.3 Hz, 1H), 8.13 (d, *J* = 8.5 Hz, 1H), 7.95 (d, *J* = 8.3 Hz, 1H), 7.76 (d, *J* = 8.5 Hz, 1H), 7.64-7.56 (m, 1H), 7.55-7.47 (m, 1H), 3.16 (t, *J* = 6.4 Hz, 2H), 2.66 (t, *J* = 6.4 Hz, 2H), 2.33 (tt, *J* = 6.4, 6.4 Hz, 2H); **$^{13}C\{^1H\}$ NMR** (100 MHz, $CDCl_3$) δ 195.1, 169.5, 150.0, 131.7, 128.5, 126.6, 125.5, 125.0, 120.8, 119.7, 119.7, 119.6, 117.5 38.0, 24.0, 22.7;

HRMS (EI, $[M]^+$) for $C_{16}H_{12}O_2$ calcd. 236.0843, found: 236.0838.

9,10-Dihydronaphtho[2,1-b]benzofuran-11(8H)-one (3p)

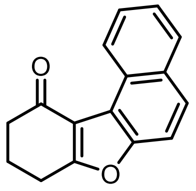

Following General Procedure D, the reaction was conducted with **2p** (0.5 mmol, 119 mg). The crude product was purified by flash column chromatography (hexanes/EtOAc = 10/1 to 8/1) to afford **3p** (106 mg, 90%) as brown solid. m.p. 151-152 °C; R_f: 0.71 (hexanes/EtOAc = 4/1); **IR** (film): 2953, 2924, 2889, 1668, 1624, 1566, 1523, 1455, 1425, 1416, 1391, 1364, 1220, 1065, 1005, 806, 754, 718 cm^{-1}; **^{1}H NMR** (400 MHz, $CDCl_3$) δ 9.63 (d, J = 8.4 Hz, 1H), 7.92 (d, J = 8.1 Hz, 1H), 7.79 (d, J = 8.9 Hz, 1H), 7.71-7.58 (m, 2H), 7.53 (dd, J = 7.4, 7.4 Hz, 1H), 3.15 (t, J = 6.4 Hz, 2H), 2.74 (t, J = 6.4 Hz, 2H), 2.32 (tt, J = 6.4, 6.4 Hz, 2H); **^{13}C{^{1}H} NMR** (100 MHz, $CDCl_3$) δ 194.1, 170.1, 152.3, 131.2, 128.2, 128.2, 128.1, 127.0, 126.6, 125.2, 119.6, 119.0, 111.6, 39.0, 24.4, 22.2; **HRMS** (EI, $[M]^+$) for $C_{16}H_{12}O_2$ calcd. 236.0843, found: 236.0838.

8-Methyl-3,4-dihydrodibenzo[b,d]furan-1(2H)-one (3q)

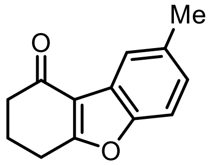

Following General Procedure D, the reaction was conducted with **2q** (0.5 mmol, 101 mg). The crude product was purified by flash column chromatography (hexanes/EtOAc = 10/1 to 8/1) to afford **3q** (67 mg, 67%) as brown solid. m.p. 90-91 °C; R_f: 0.40 (hexanes/EtOAc = 4/1); **IR** (cast): 2951, 1666, 1588, 1457, 1394, 1179, 1004, 800, 775 cm^{-1}; **^{1}H NMR** (400 MHz, $CDCl_3$) δ 7.87 – 7.84 (m, 1H), 7.33 (d, J = 8.4 Hz, 1H), 7.11 (dd, J = 8.4, 1.4 Hz, 1H), 3.01 (t, J = 6.3 Hz, 2H), 2.60 (t, J = 6.4 Hz, 2H), 2.45 (s, 3H), 2.26 (p, J = 6.4 Hz, 2H); **^{13}C{^{1}H} NMR** (100 MHz, $CDCl_3$) δ 194.9, 171.0, 153.0, 134.2, 126.0, 123.7,

121.7, 116.3, 110.5, 37.8, 23.8, 22.5, 21.3; **HRMS** (EI, $[M]^+$) for $C_{13}H_{12}O_2$ calcd. 200.0843, found: 200.0841.

3-Methyl-3,4-dihydrodibenzo[b,d]furan-1(2H)-one (3r)

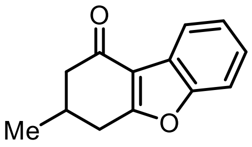

Following General Procedure D, the reaction was conducted with **2r** (0.5 mmol, 101 mg). The crude product was purified by flash column chromatography (hexanes/EtOAc = 10/1 to 8/1) to afford **3r** (65 mg, 65%) as yellow solid. m.p. 108-109 °C; R_f: 0.50 (hexanes/EtOAc = 4/1); **IR** (cast): 2962, 1668, 1592, 1483, 1449, 1042, 772, 749 cm^{-1}; **^{1}H NMR** (400 MHz, $CDCl_3$) δ 8.09-8.01 (m, 1H), 7.52-7.43 (m, 1H), 7.35-7.28 (m, 2H), 3.10 (m, 1H), 2.78-2.61 (m, 2H), 2.61-2.48 (m, 1H), 2.42-2.30 (m, 1H), 1.23 (d, J = 6.6 Hz, 3H); **^{13}C{^{1}H} NMR** (100 MHz, $CDCl_3$) δ 194.3, 170.5, 154.7, 124.9, 124.4, 123.6, 121.7, 116.1, 111.1, 46.3, 31.8, 30.7, 21.1; **HRMS** (EI, $[M]^+$) for $C_{13}H_{12}O_2$ calcd. 200.0843, found: 200.0837.

3,3-Dimethyl-3,4-dihydrodibenzo[b,d]furan-1(2H)-one (3s)[8]

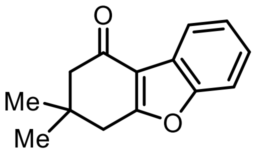

Following General Procedure D, the reaction was conducted with **2s** (0.5 mmol, 108 mg). The crude product was purified by flash column chromatography (hexanes/EtOAc = 10/1 to 8/1) to afford **3s** (65 mg, 61%) as yellow solid. m.p. 108-109 °C; R_f: 0.55 (hexanes/EtOAc = 4/1); **^{1}H NMR** (400 MHz, $CDCl_3$) δ 8.07-8.02 (m, 1H), 7.50-7.44 (m, 1H), 7.37-7.27 (m, 2H), 2.90 (s, 2H), 2.48 (s, 2H), 1.20 (s, 6H).

[8] Li, X.; Shen, Y.; Zhang, G.; Zheng, X.; Zhao, Q.; Song, Z. *Org. Lett.* **2022,** *24*, 5281–5286.

Determination of Kinetic Isotope Effects

(a) Reversibility/scrambling tests.

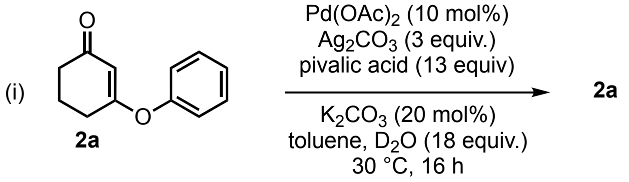

- No appreciable deuterium incorporation was observed for ***2a****.*

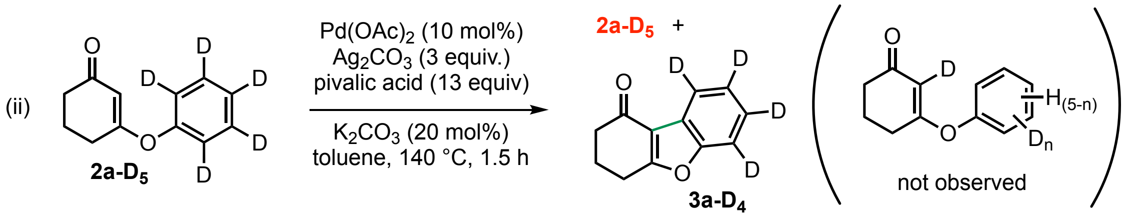

- No appreciable deuterium exchange was observed for ***2a-D₅****.*

(b) KIE determined from two parallel reactions.

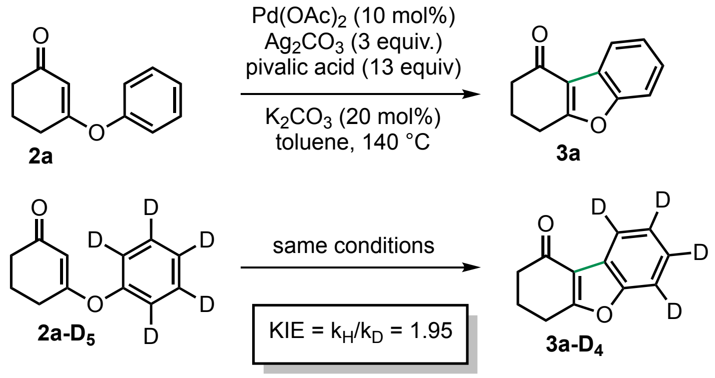

A sealed tube containing 3-phenoxycyclohex-2-en-1-one (**2a**) (0.5 mmol, 94 mg), silver carbonate (1.5 mmol, 414 mg), potassium carbonate (0.1 mmol, 14 mg), pivalic acid (6.5 mmol, 664 mg) and palladium acetate (0.05 mmol, 11 mg) in toluene (5 mL) was stirred at 140 °C. In a parallel experiment, a reaction was performed using 3-(phenoxy-d5)cyclohex-2-en-1-one (**2a-D$_5$**) (0.5 mmol, 96 mg) as a substrate under otherwise identical conditions. Aliquots were removed from both reactions at 20, 40, 60, and 90 minutes. The samples were diluted with DCM and filtered through a pad of elite. The filtrates were dried over anhydrous $MgSO_4$ and concentrated under reduced pressure. The crude reaction mixtures were analyzed by ^{1}H NMR, and the conversion (%) to the corresponding products 3,4-dihydrodibenzo[b,d]furan-1(2H)-one (**3a**) or 3,4-

dihydrodibenzo[b,d]furan-1(2H)-one-6,7,8,9-d4 (**3a-D$_4$**) of each sample was plotted against the reaction times.

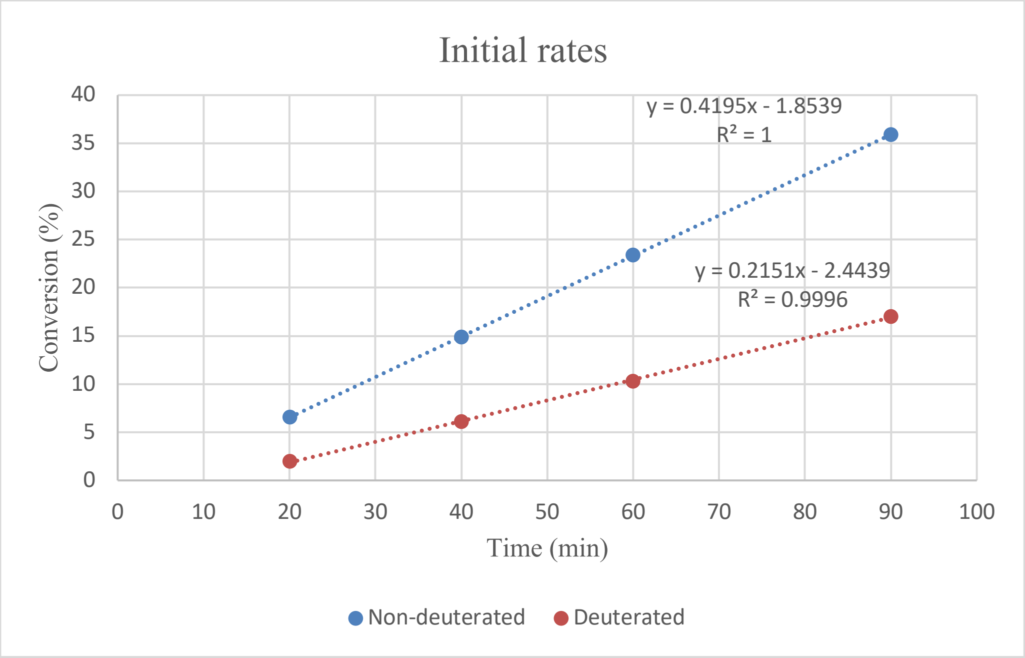

KIE = 0.4195/0.2151 = 1.95 (data shown is the average of two runs)

(c) KIE determined from an intermolecular competition.

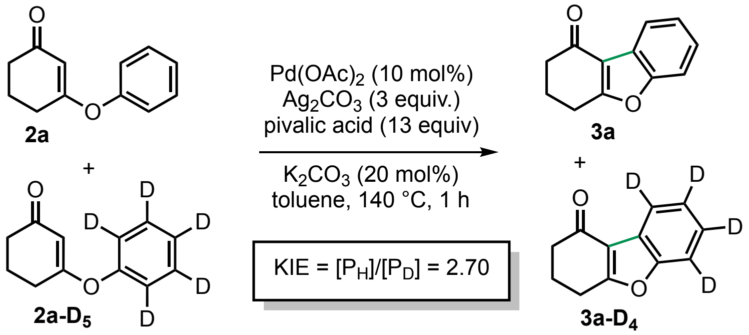

A sealed tube was charged with **2a** (43 mg, 0.25 mmol), **2a-D$_5$** (45 mg, 0.25 mmol), $Pd(OAc)_2$ (12 mg, 0.05 mmol), pivalic acid (664 mg, 6.5 mmol), $AgCO_3$ (414 mg, 1.5 mmol) and K_2CO_3 (13.8 mg, 0.1 mmole). The mixture was stirred at 140 °C. After 60 minutes, an aliquot was removed from the reaction. The sample was diluted with DCM and filtered through a pad of celite. The filtrates were concentrated under reduced pressure and purified by flash column chromatography. The reaction mixture was analyzed by 1H NMR to determine the ratio of **3a** and **3a-D$_4$**.

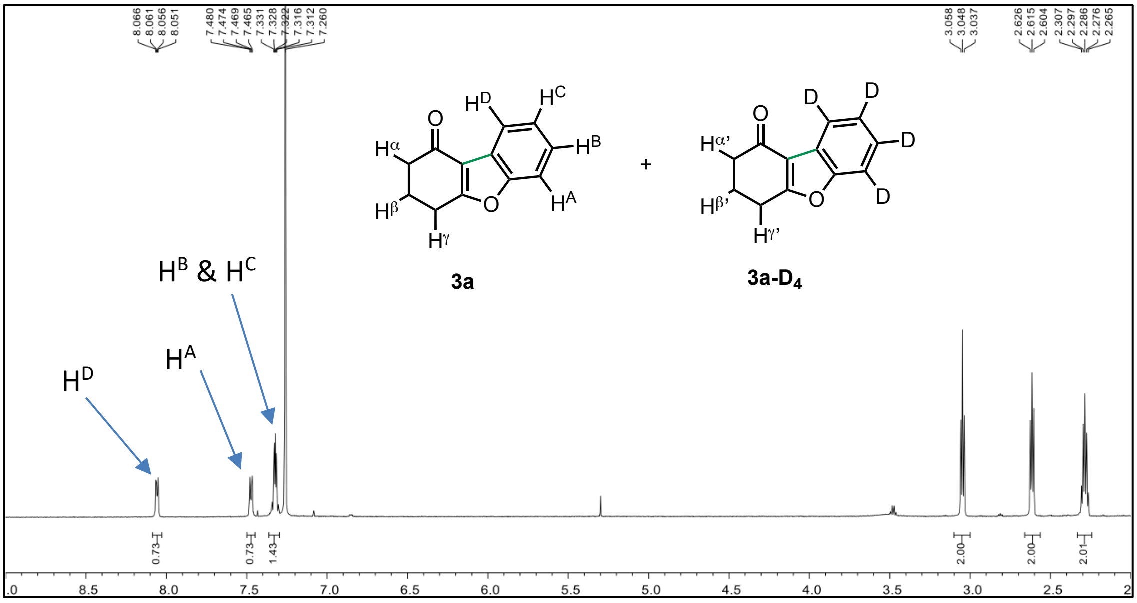

KIE = $[P_H]/[P_D] = (H^B + H^C)/[(H^{\alpha} + H^{\alpha'}) – (H^B + H^C) = 0.73/(1-0.73) = 2.70$

^{1}H and ^{13}C NMR Spectra

^{1}H NMR (400 MHz, $CDCl_3$) spectrum of compound 1a

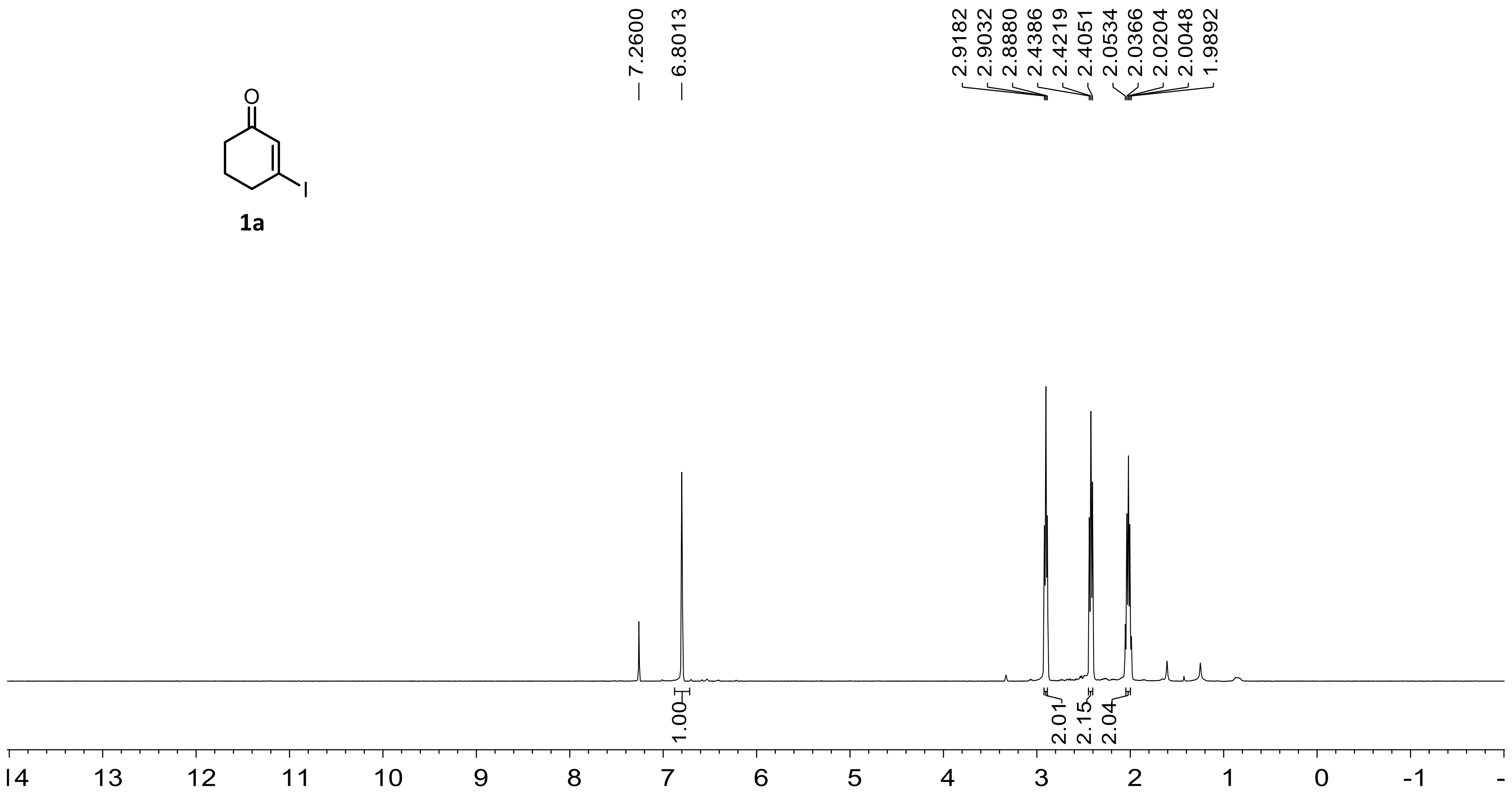

^{1}H NMR (400 MHz, CD_3OD) spectrum of compound 1b

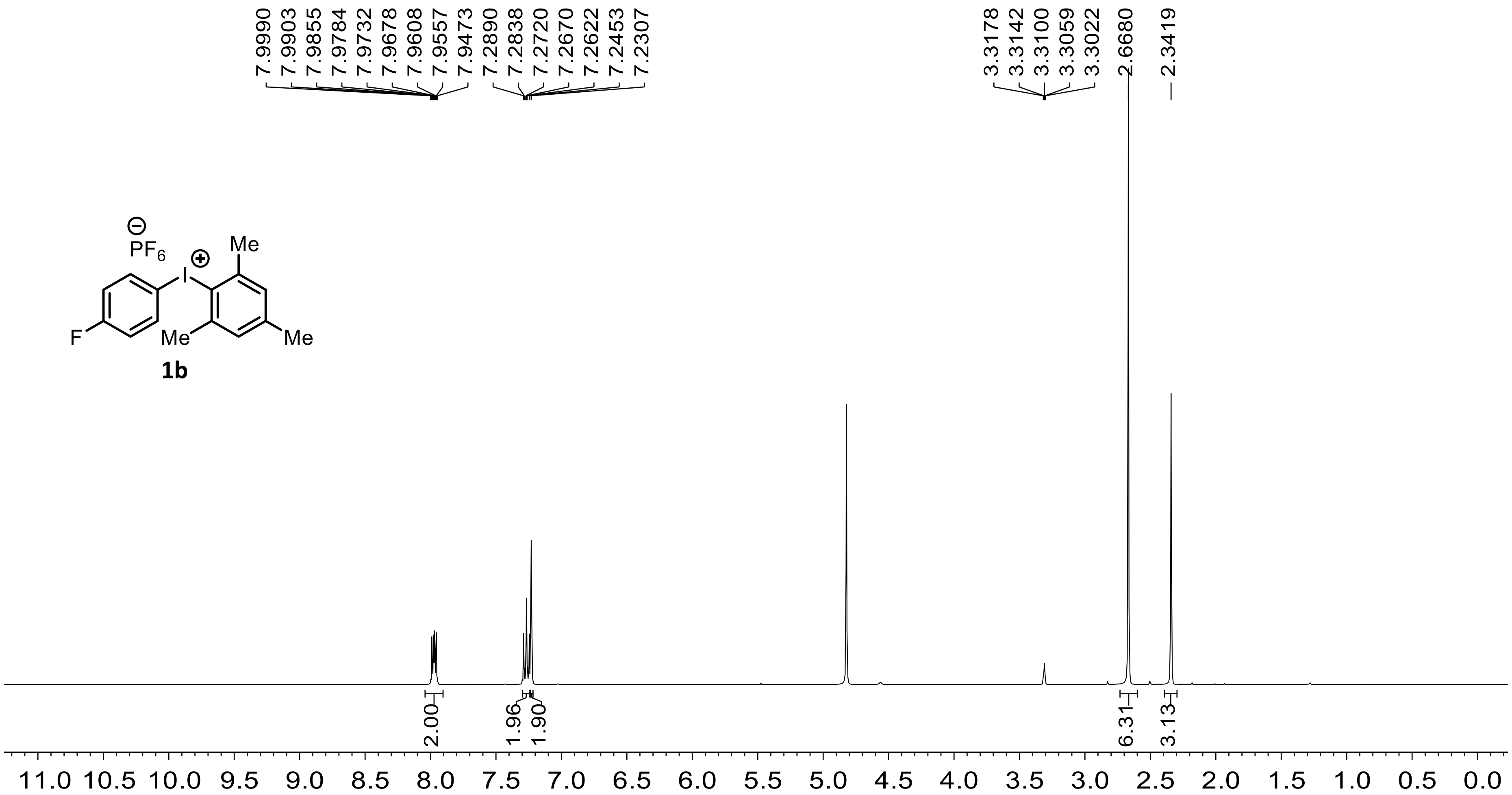

^1H NMR (400 MHz, CD_3OD) spectrum of compound 1c

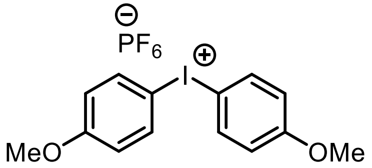

1c

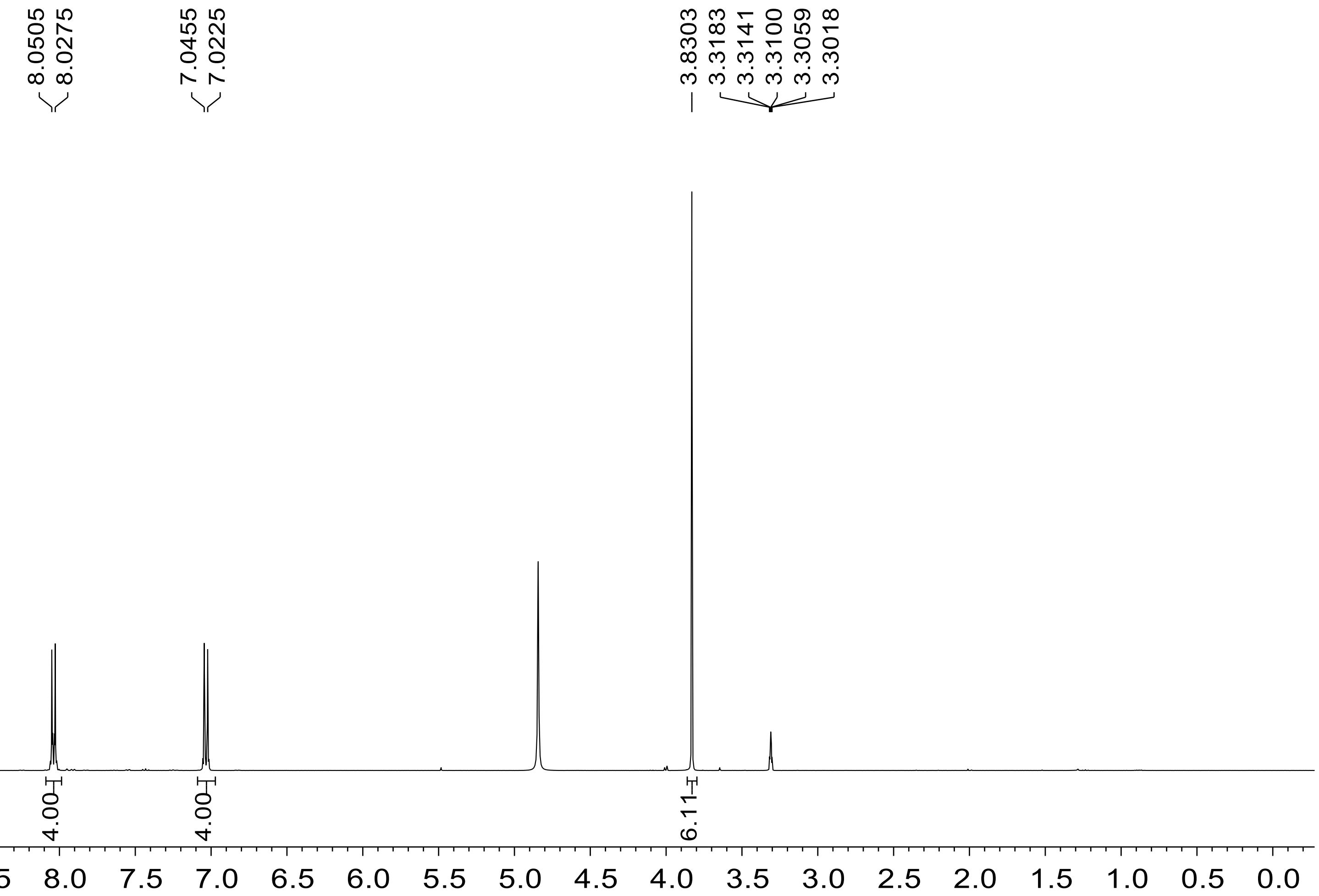

^{1}H NMR (400 MHz, CD_3OD) spectrum of compound 1d

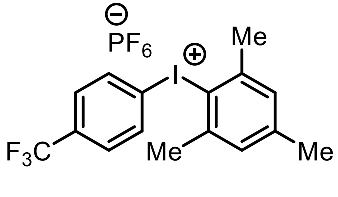

1d

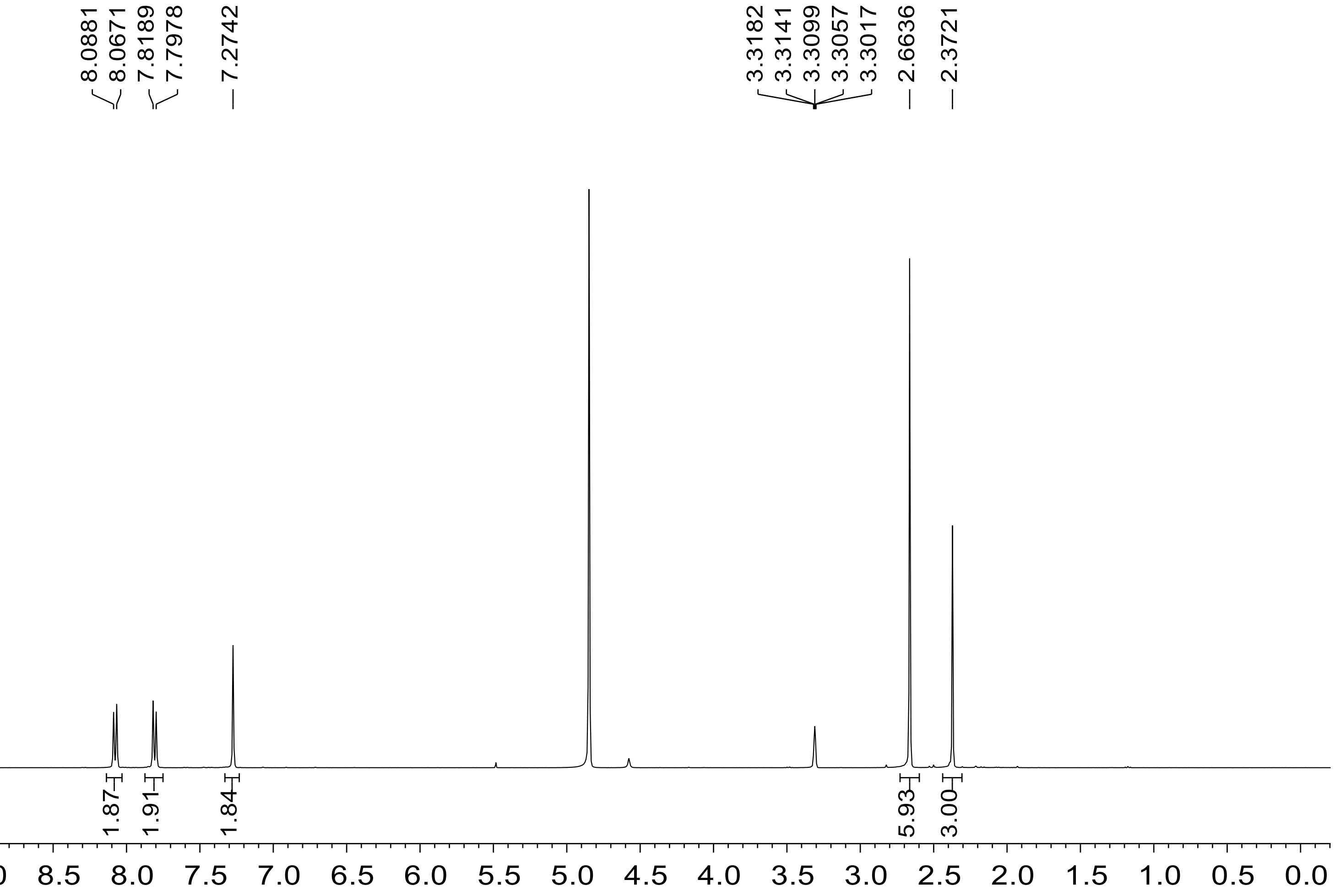

^{1}H NMR (400 MHz, CD_3OD) spectrum of compound 1e

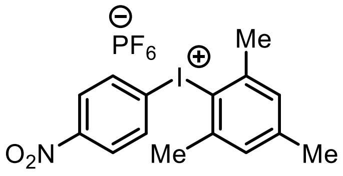

1e

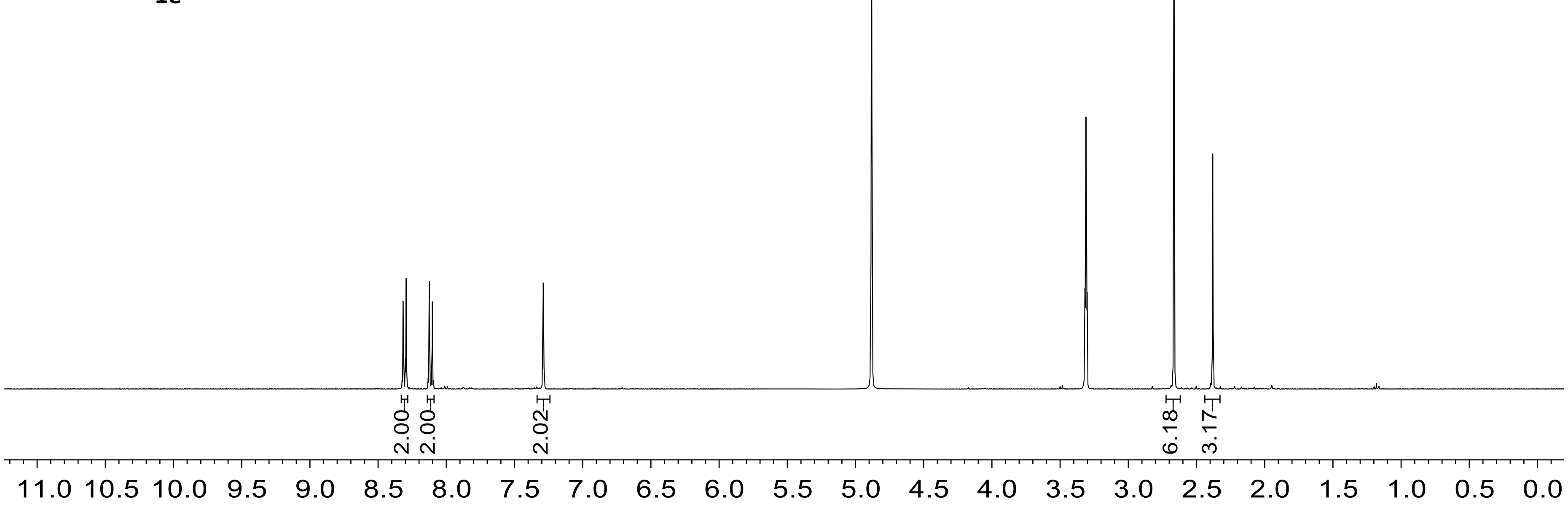

$^{13}C\{^{1}H\}$ NMR (100 MHz, CD_3OD) spectrum of compound 1e

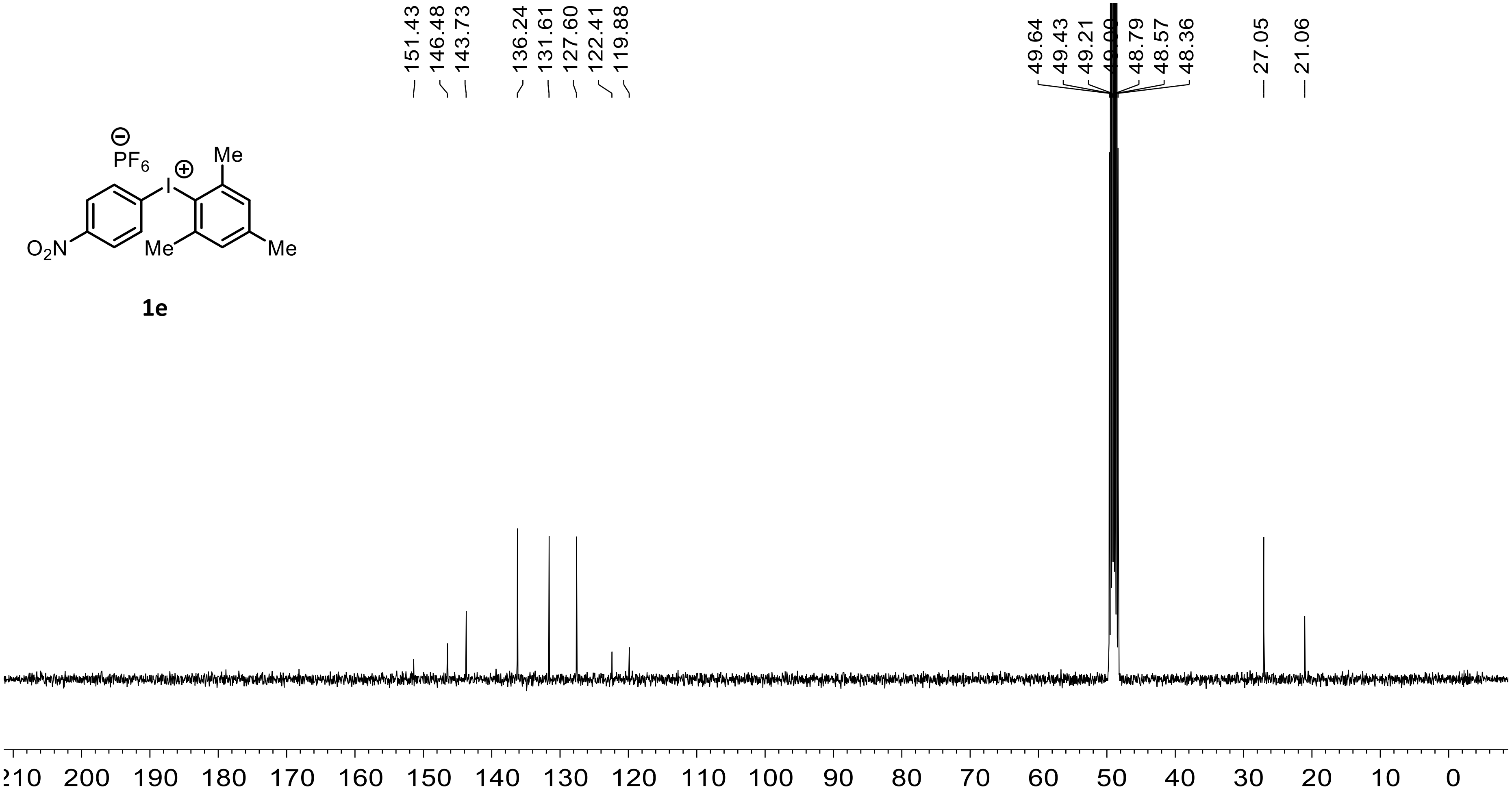

^{1}H NMR (400 MHz, CD_3OD) spectrum of compound 1f

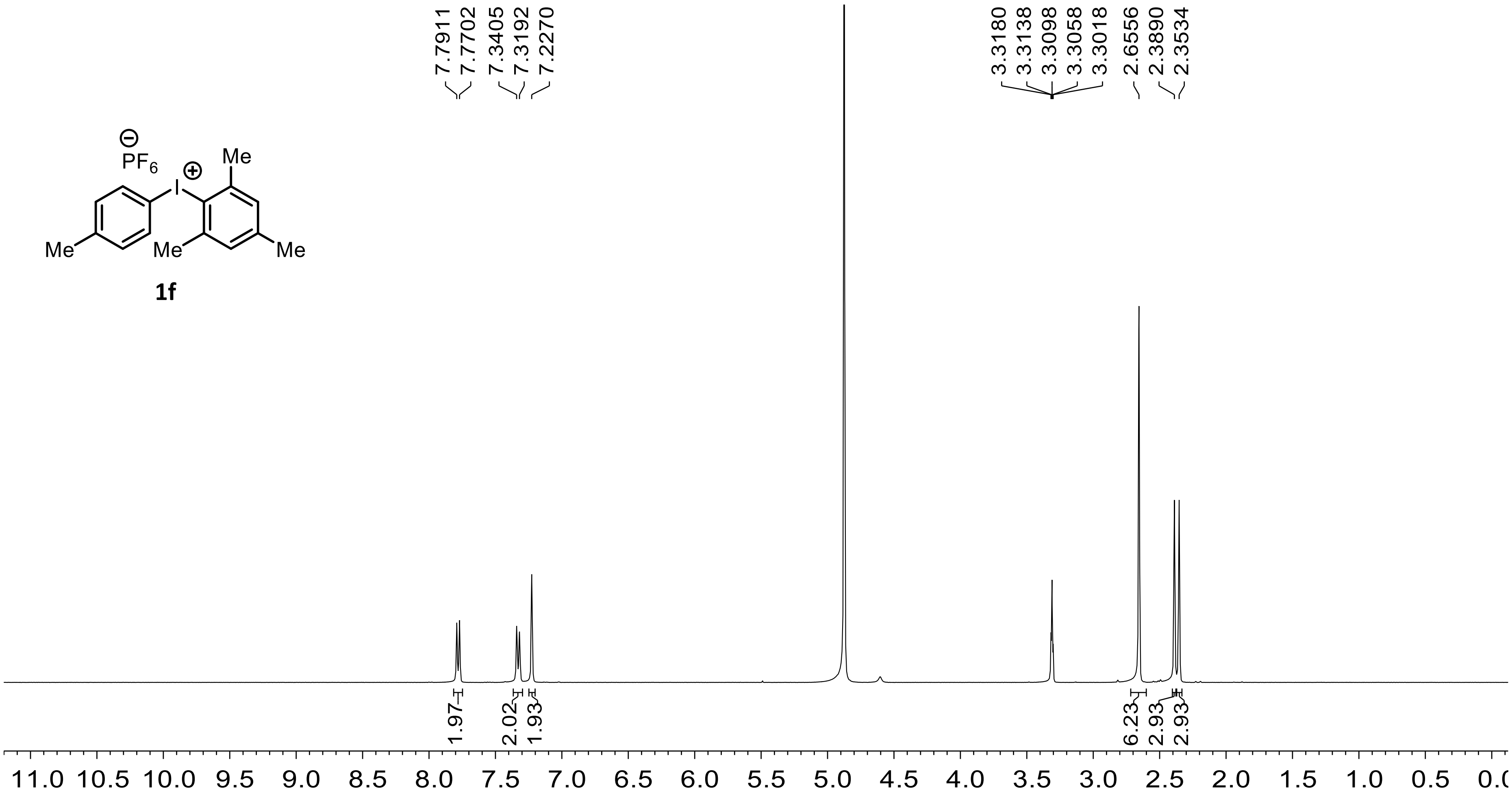

^1H NMR (400 MHz, $CDCl_3$) spectrum of compound 2a

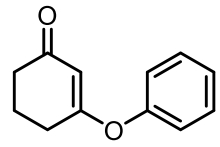

2a

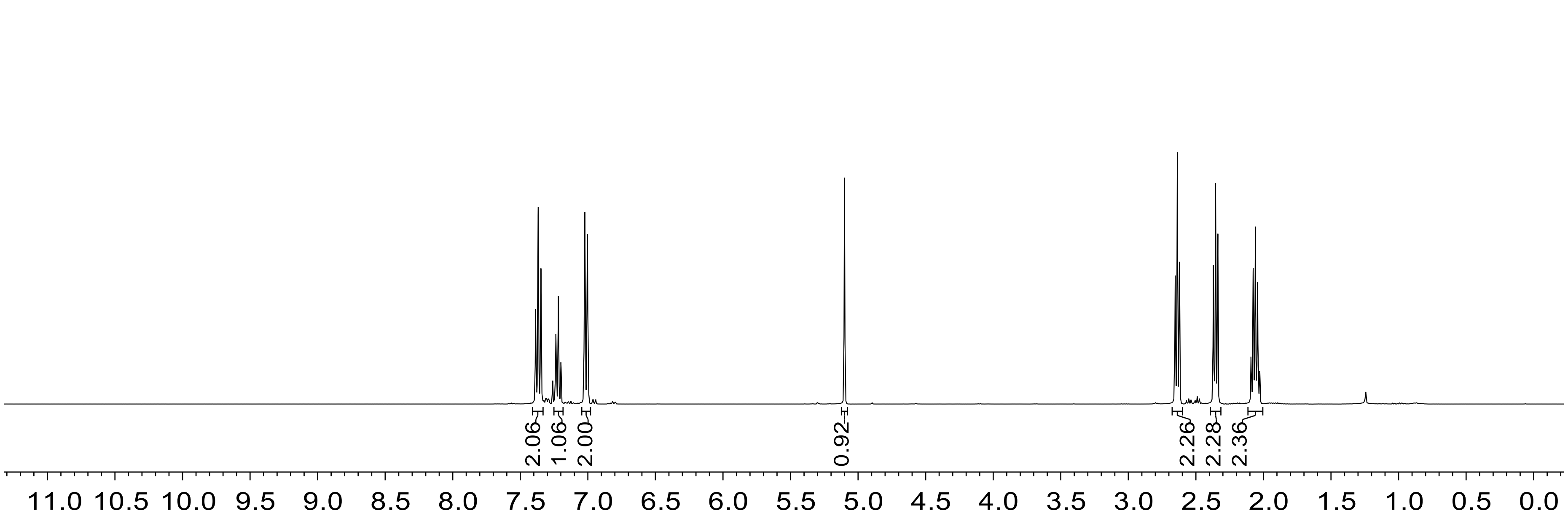

^{1}H NMR (400 MHz, $CDCl_3$) spectrum of compound 2b

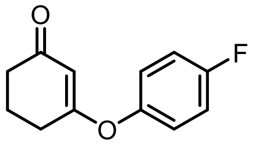

2b

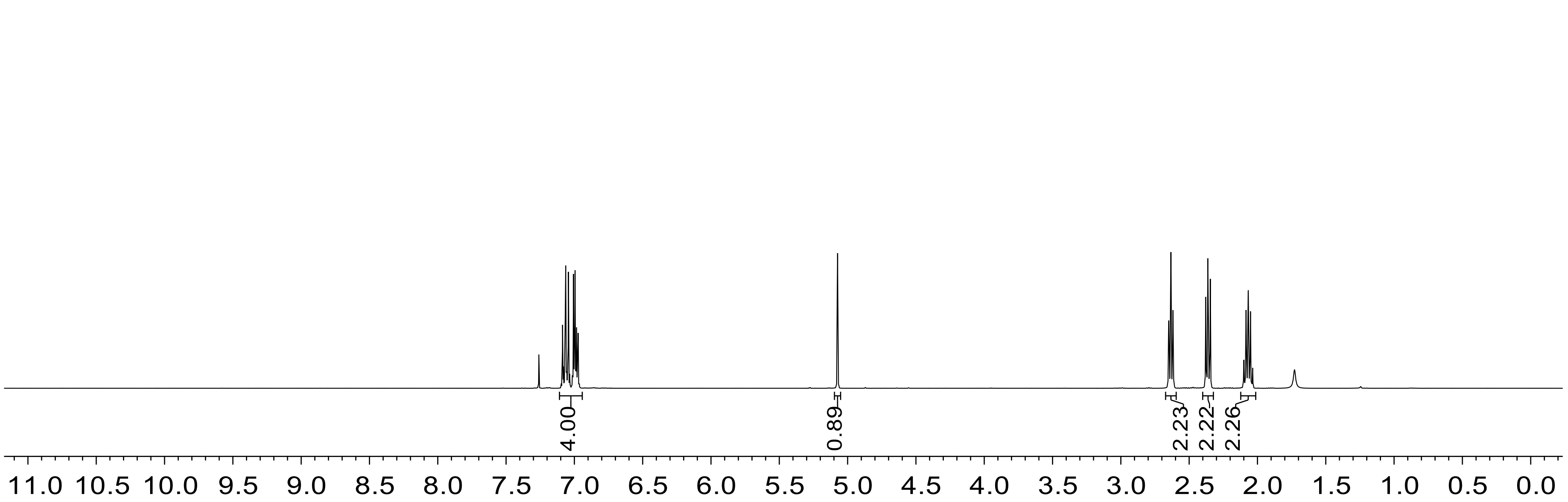

$^{13}C\{^{1}H\}$ NMR (100 MHz, $CDCl_3$) spectrum of compound 2b

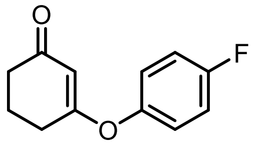

2b

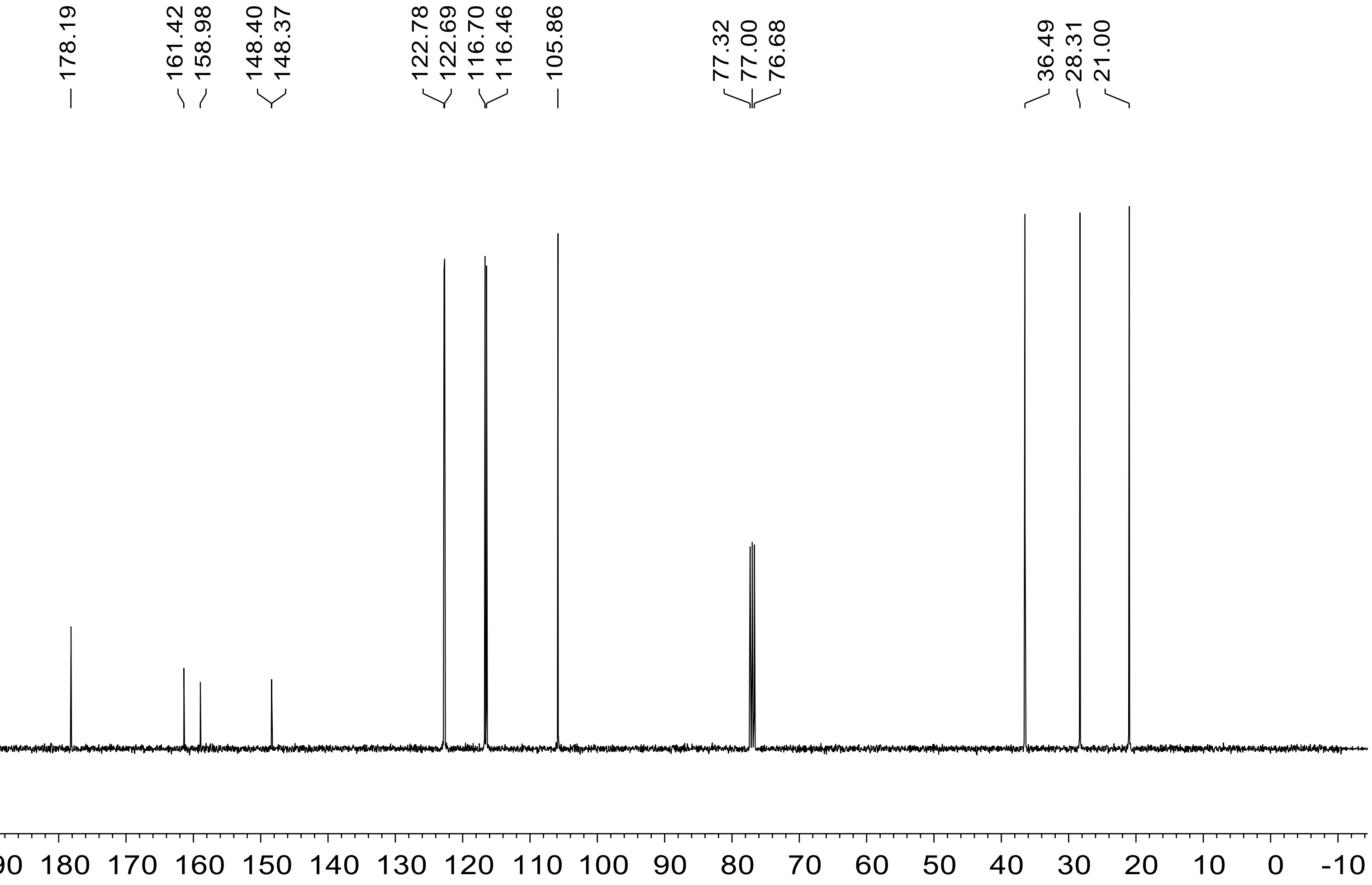

^1H NMR (400 MHz, $CDCl_3$) spectrum of compound 2c

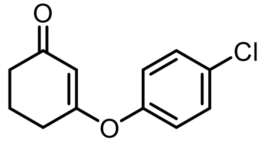

2c

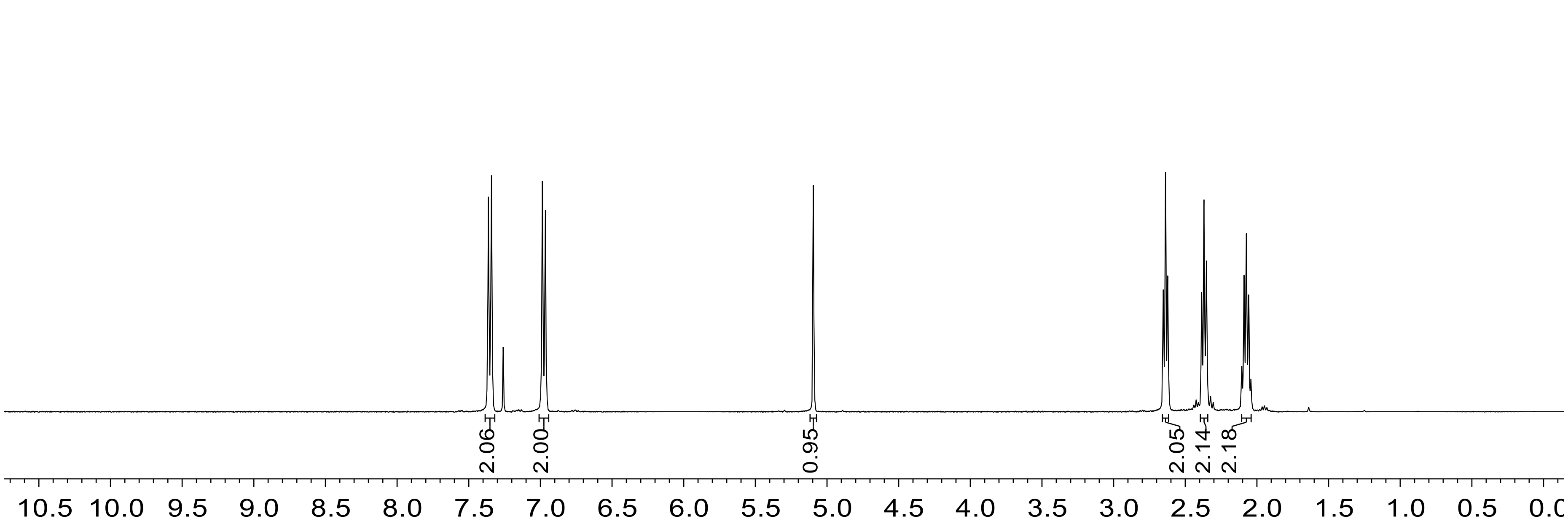

$^{13}C\{^{1}H\}$ NMR (100 MHz, $CDCl_3$) spectrum of compound 2c

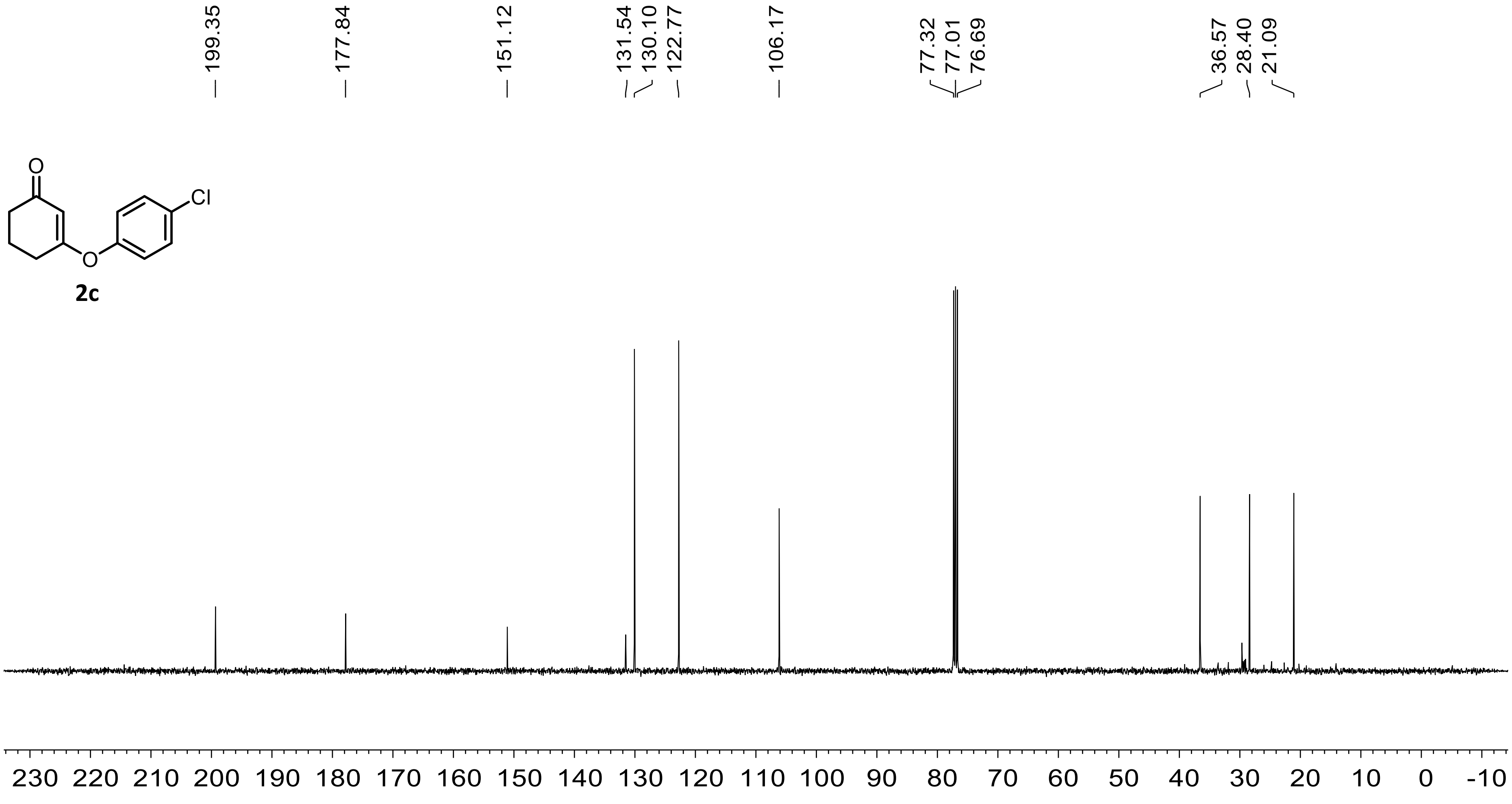

^1H NMR (400 MHz, $CDCl_3$) spectrum of compound 2d

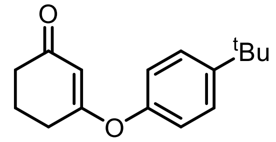

2d

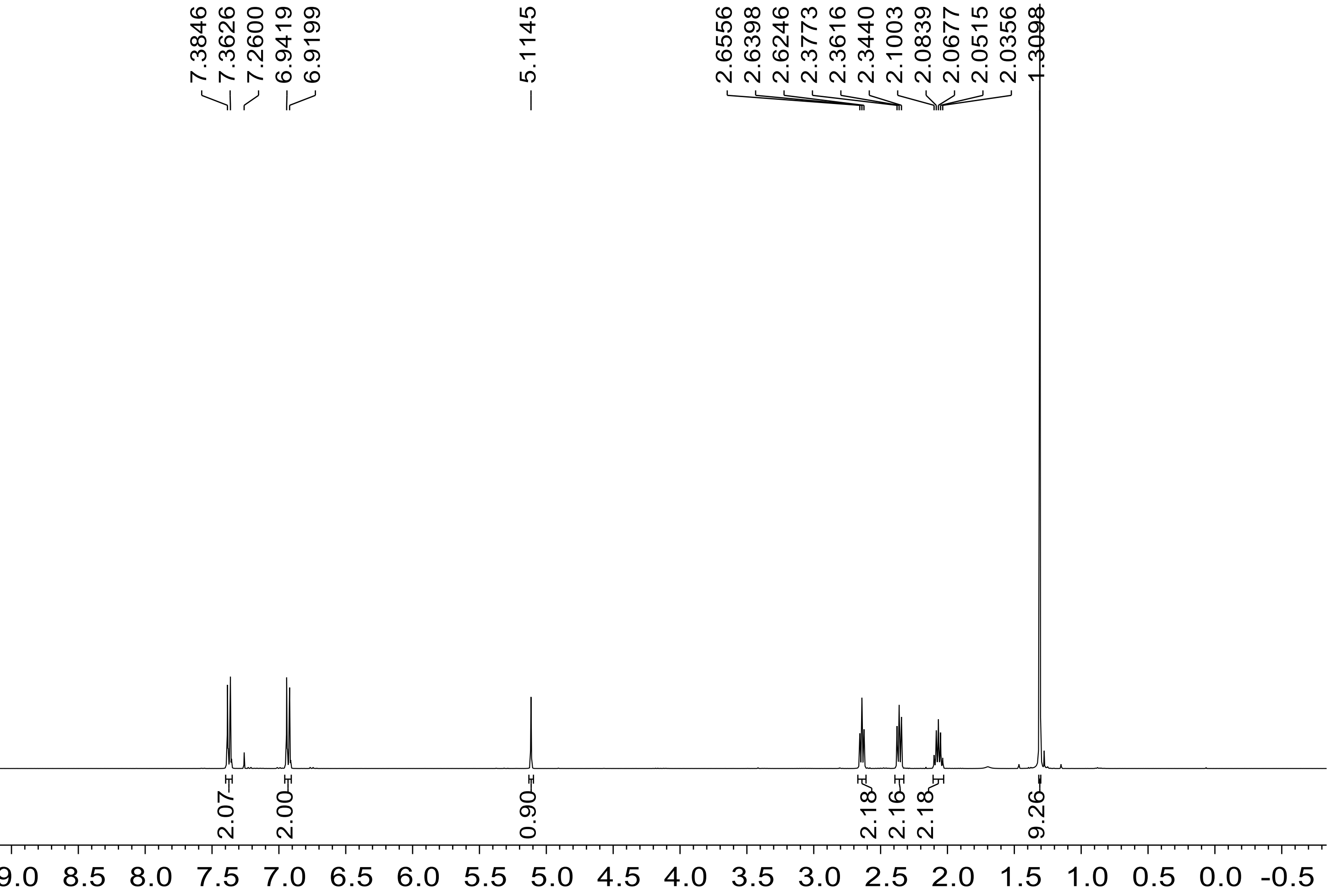

$^{13}C\{^1H\}$ NMR (100 MHz, $CDCl_3$) spectrum of compound 2d

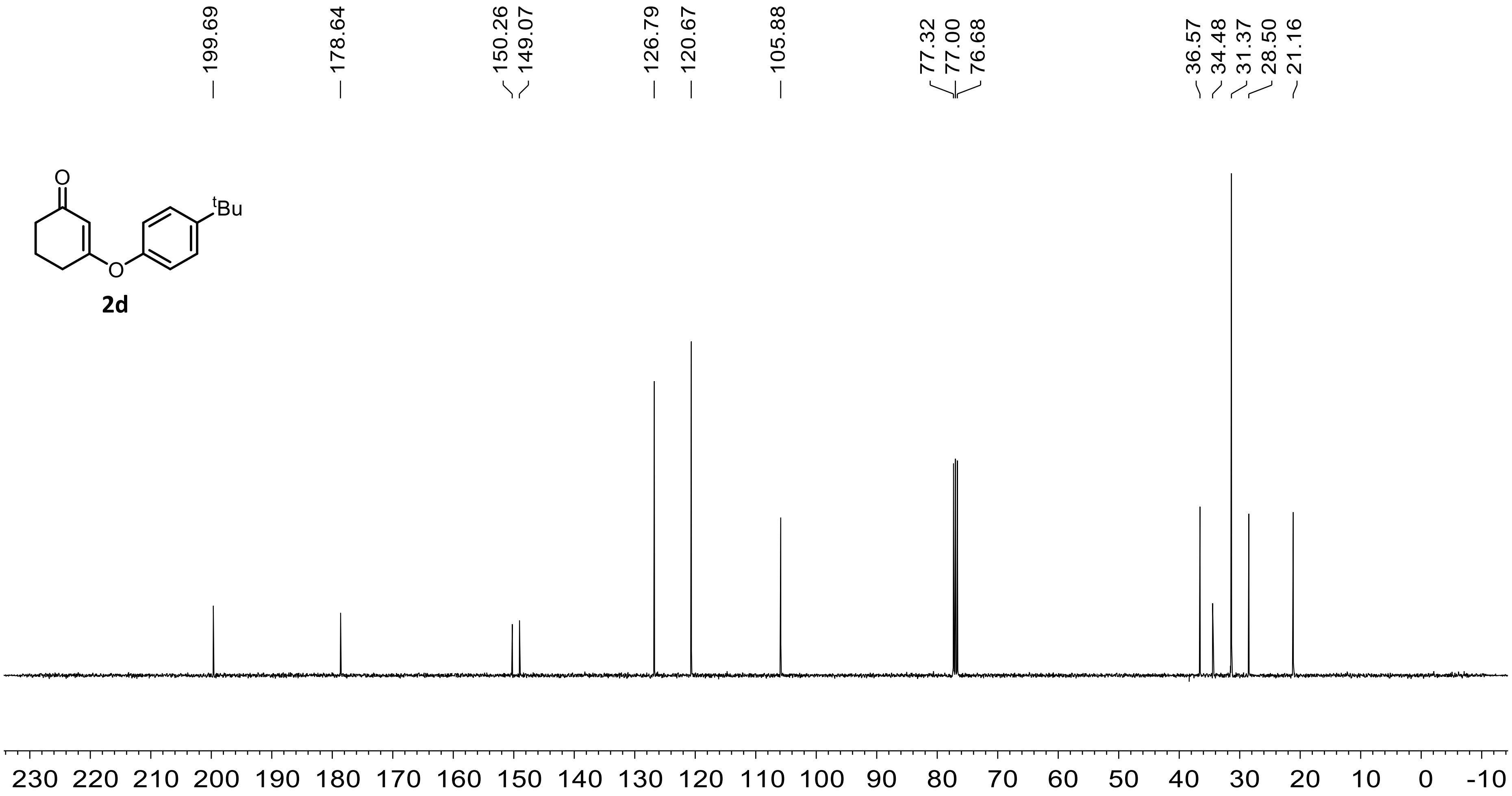

^{1}H NMR (400 MHz, $CDCl_3$) spectrum of compound 2e

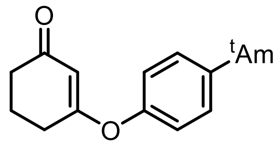

2e

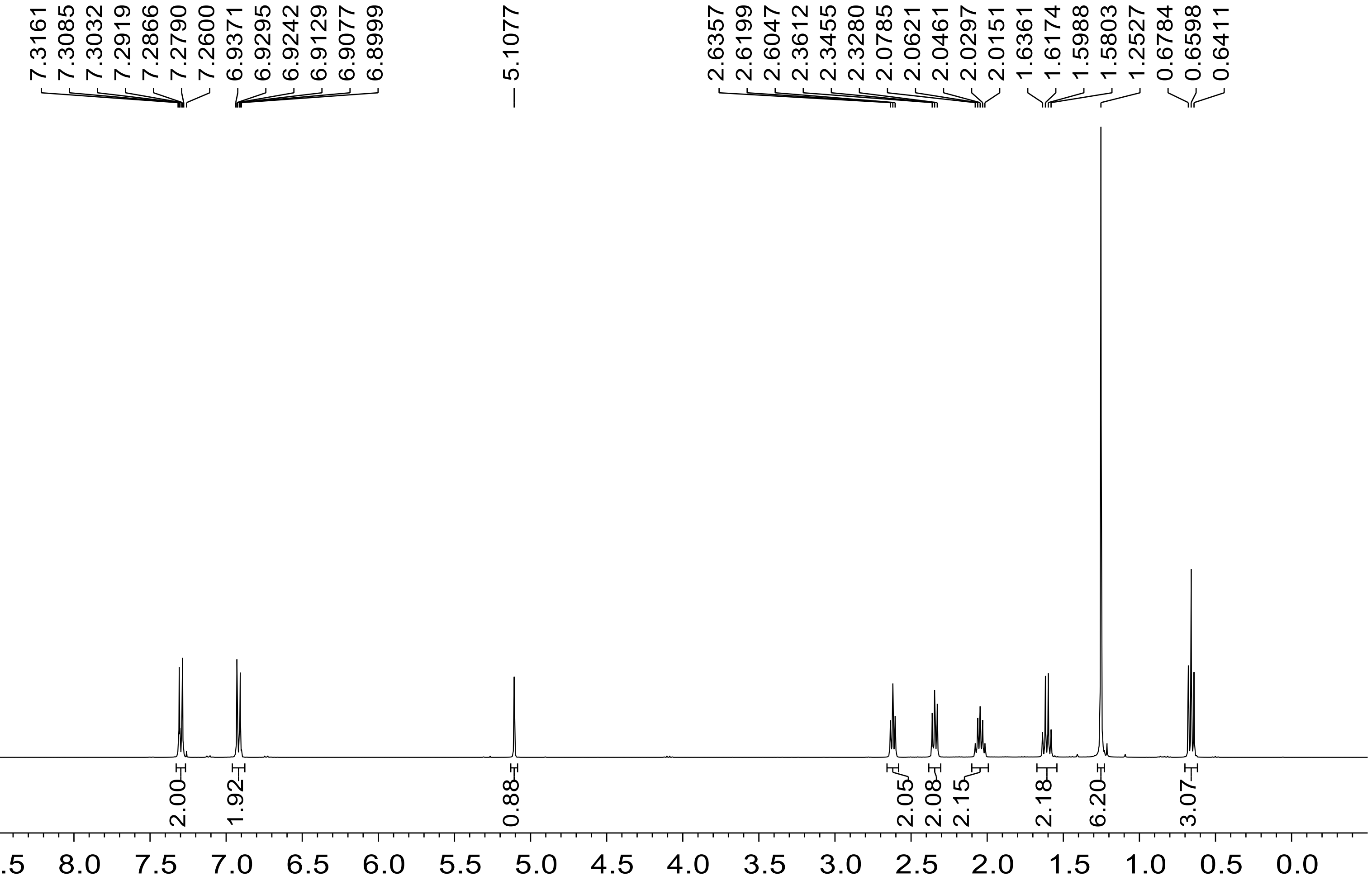

$^{13}C\{^{1}H\}$ NMR (100 MHz, $CDCl_3$) spectrum of compound 2e

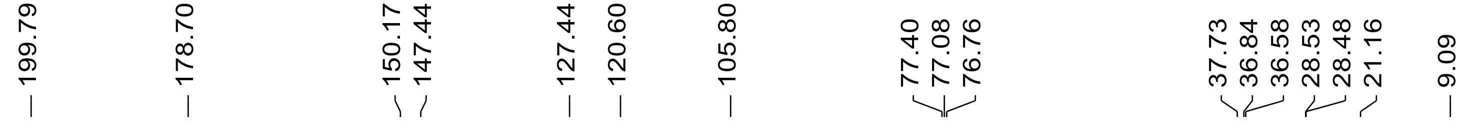

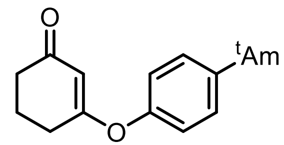

2e

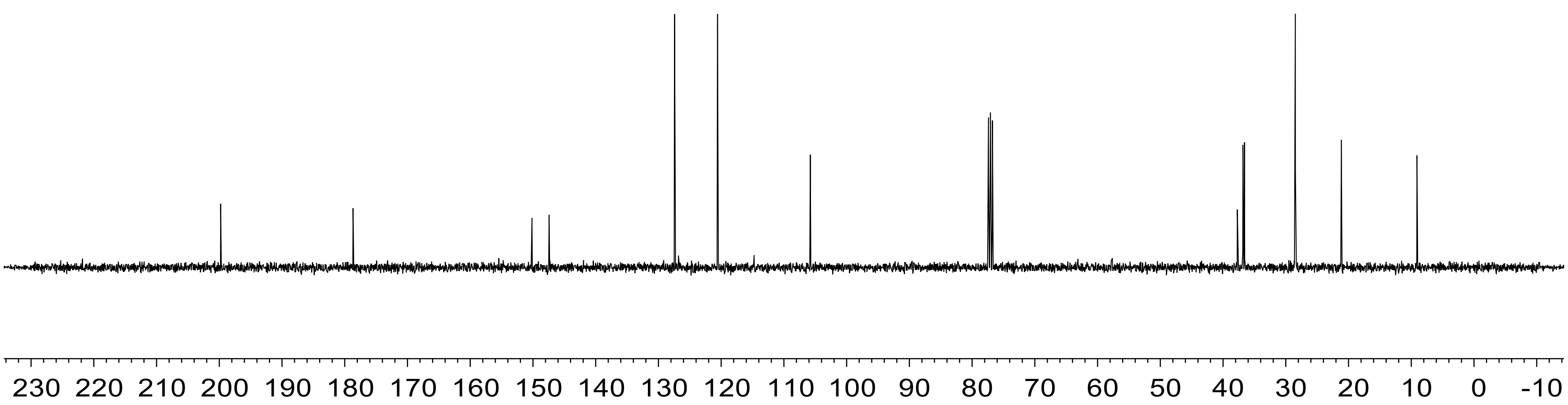

^{1}H NMR (400 MHz, $CDCl_3$) spectrum of compound 2f

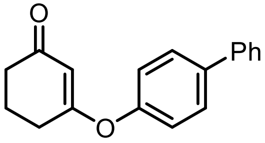

2f

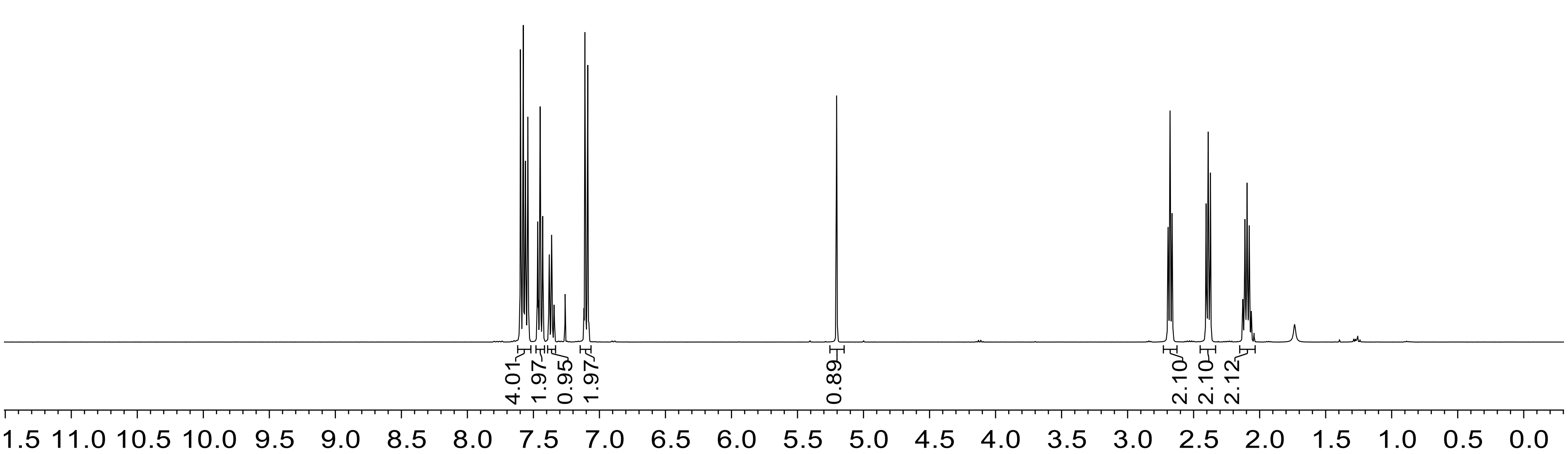

$^{13}C\{^1H\}$ NMR (100 MHz, $CDCl_3$) spectrum of compound 2f

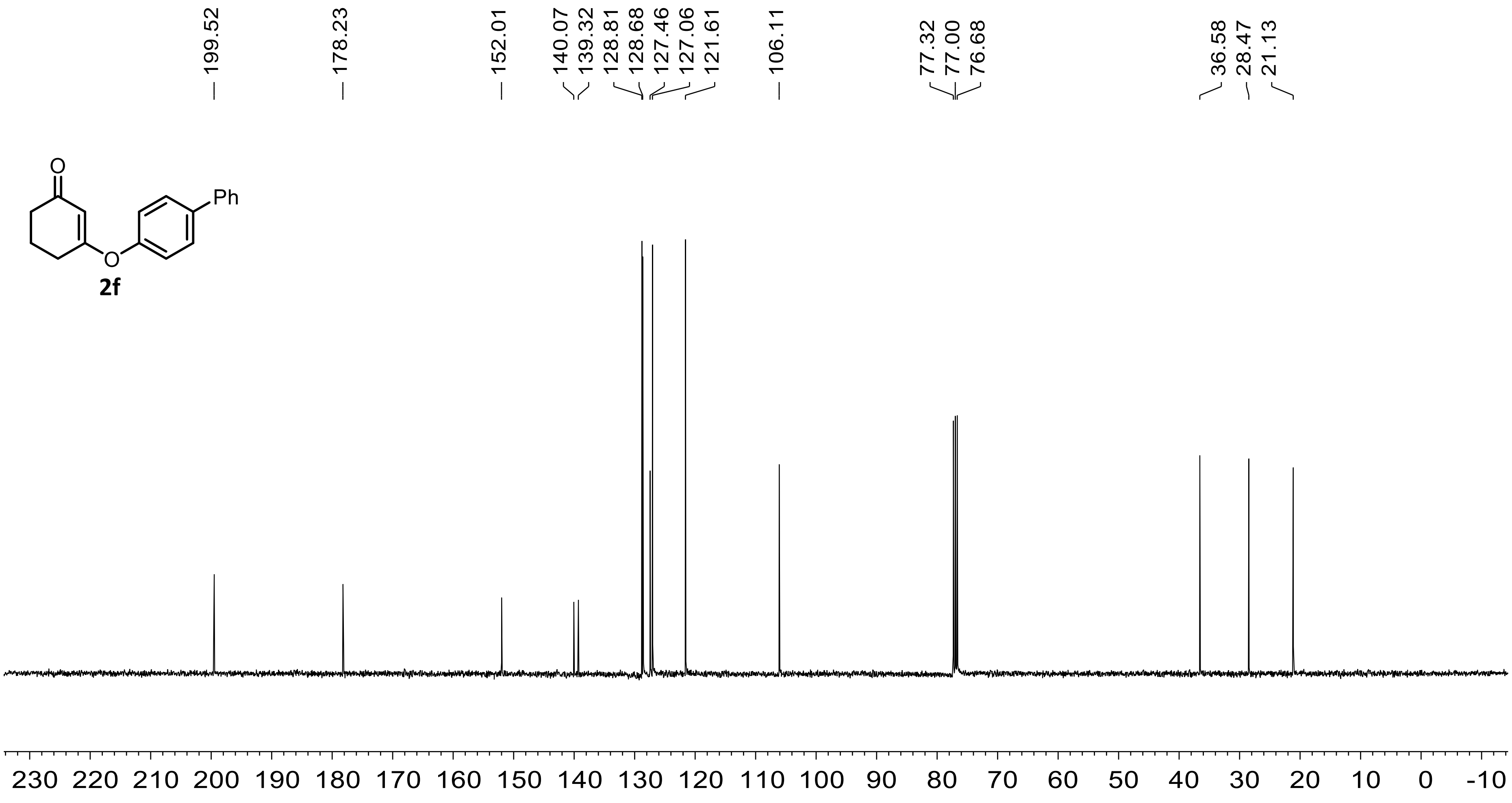

^{1}H NMR (400 MHz, $CDCl_3$) spectrum of compound 2g

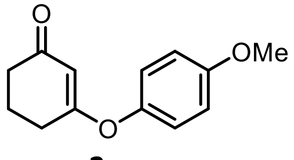

2g

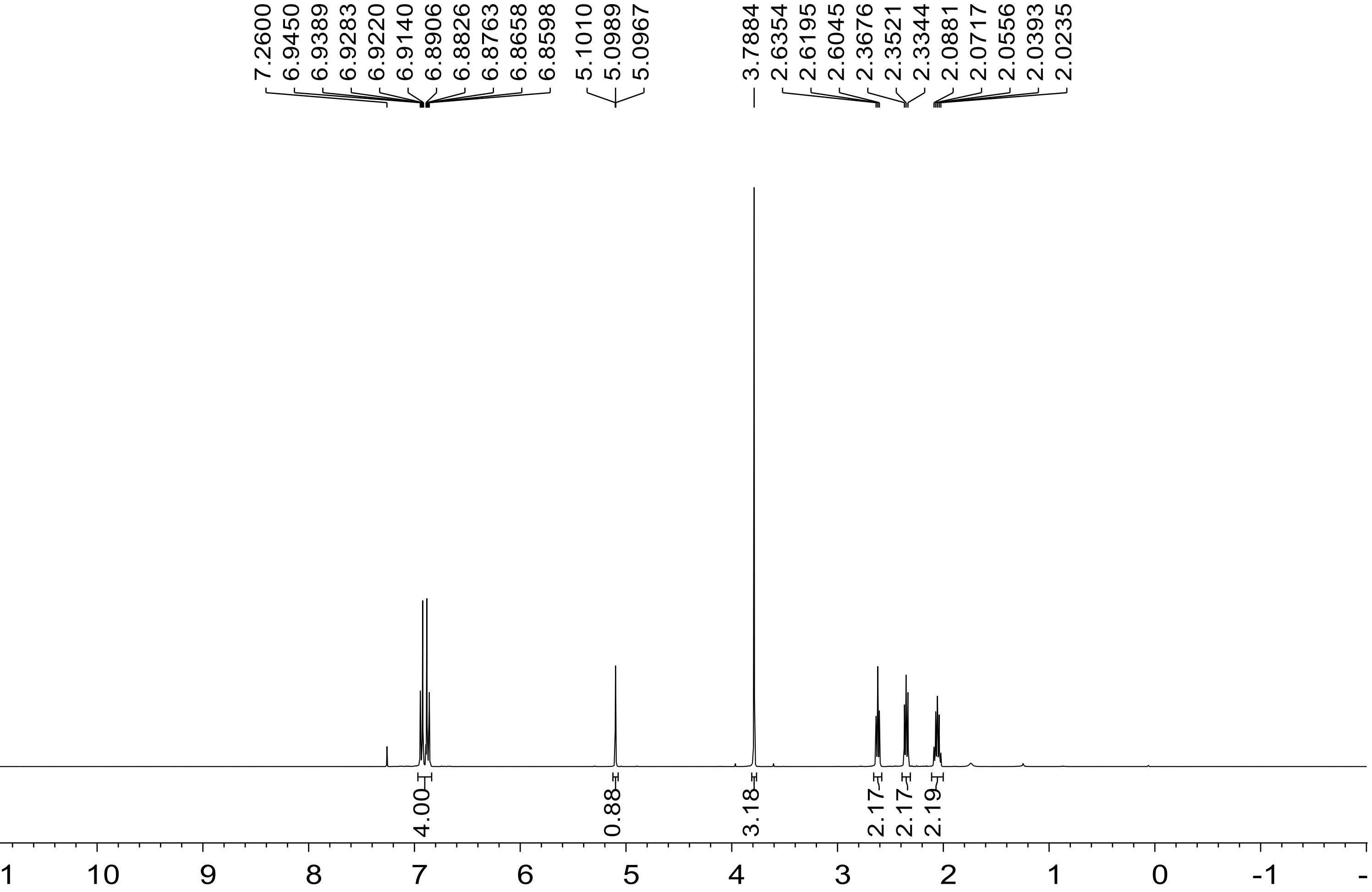

$^{13}C\{^1H\}$ NMR (100 MHz, $CDCl_3$) spectrum of compound 2g

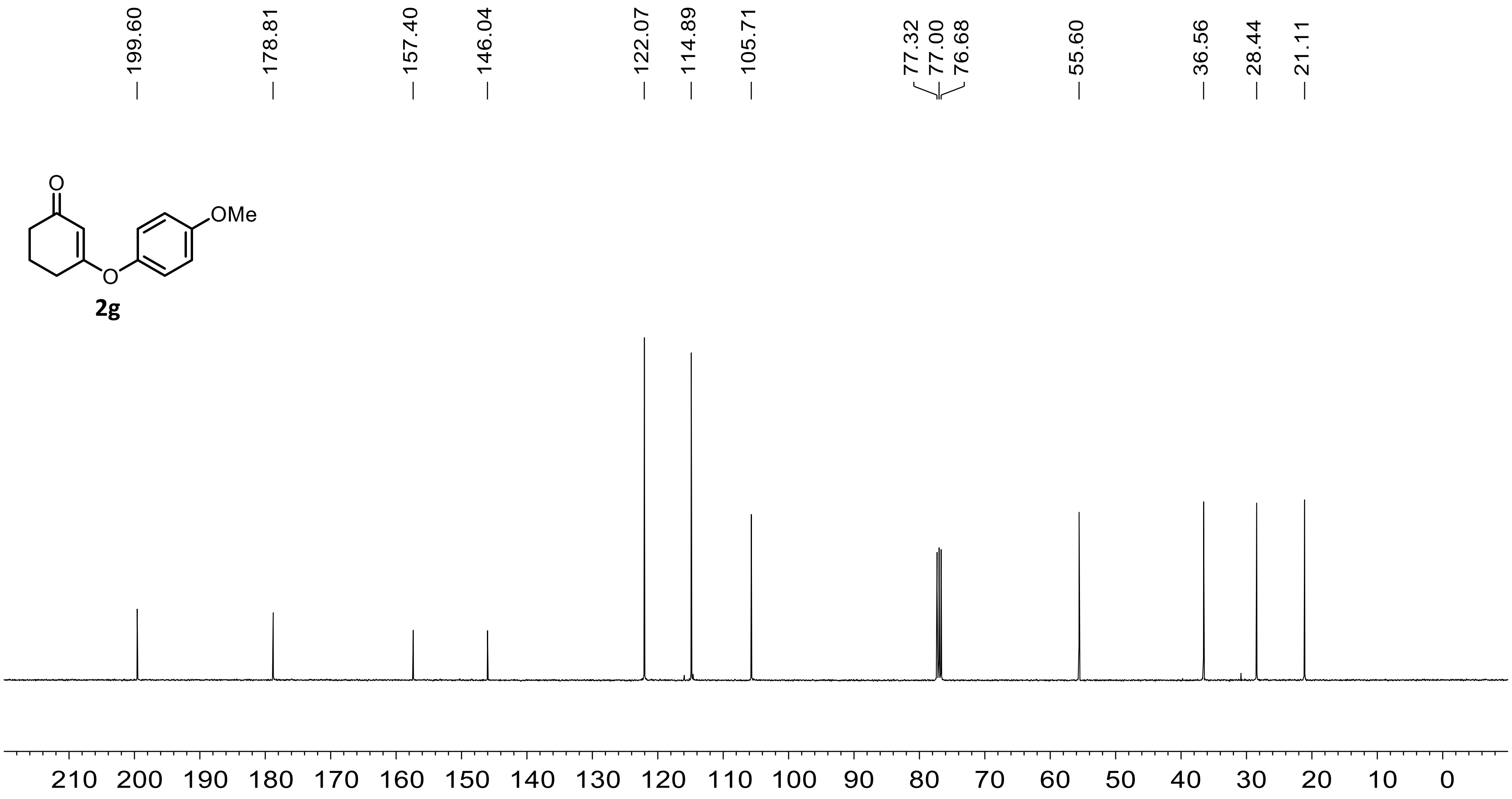

^{1}H NMR (400 MHz, $CDCl_3$) spectrum of compound 2h

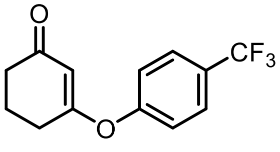

2h

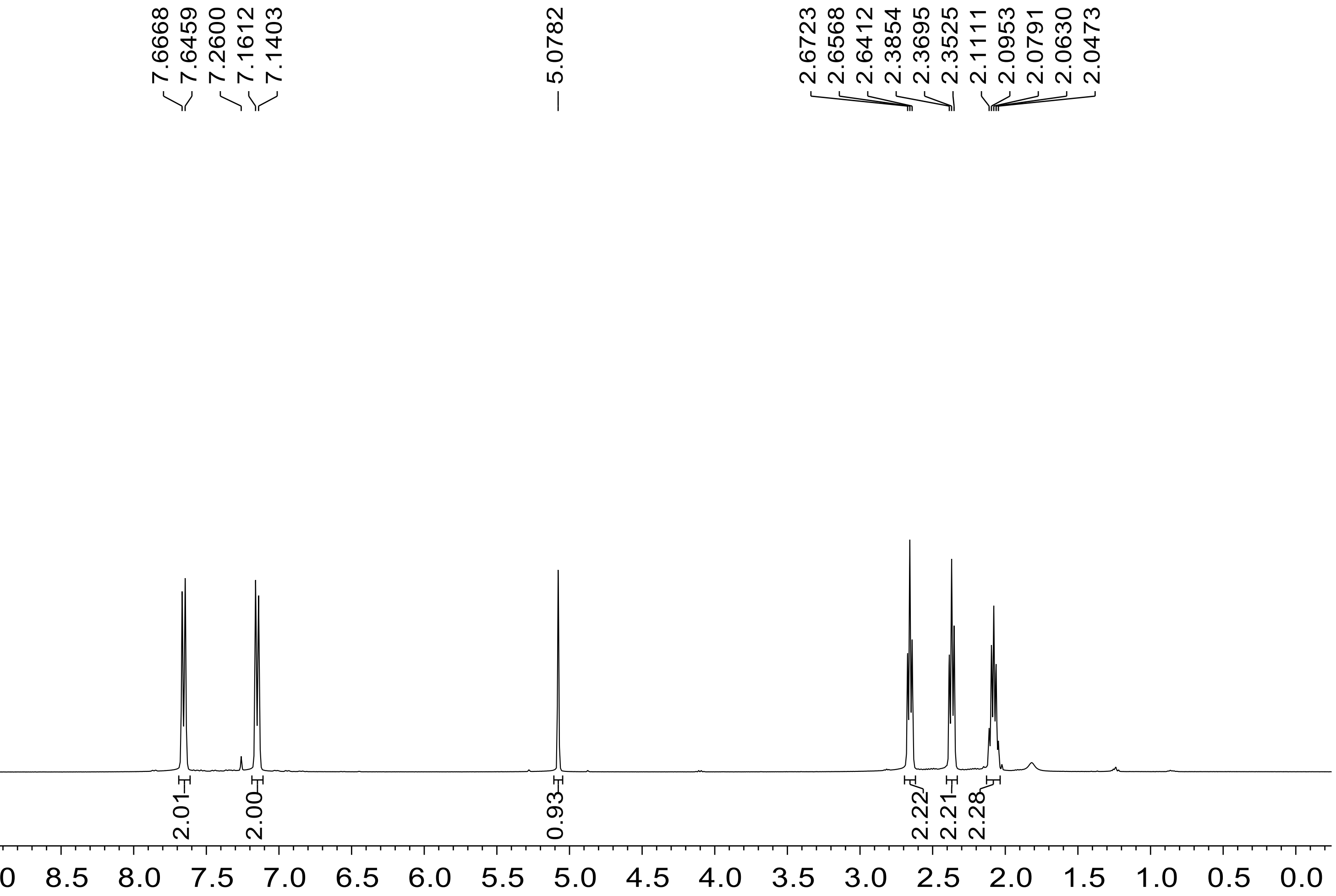

$^{13}C\{^1H\}$ NMR (100 MHz, $CDCl_3$) spectrum of compound 2h

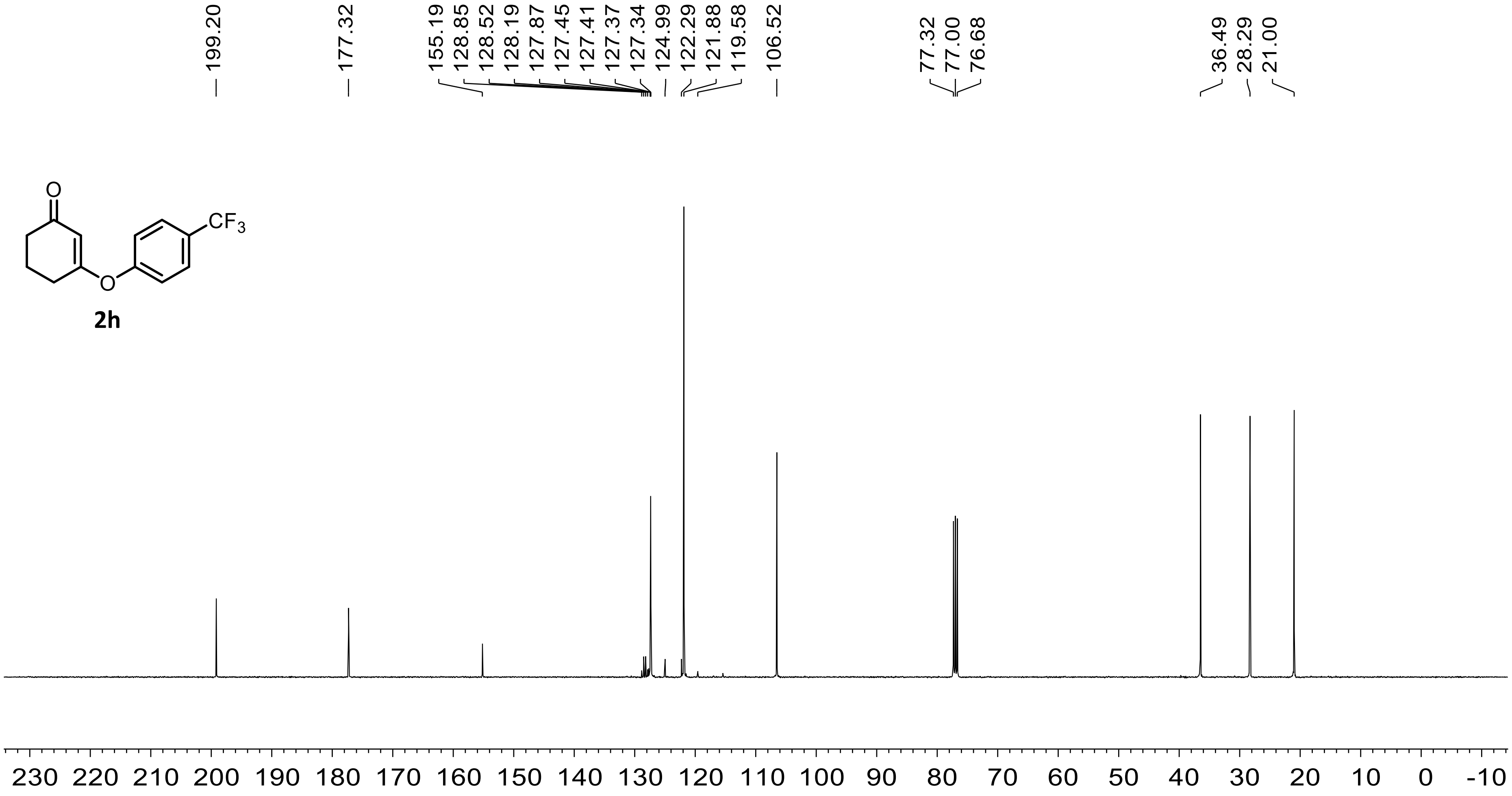

^1H NMR (400 MHz, $CDCl_3$) spectrum of compound 2i

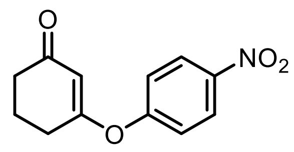

2i

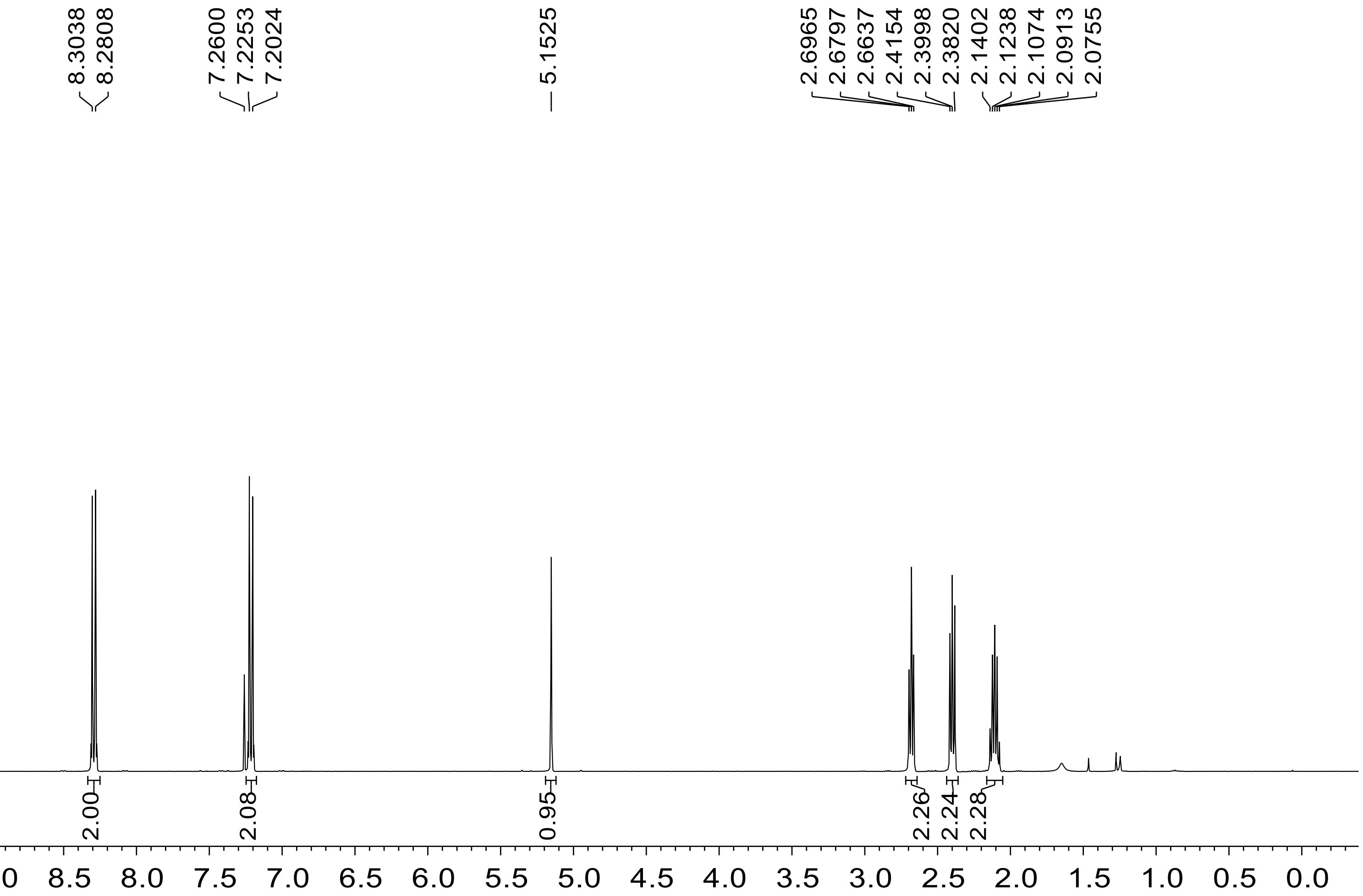

$^{13}C\{^1H\}$ NMR (100 MHz, $CDCl_3$) spectrum of compound 2i

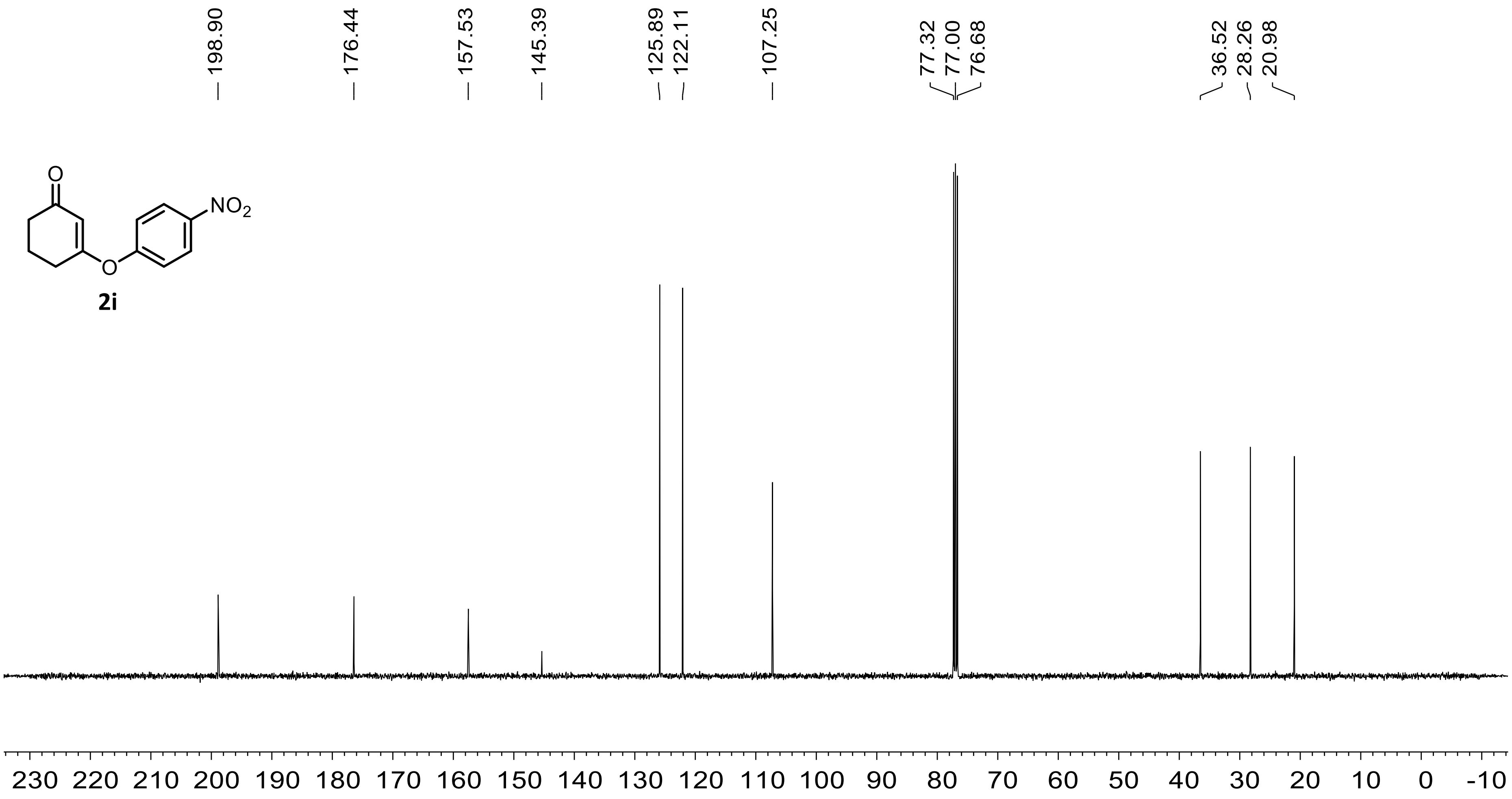

2i

^{1}H NMR (400 MHz, $CDCl_3$) spectrum of compound 2j

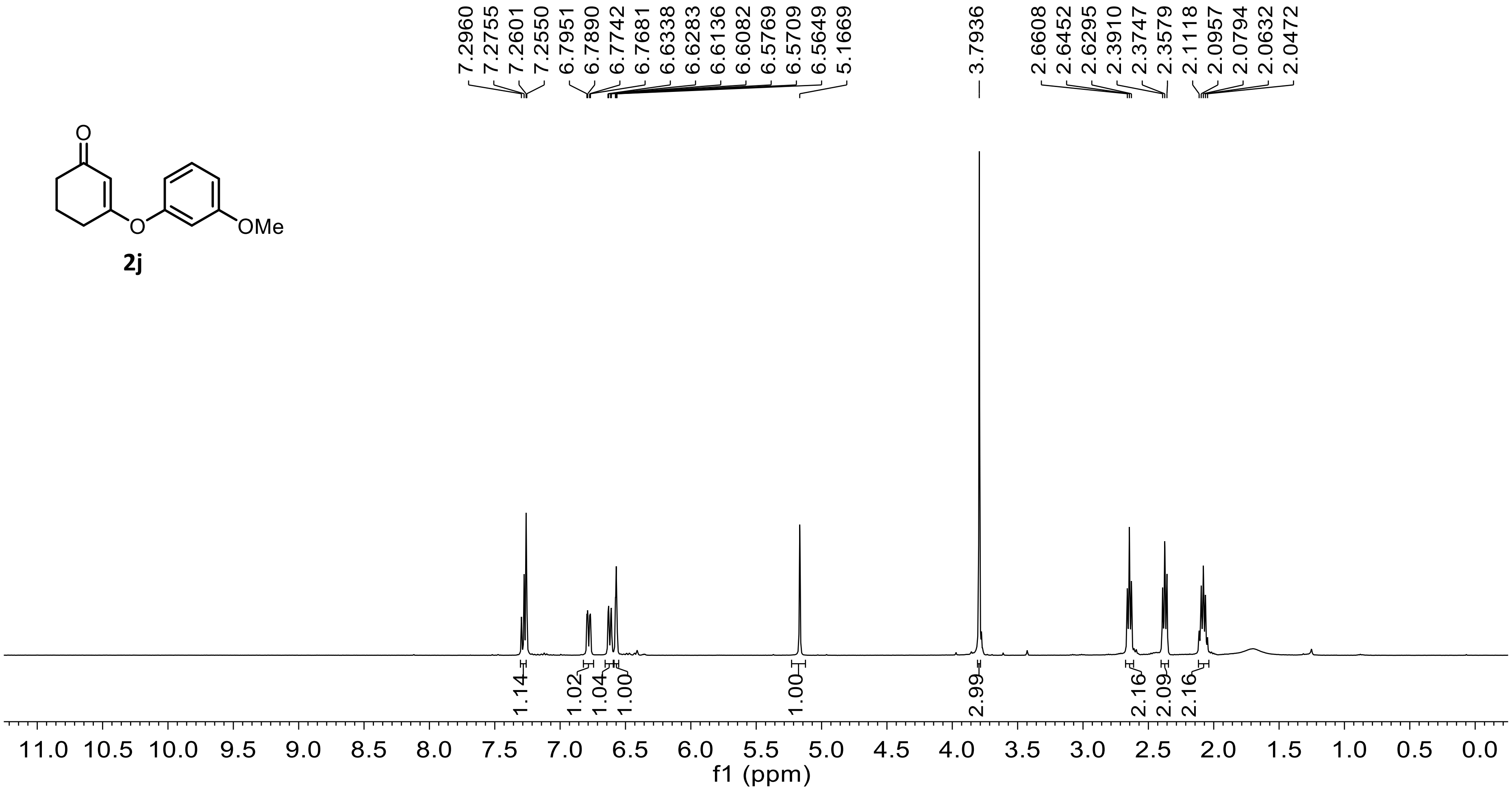

$^{13}C\{^1H\}$ NMR (100 MHz, $CDCl_3$) spectrum of compound 2j

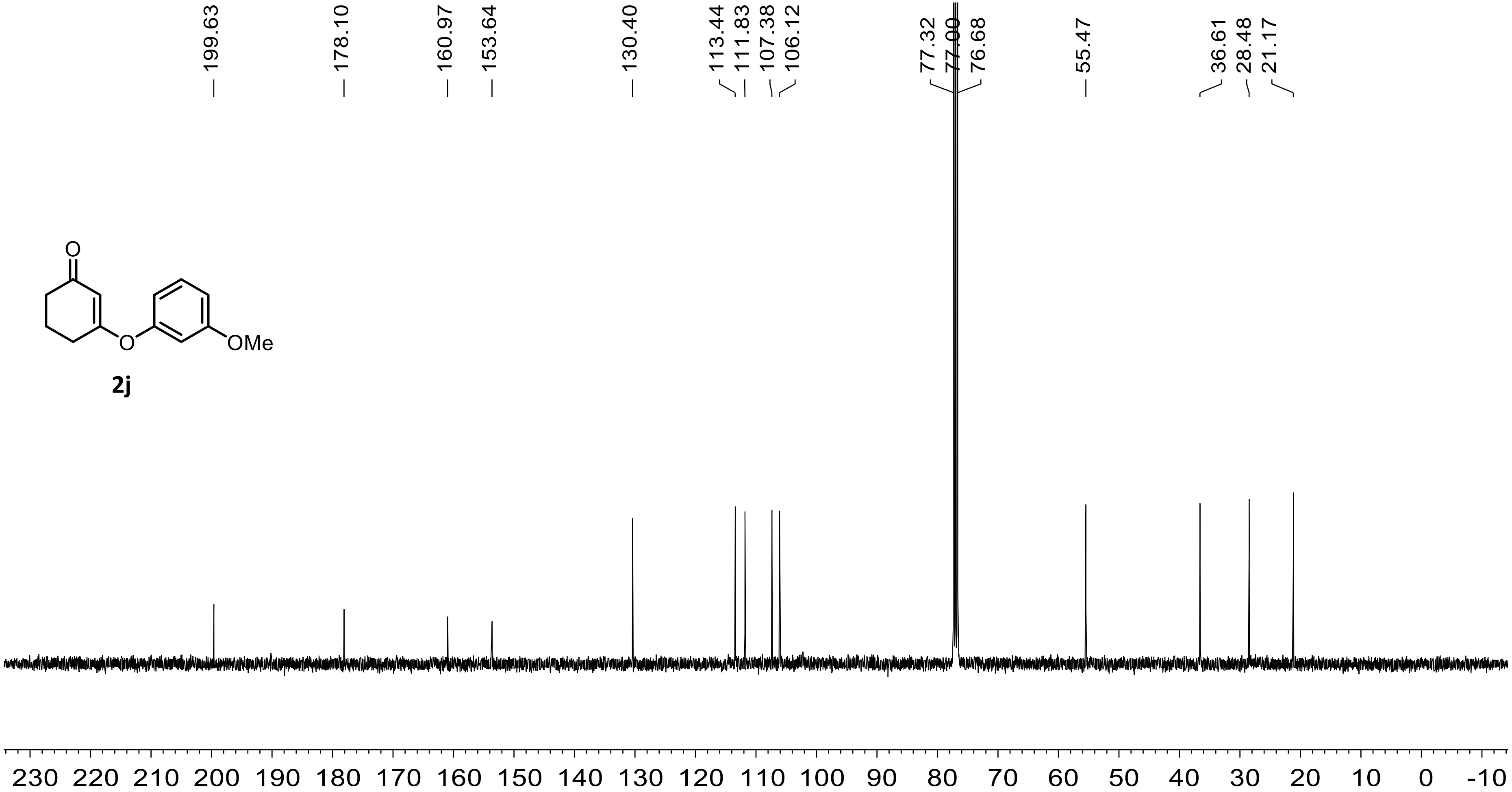

^1H NMR (400 MHz, $CDCl_3$) spectrum of compound 2k

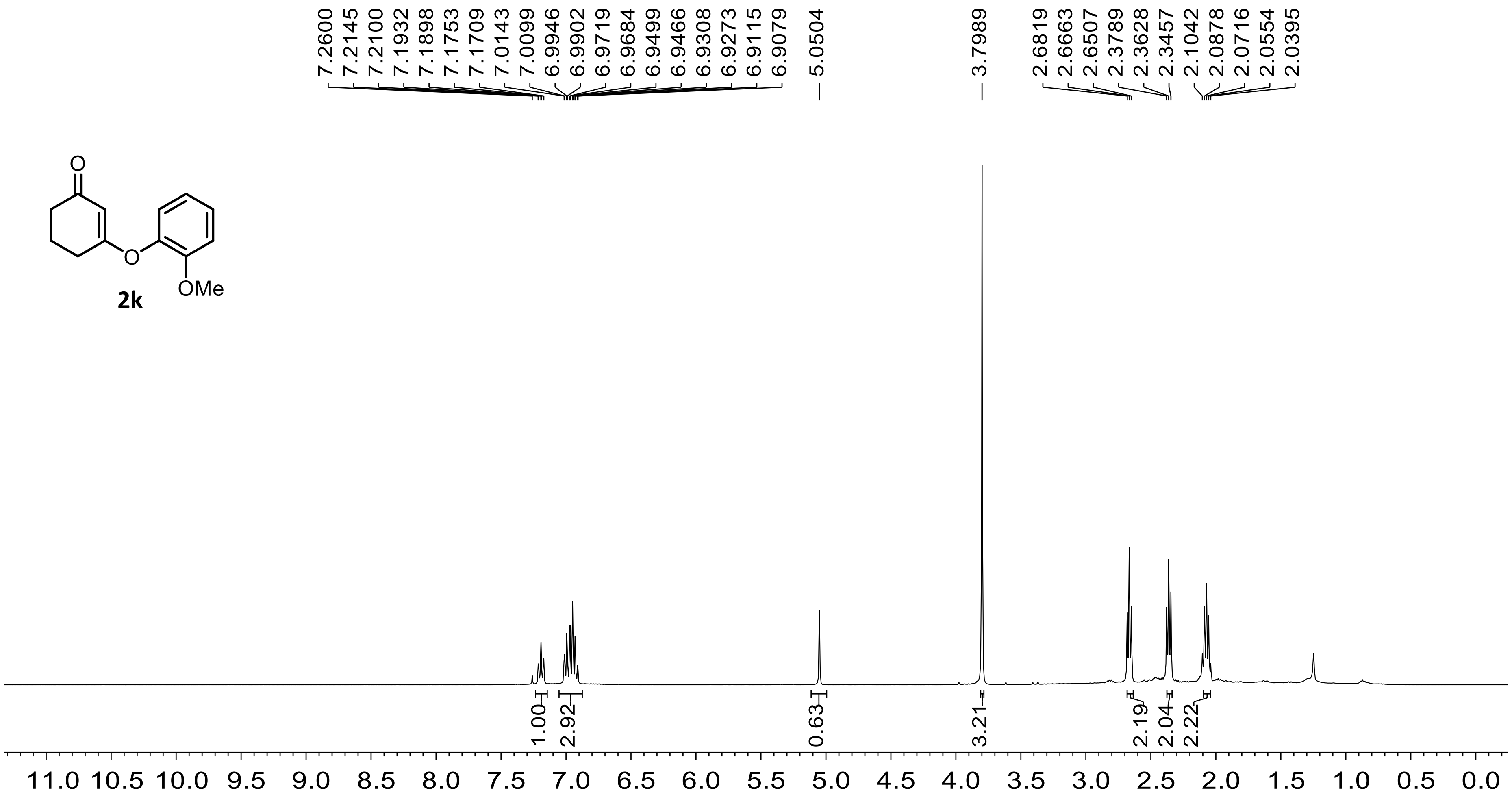

$^{13}C\{^{1}H\}$ NMR (100 MHz, $CDCl_3$) spectrum of compound 2k

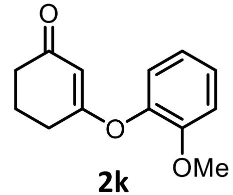

2k

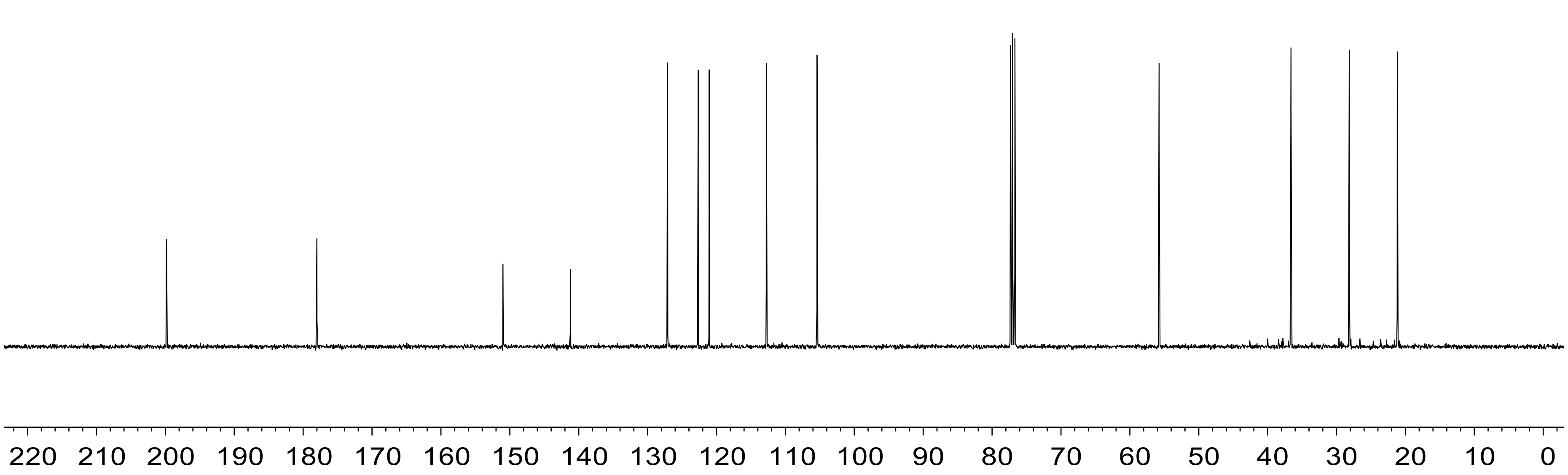

^{1}H NMR (400 MHz, $CDCl_3$) spectrum of compound 2l

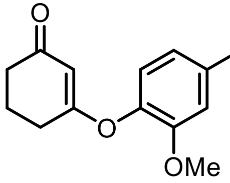

2l

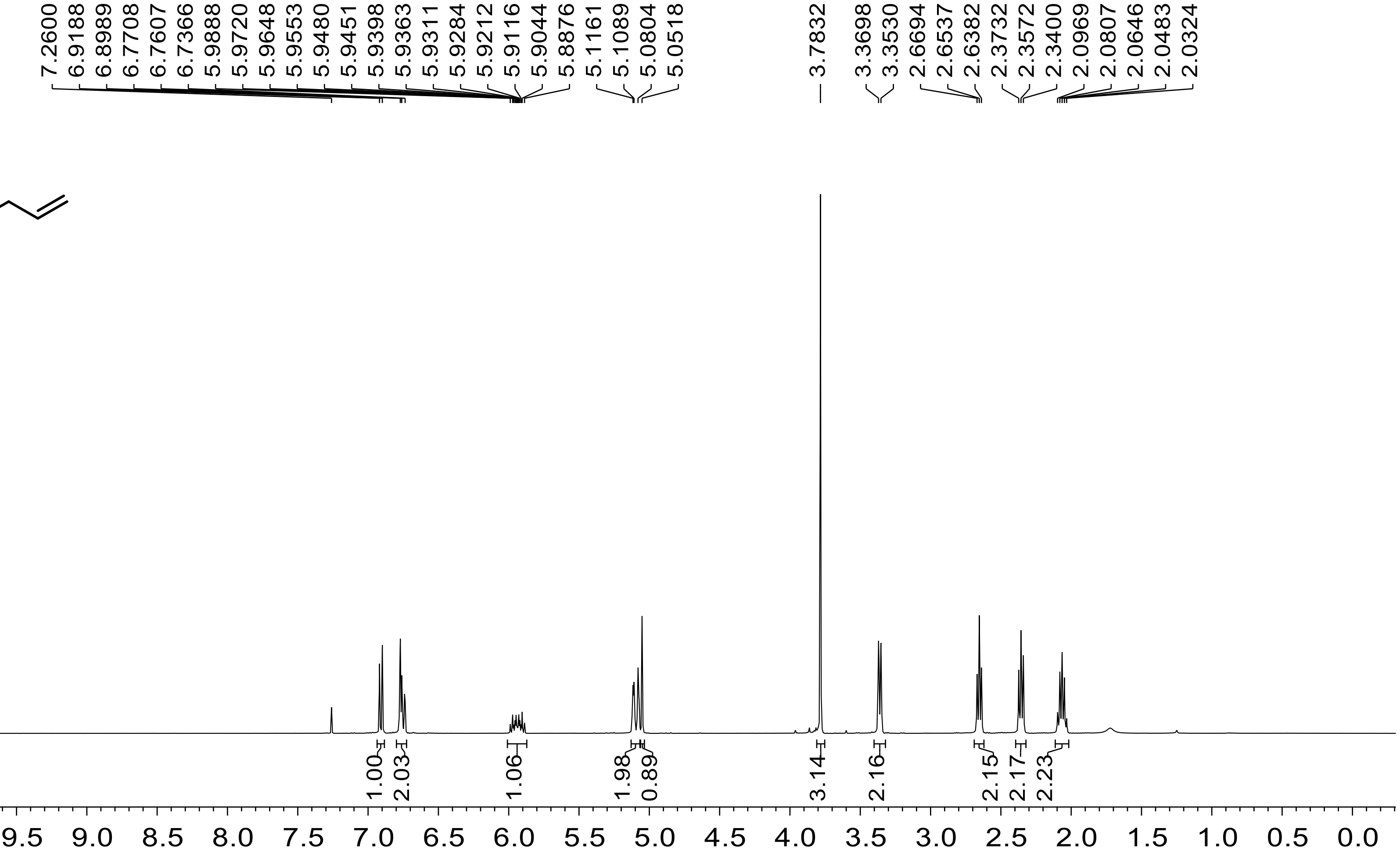

$^{13}C\{^{1}H\}$ NMR (100 MHz, $CDCl_3$) spectrum of compound 2l

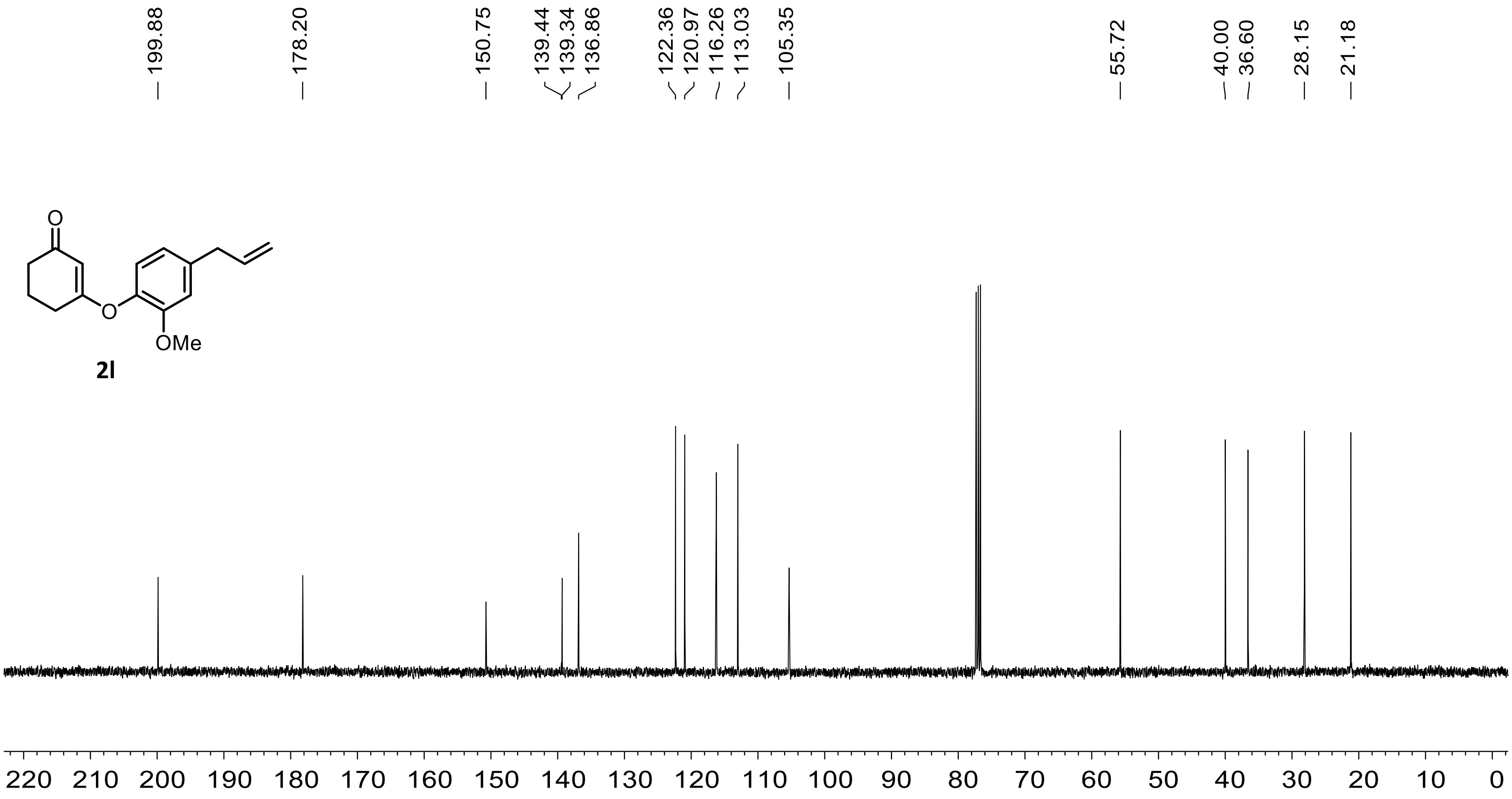

^1H NMR (400 MHz, $CDCl_3$) spectrum of compound 2m

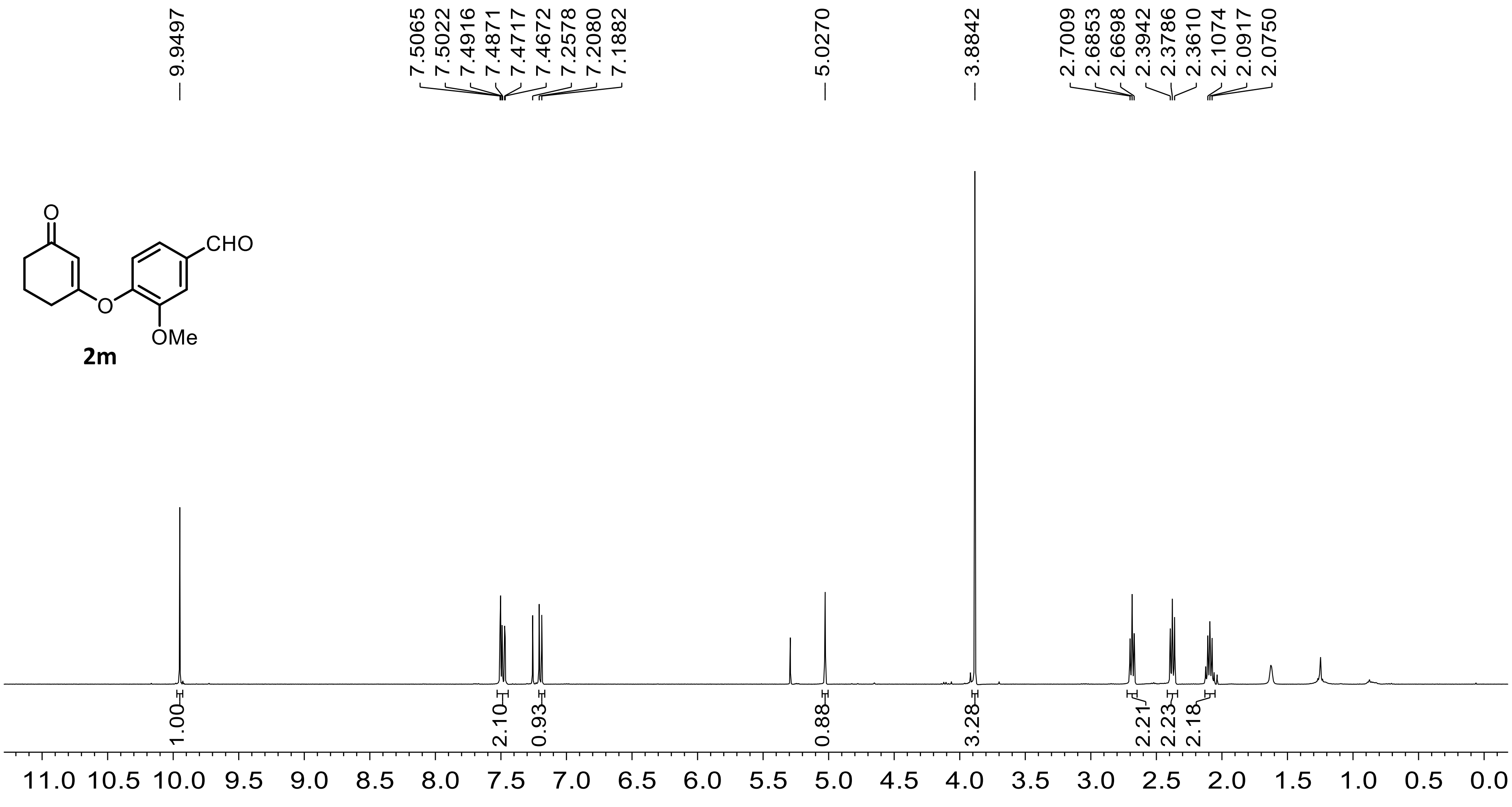

$^{13}C\{^1H\}$ NMR (100 MHz, $CDCl_3$) spectrum of compound 2m

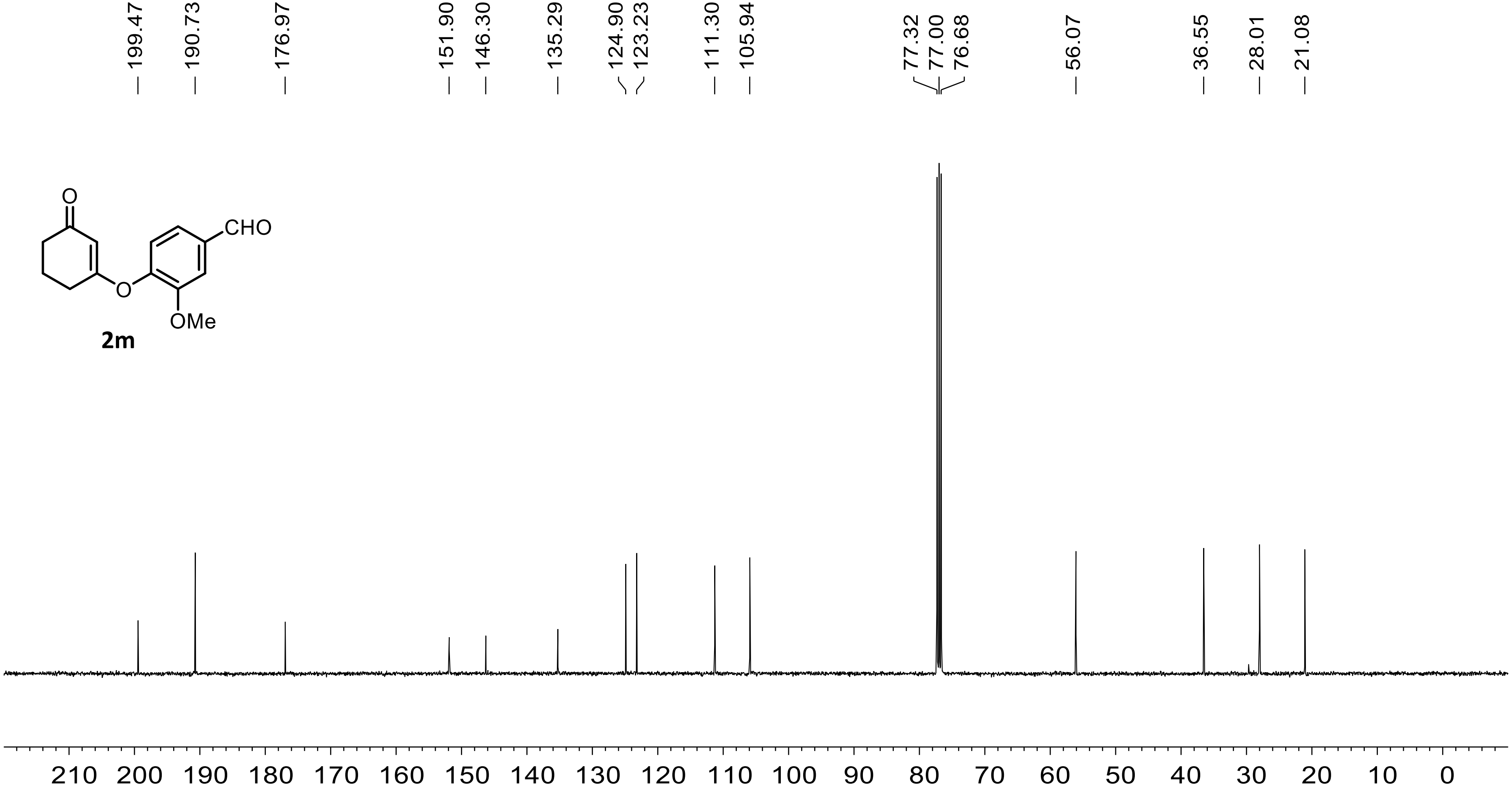

^1H NMR (400 MHz, $CDCl_3$) spectrum of compound 2n

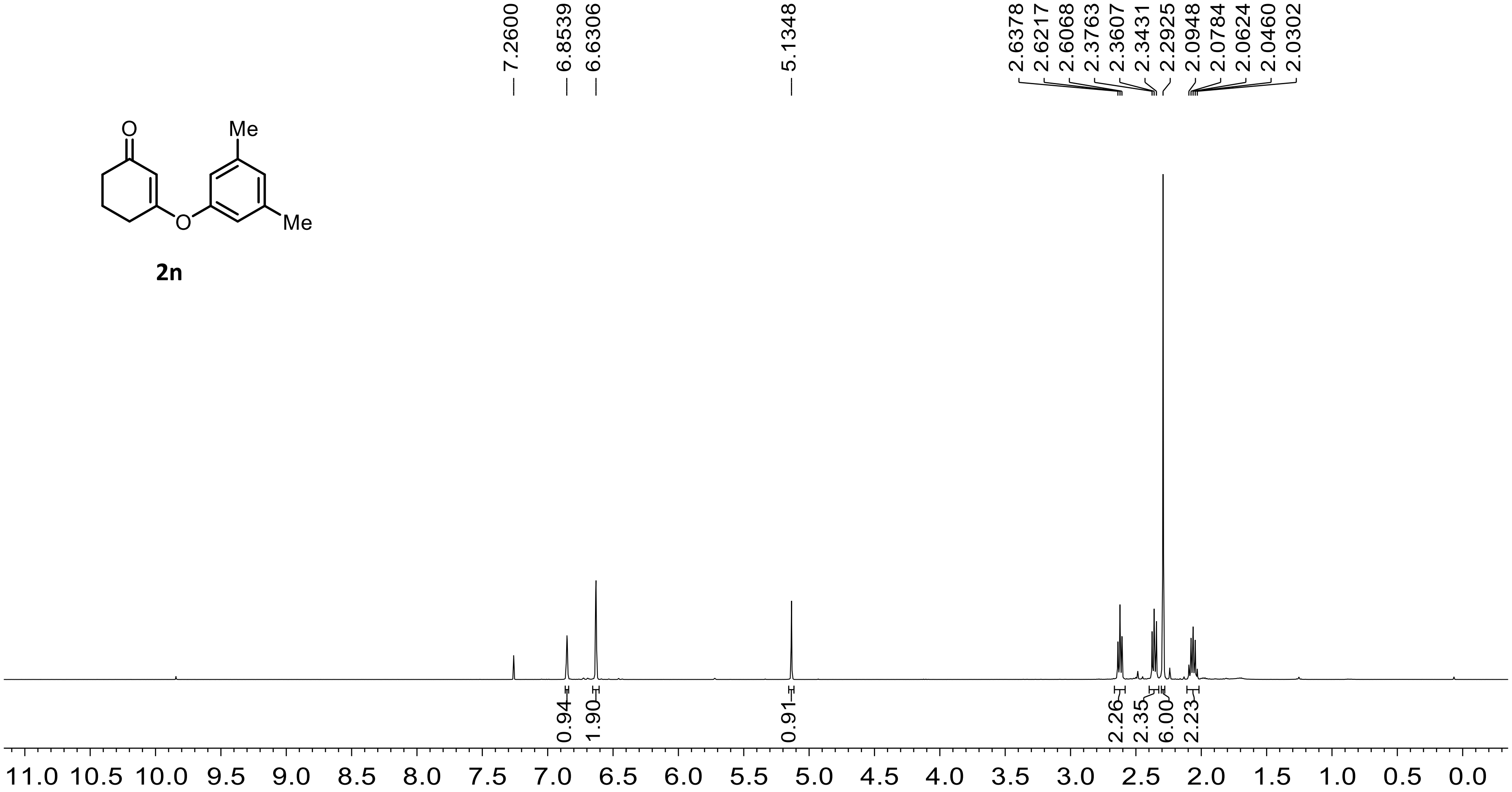

2n

$^{13}C\{^1H\}$ NMR (100 MHz, $CDCl_3$) spectrum of compound 2n

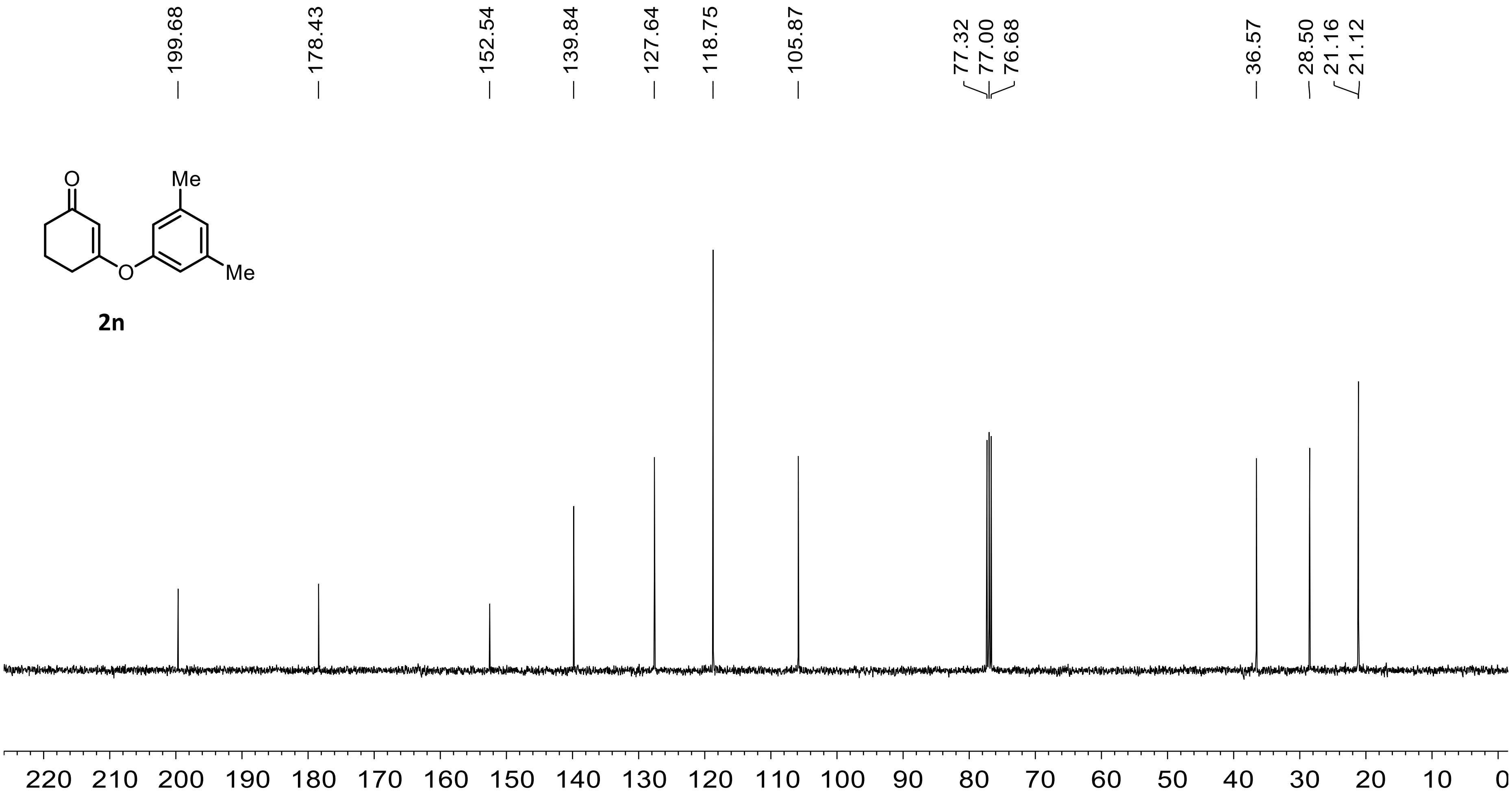

^{1}H NMR (400 MHz, $CDCl_3$) spectrum of compound 2o

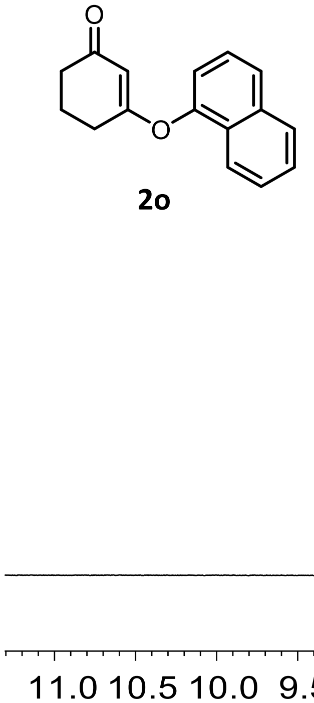

2o

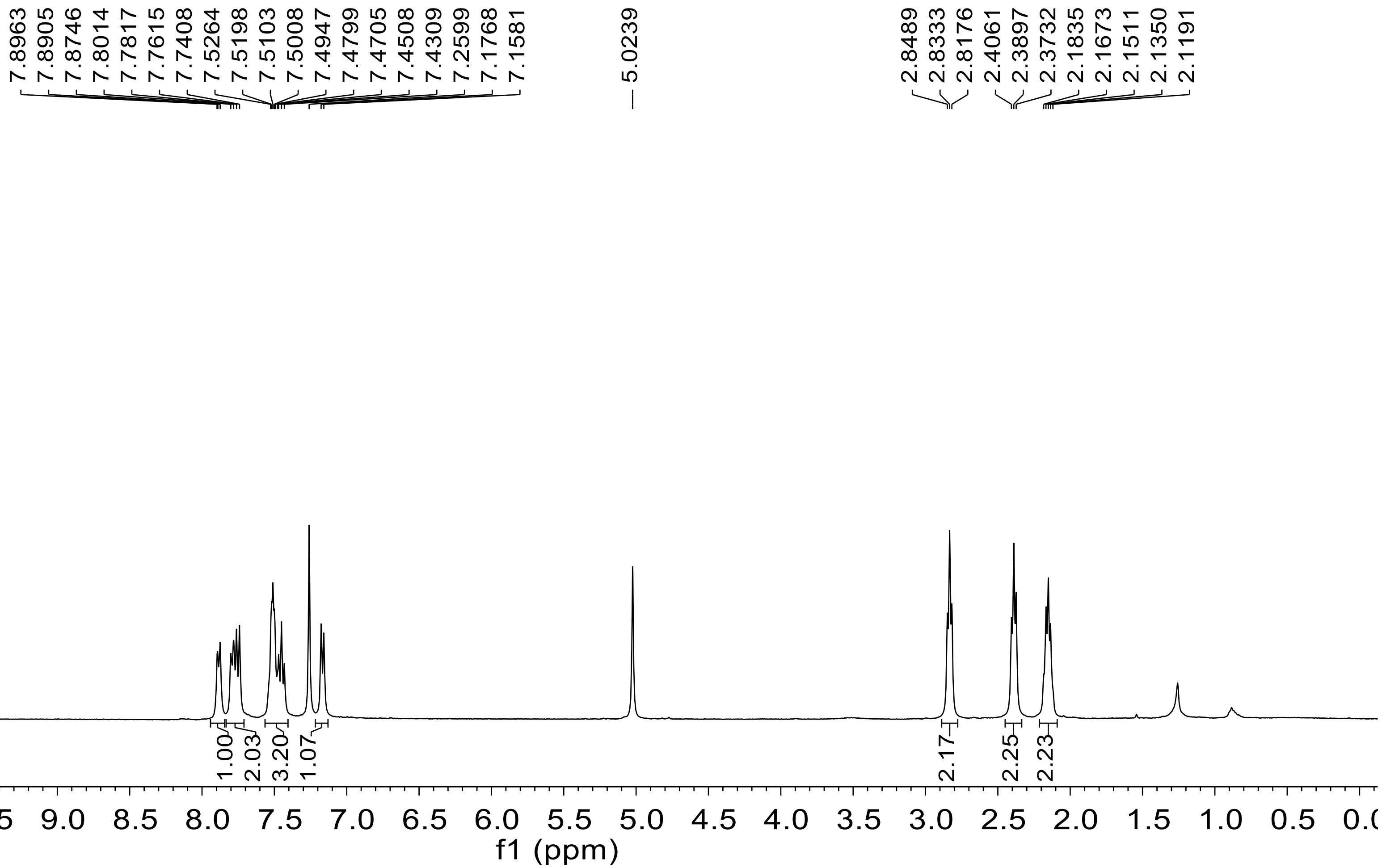

$^{13}C\{^{1}H\}$ NMR (100 MHz, $CDCl_3$) spectrum of compound 2o

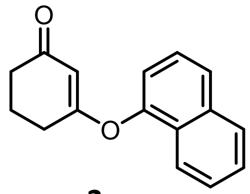

2o

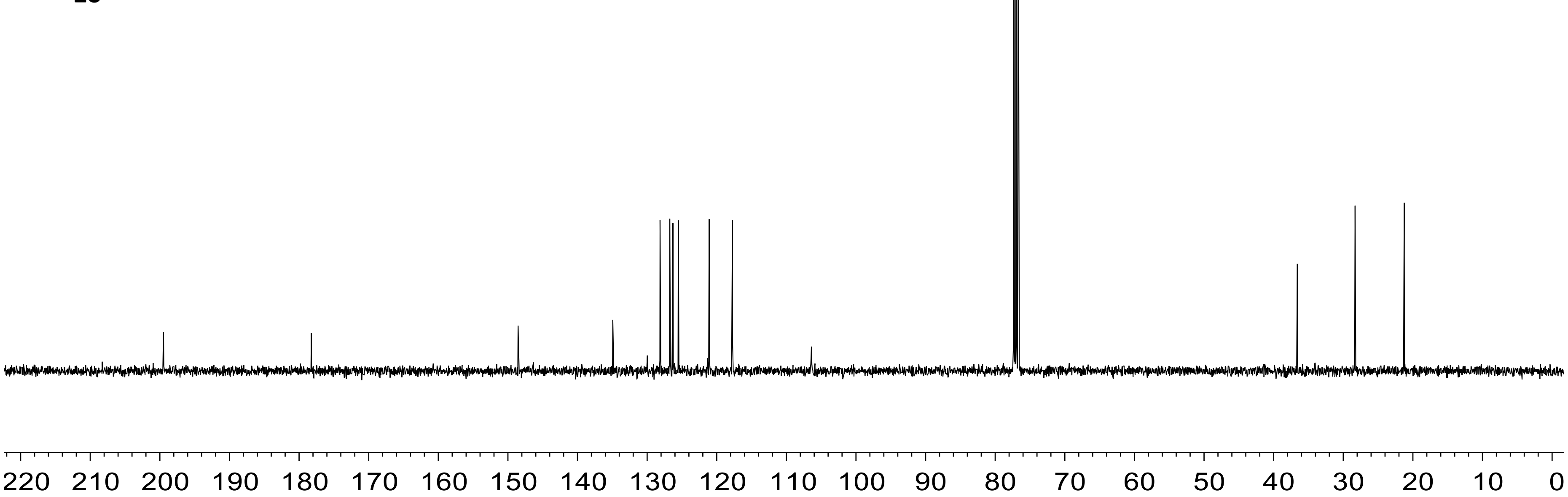

^{1}H NMR (400 MHz, $CDCl_3$) spectrum of compound 2p

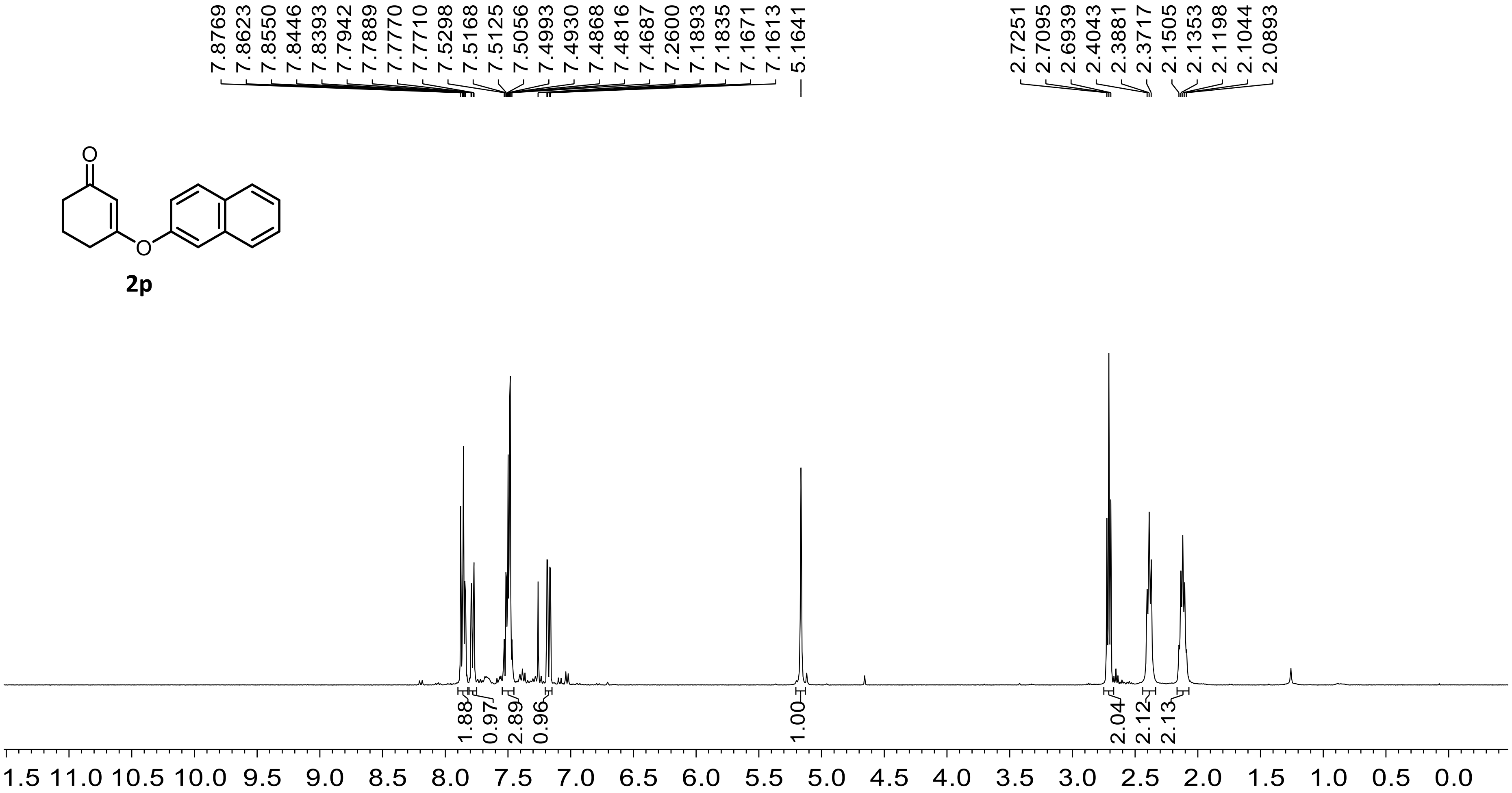

$^{13}C\{^1H\}$ NMR (100 MHz, $CDCl_3$) spectrum of compound 2p

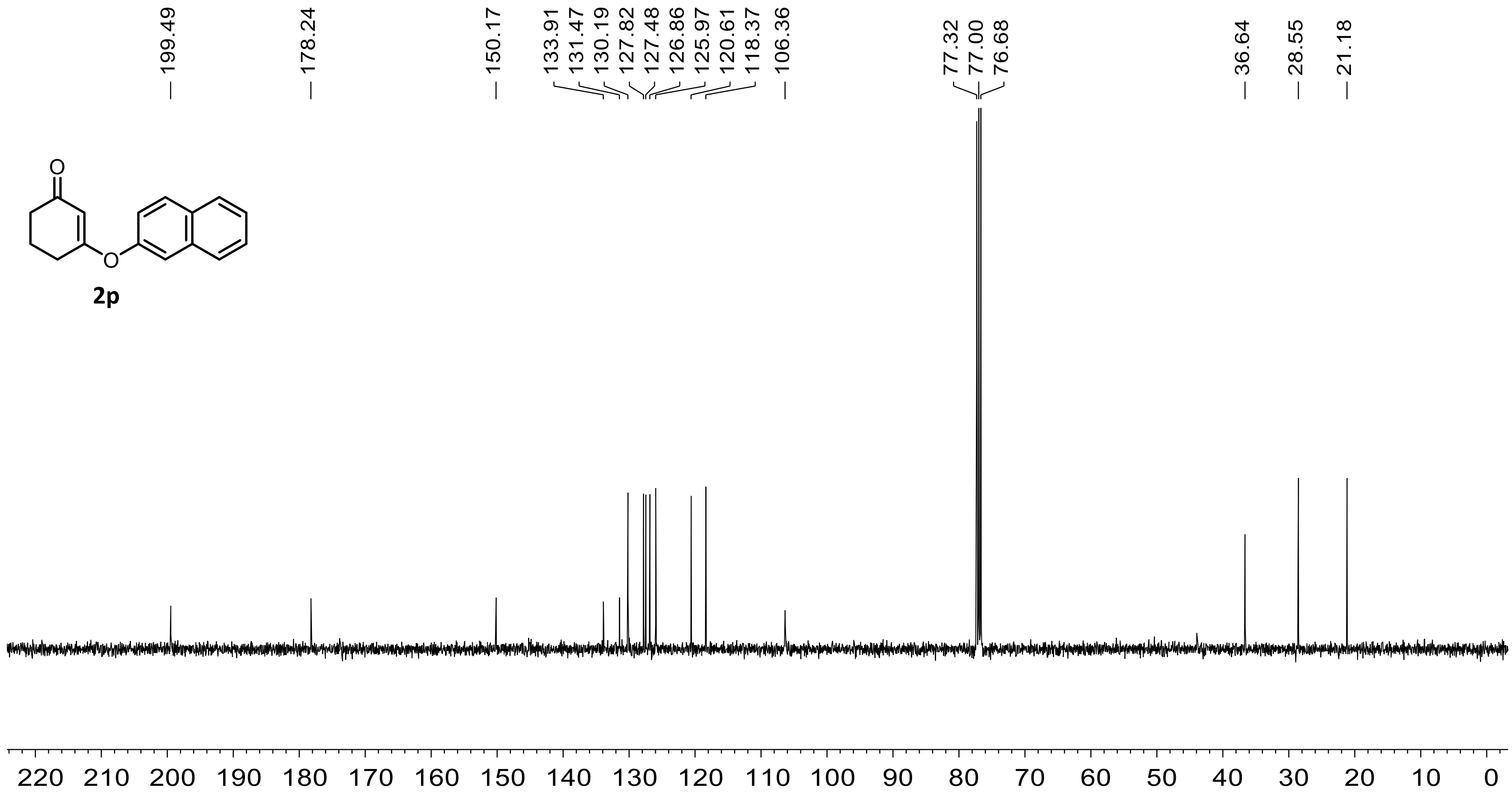

^{1}H NMR (400 MHz, $CDCl_3$) spectrum of compound 2q

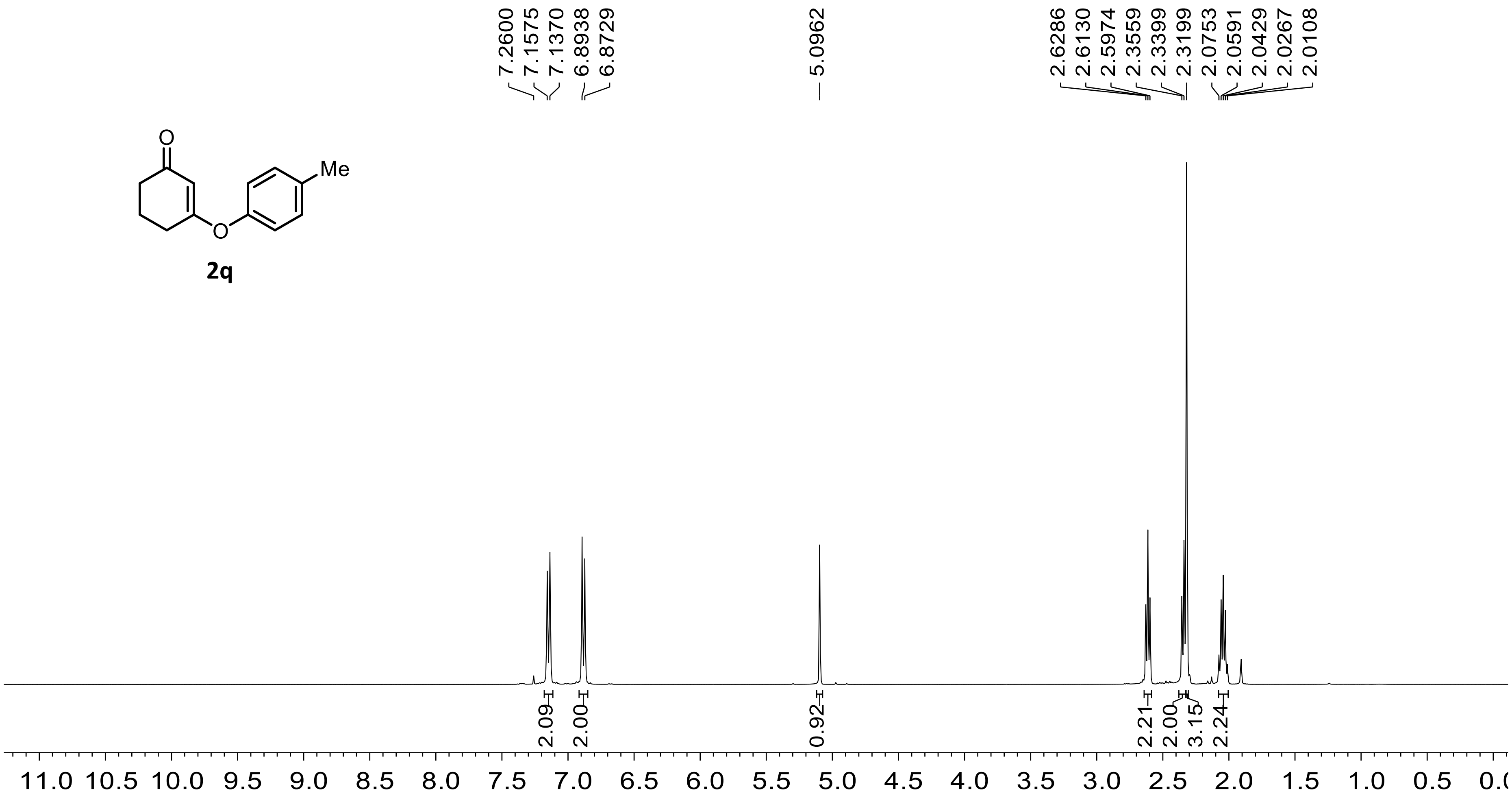

$^{13}C\{^1H\}$ NMR (100 MHz, $CDCl_3$) spectrum of compound 2q

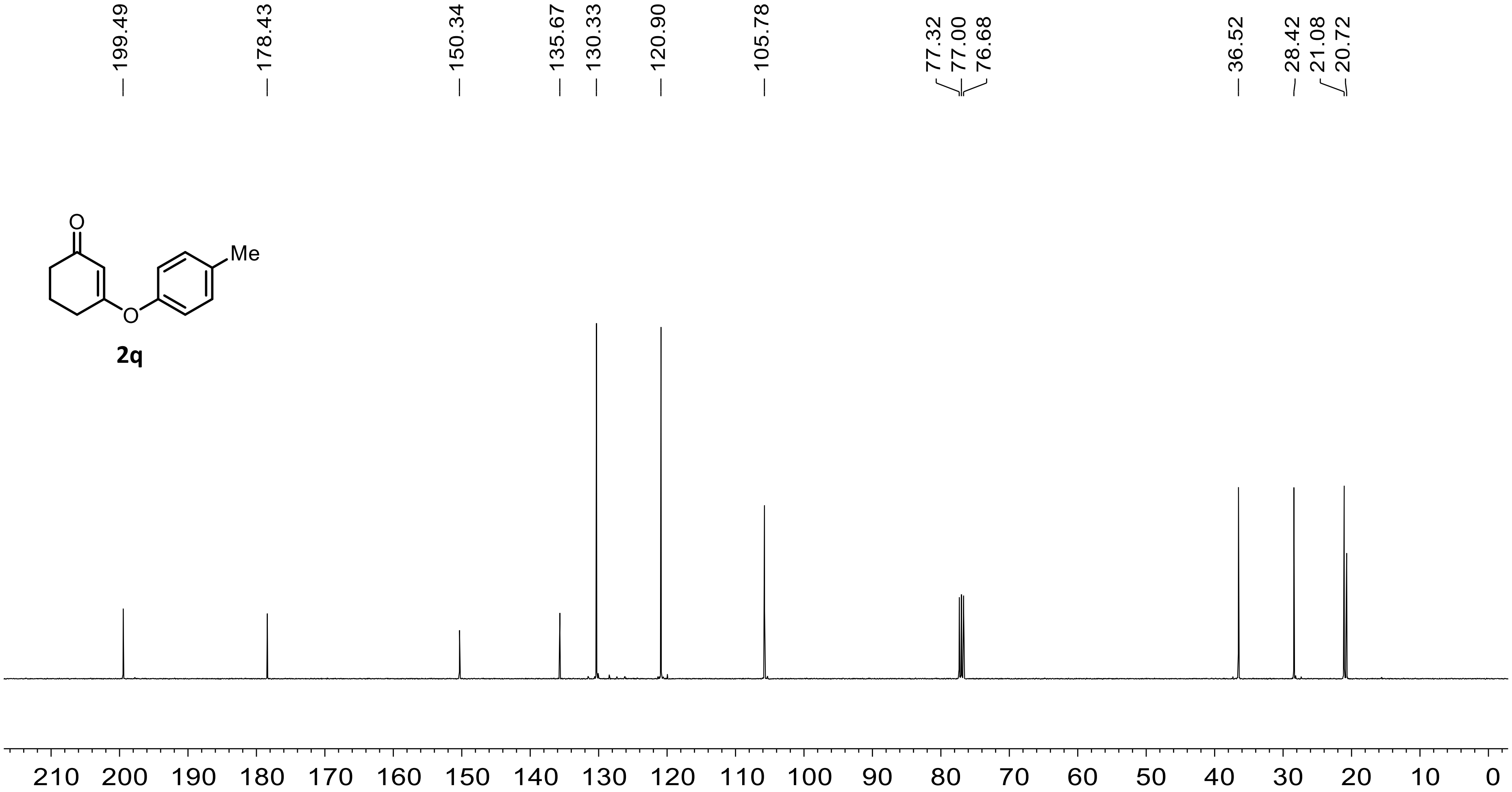

^1H NMR (400 MHz, $CDCl_3$) spectrum of compound 2r

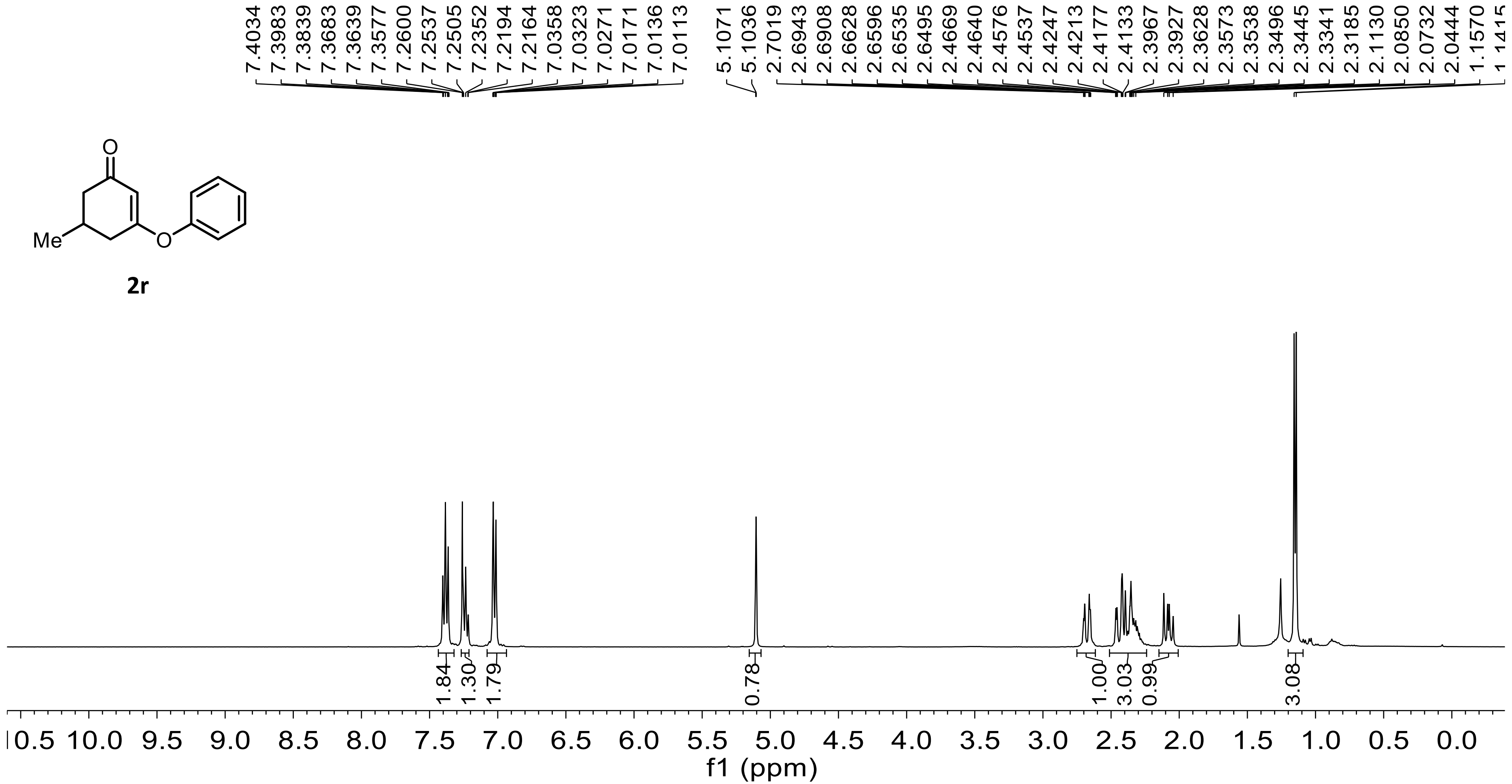

$^{13}C\{^1H\}$ NMR (100 MHz, $CDCl_3$) spectrum of compound 2r

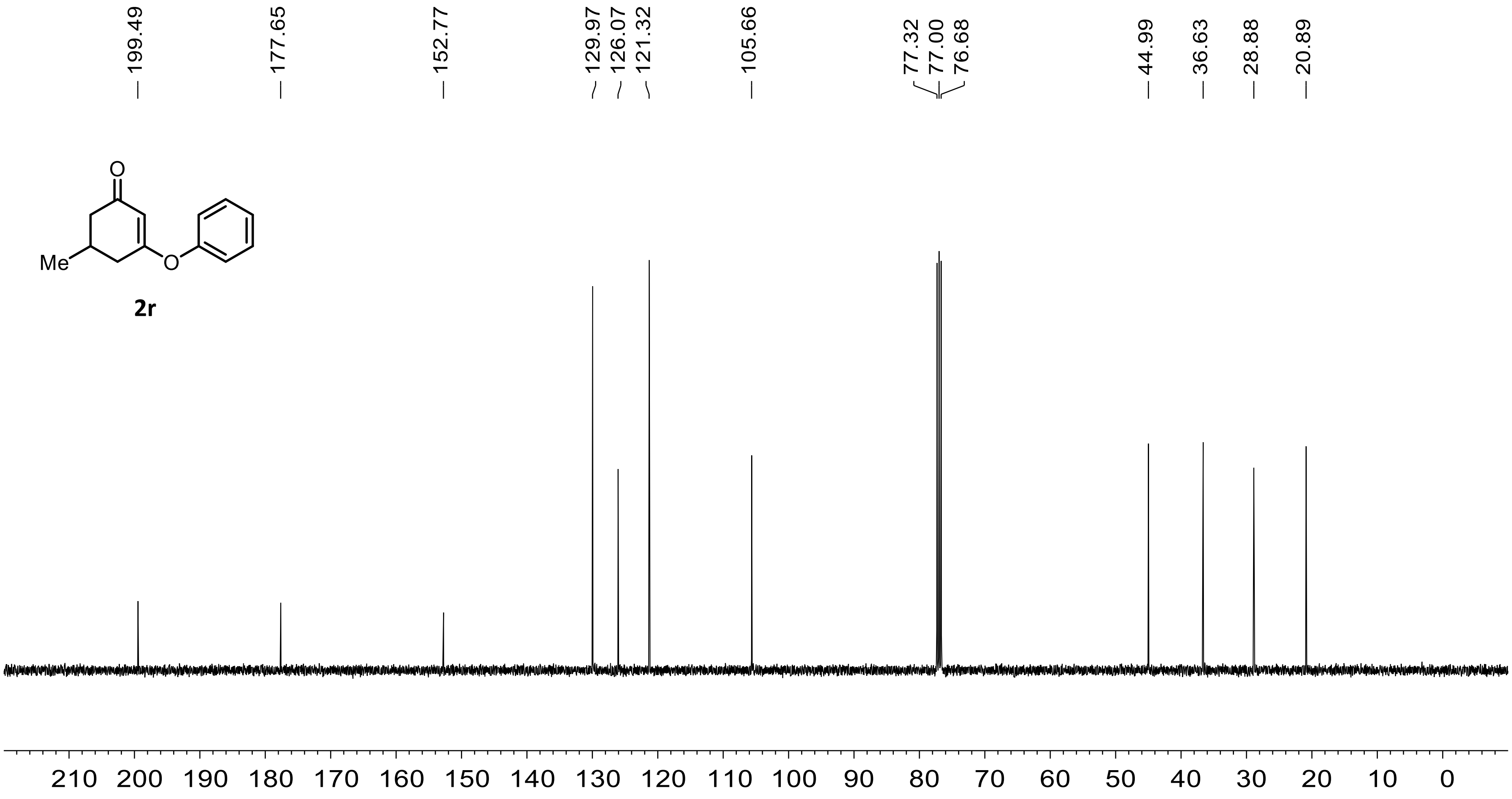

^{1}H NMR (400 MHz, $CDCl_3$) spectrum of compound 2s

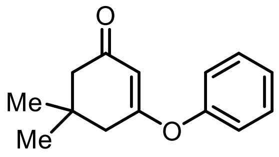

2s

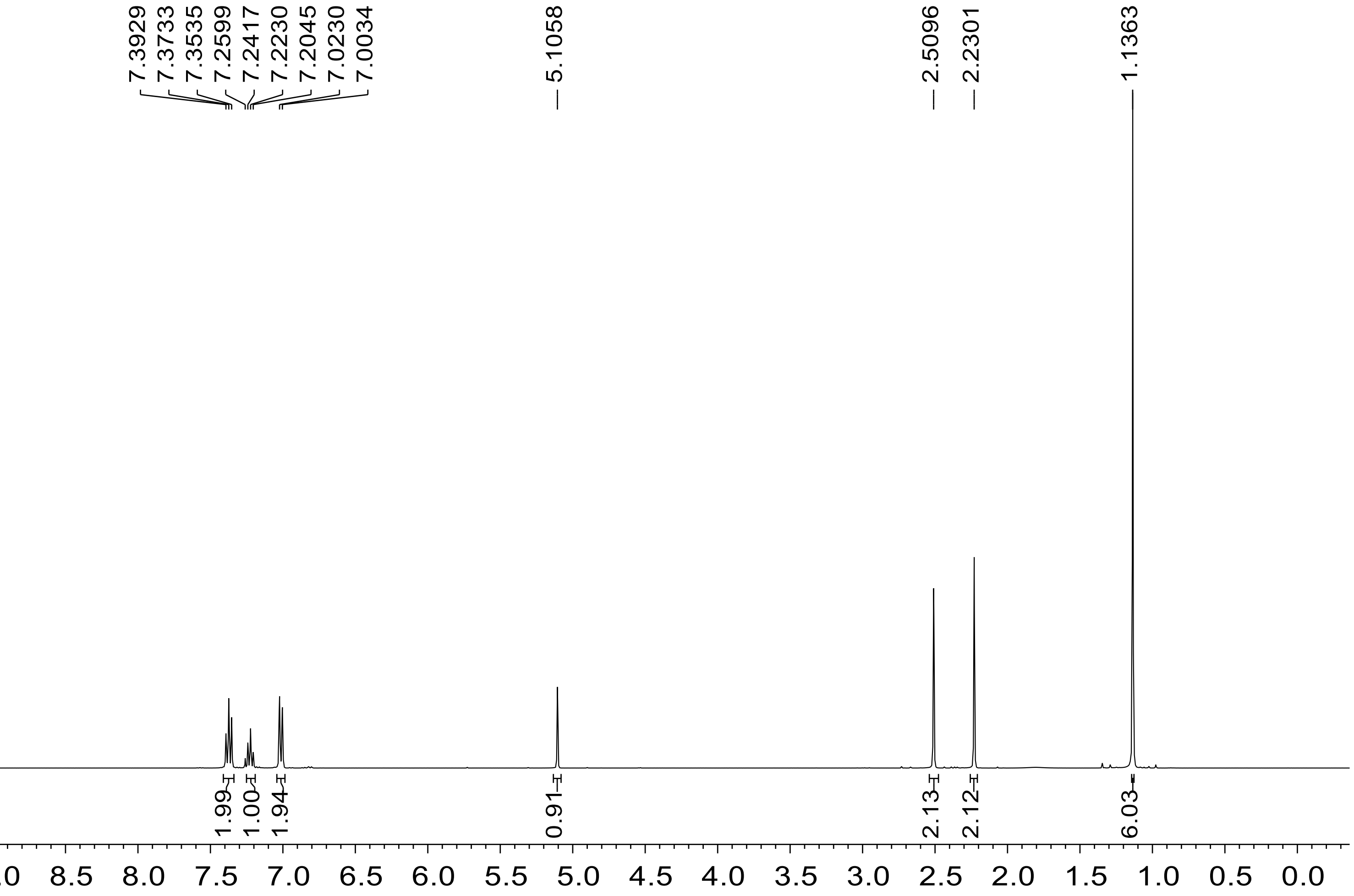

^{1}H NMR (400 MHz, $CDCl_3$) spectrum of compound 3a

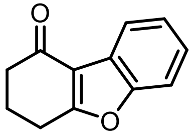

3a

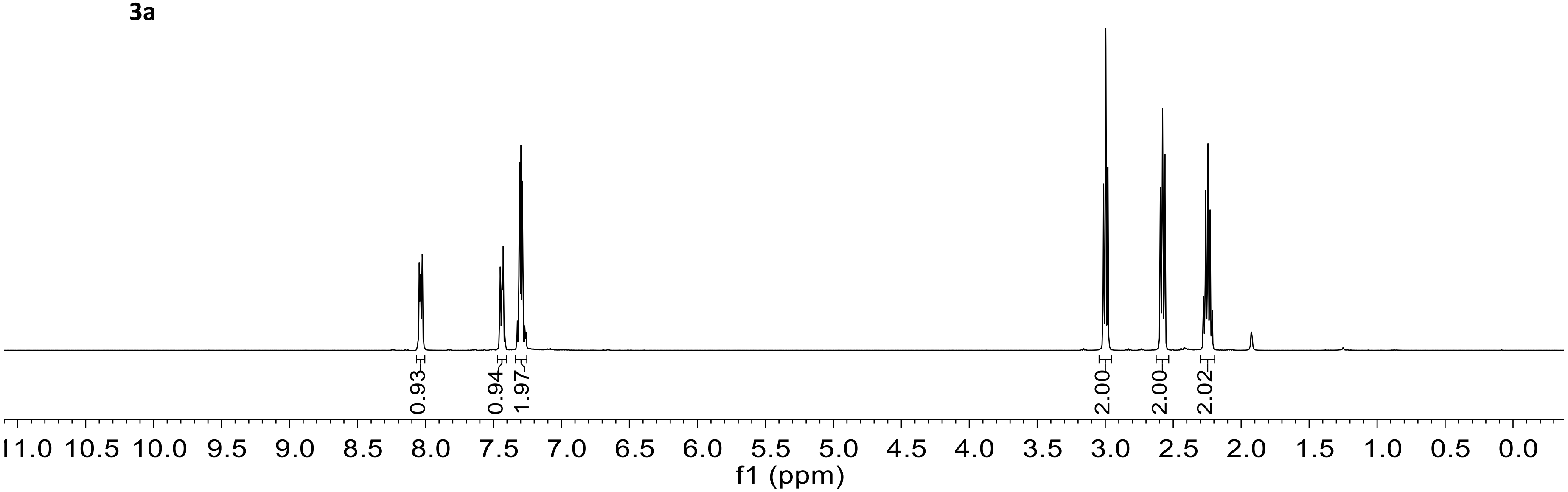

^1H NMR (400 MHz, $CDCl_3$) spectrum of compound 3b

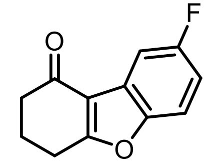

3b

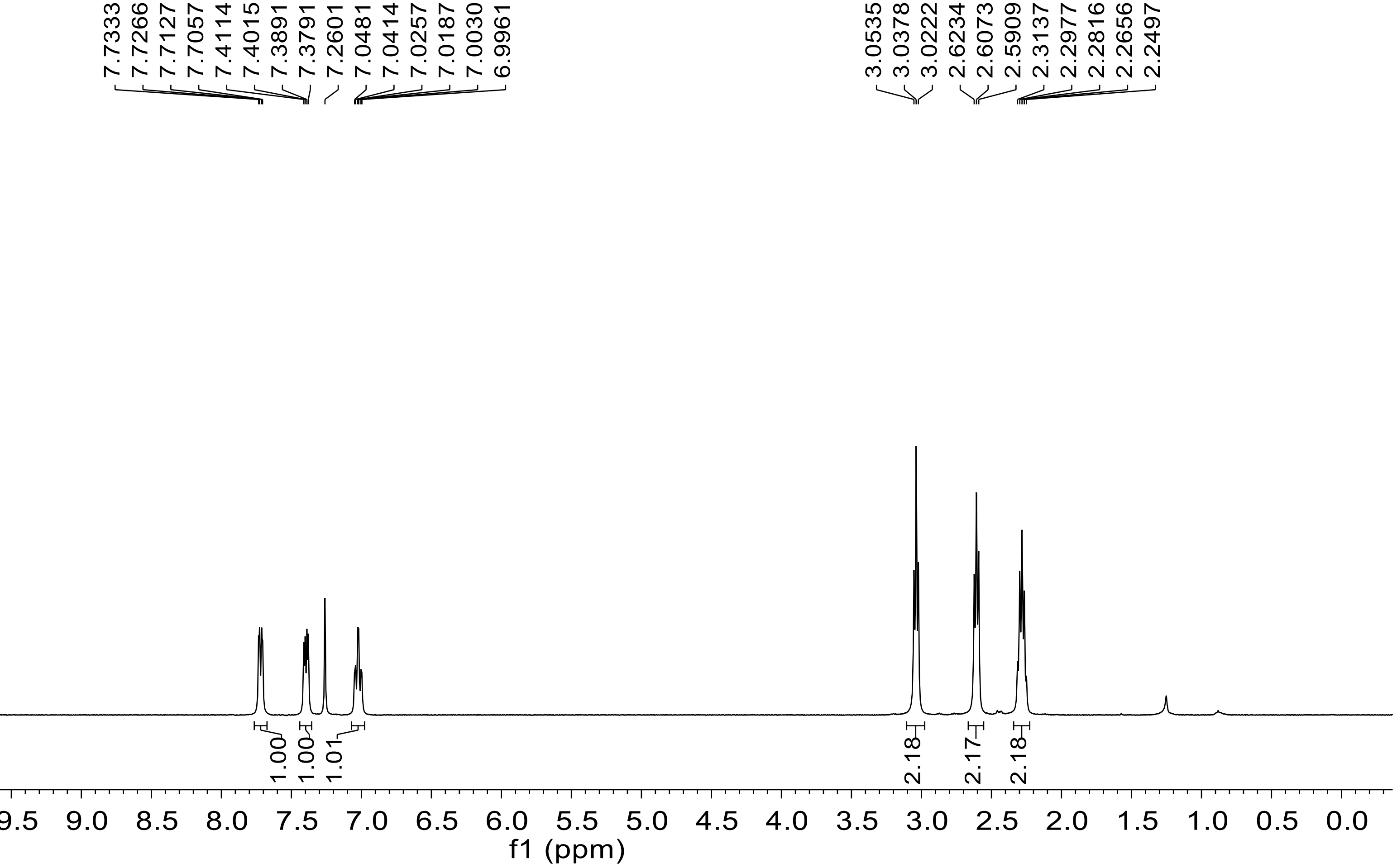

$^{13}C\{^1H\}$ NMR (100 MHz, $CDCl_3$) spectrum of compound 3b

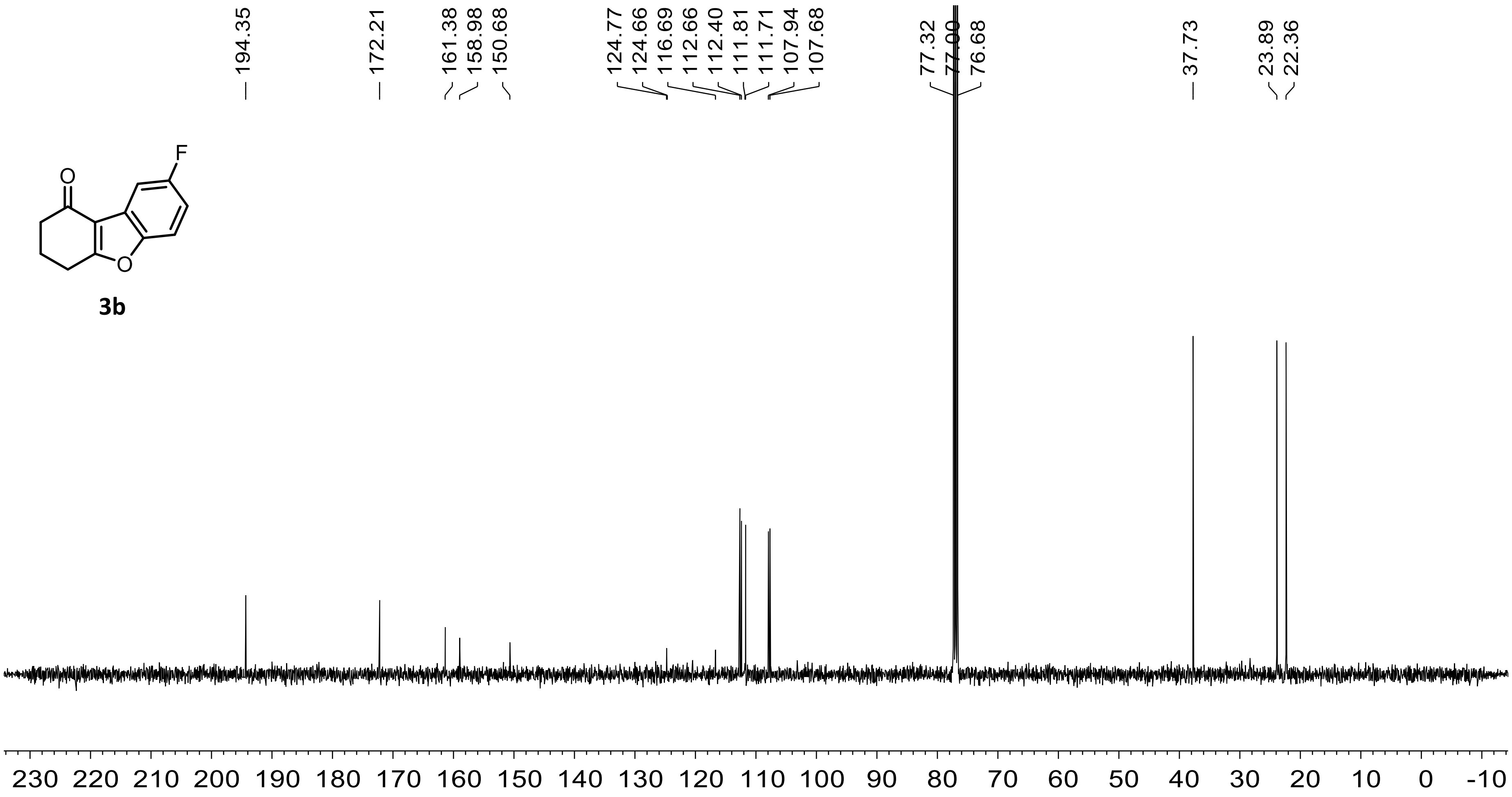

^1H NMR (400 MHz, $CDCl_3$) spectrum of compound 3c

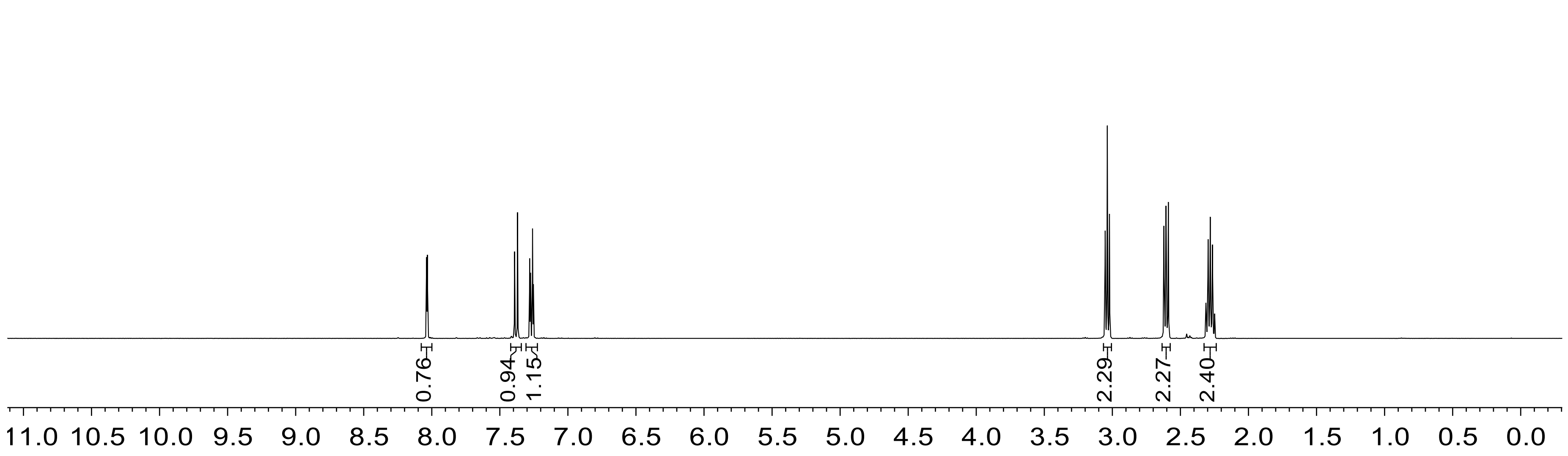

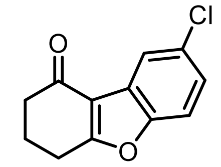

3c

2i

$^{13}C\{^1H\}$ NMR (100 MHz, $CDCl_3$) spectrum of compound 3c

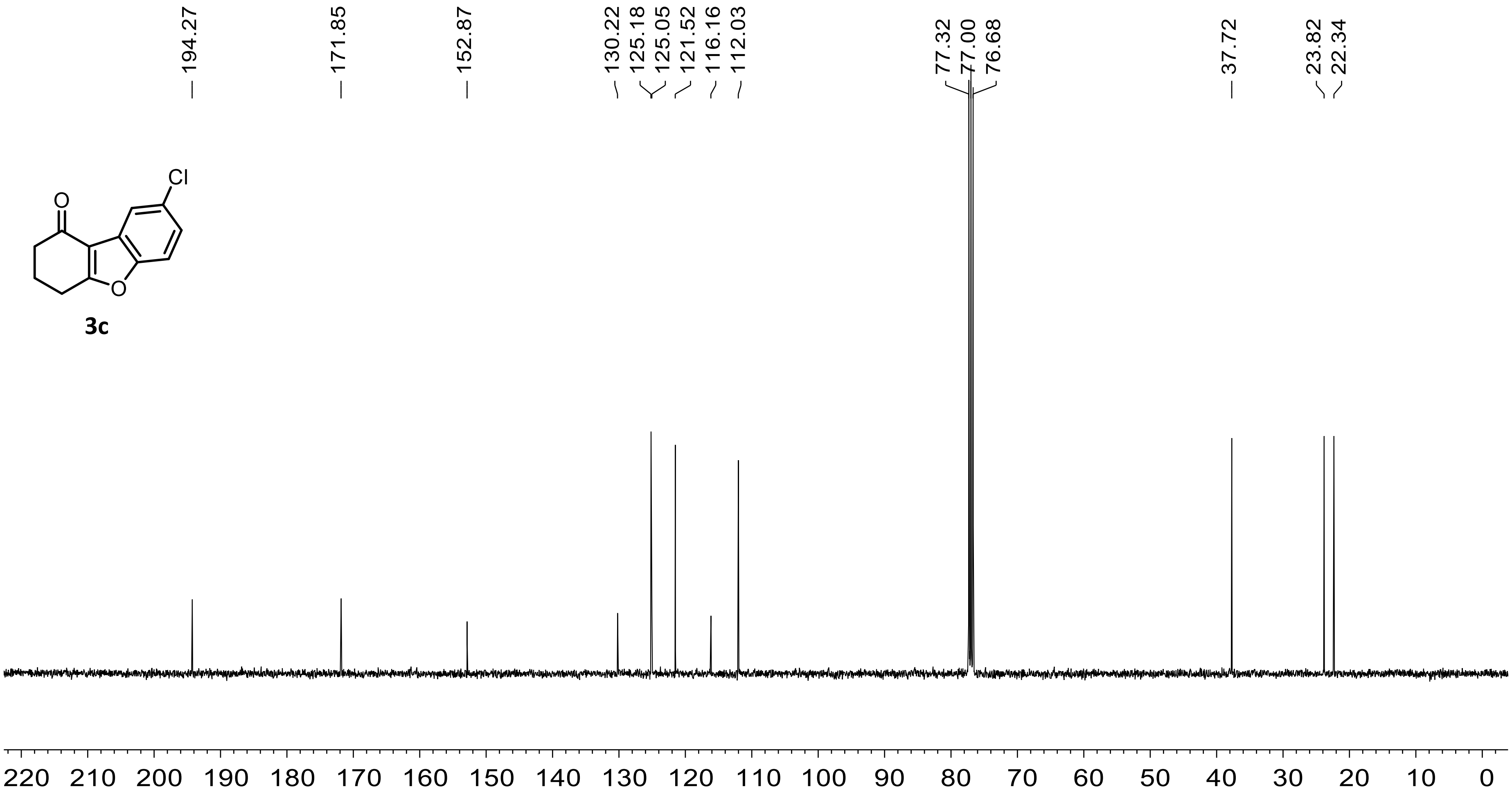

^1H NMR (400 MHz, $CDCl_3$) spectrum of compound 3d

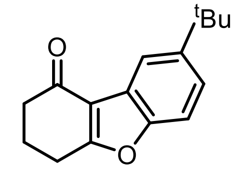

3d

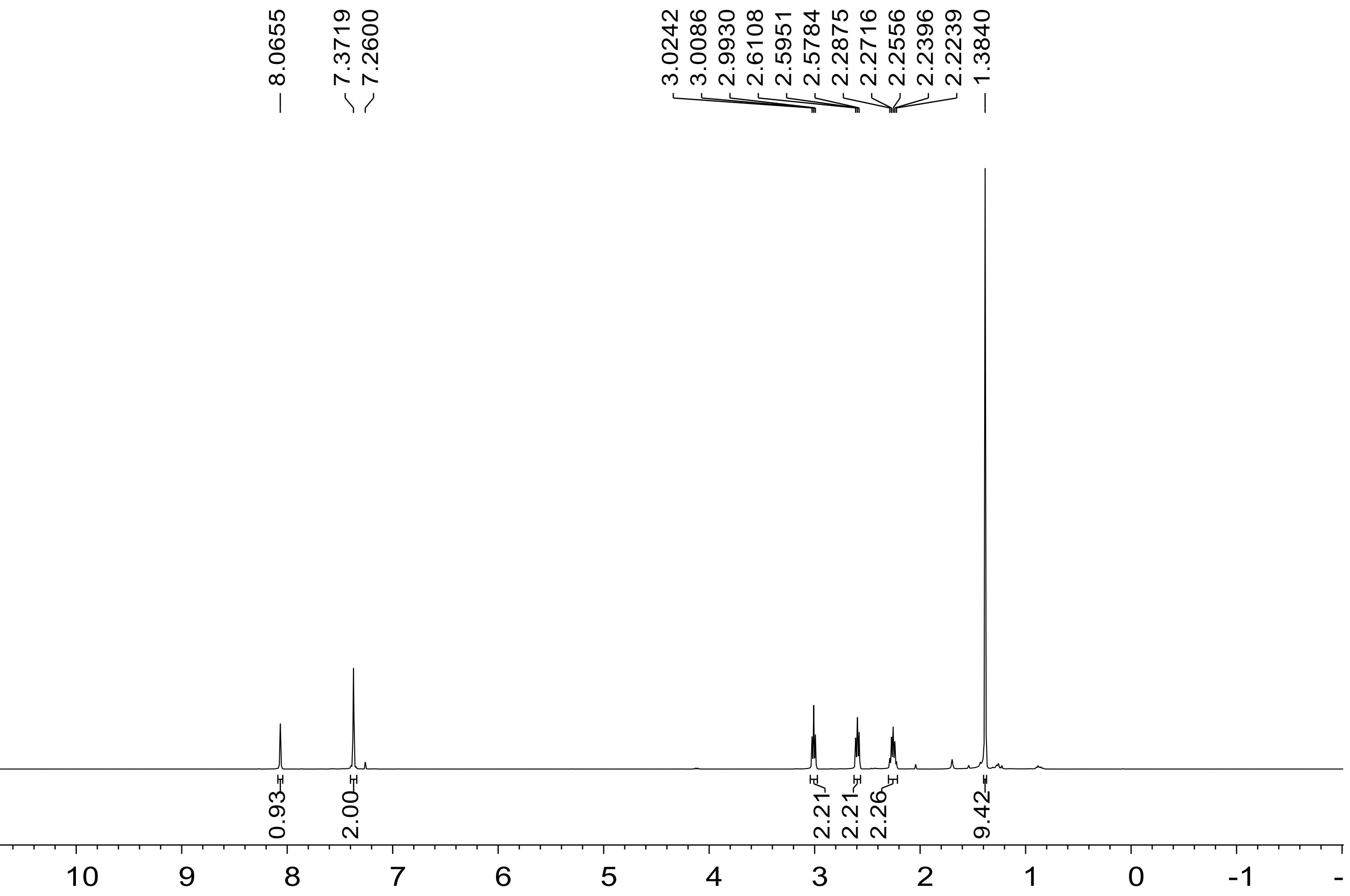

$^{13}C\{^{1}H\}$ NMR (100 MHz, $CDCl_3$) spectrum of compound 3d

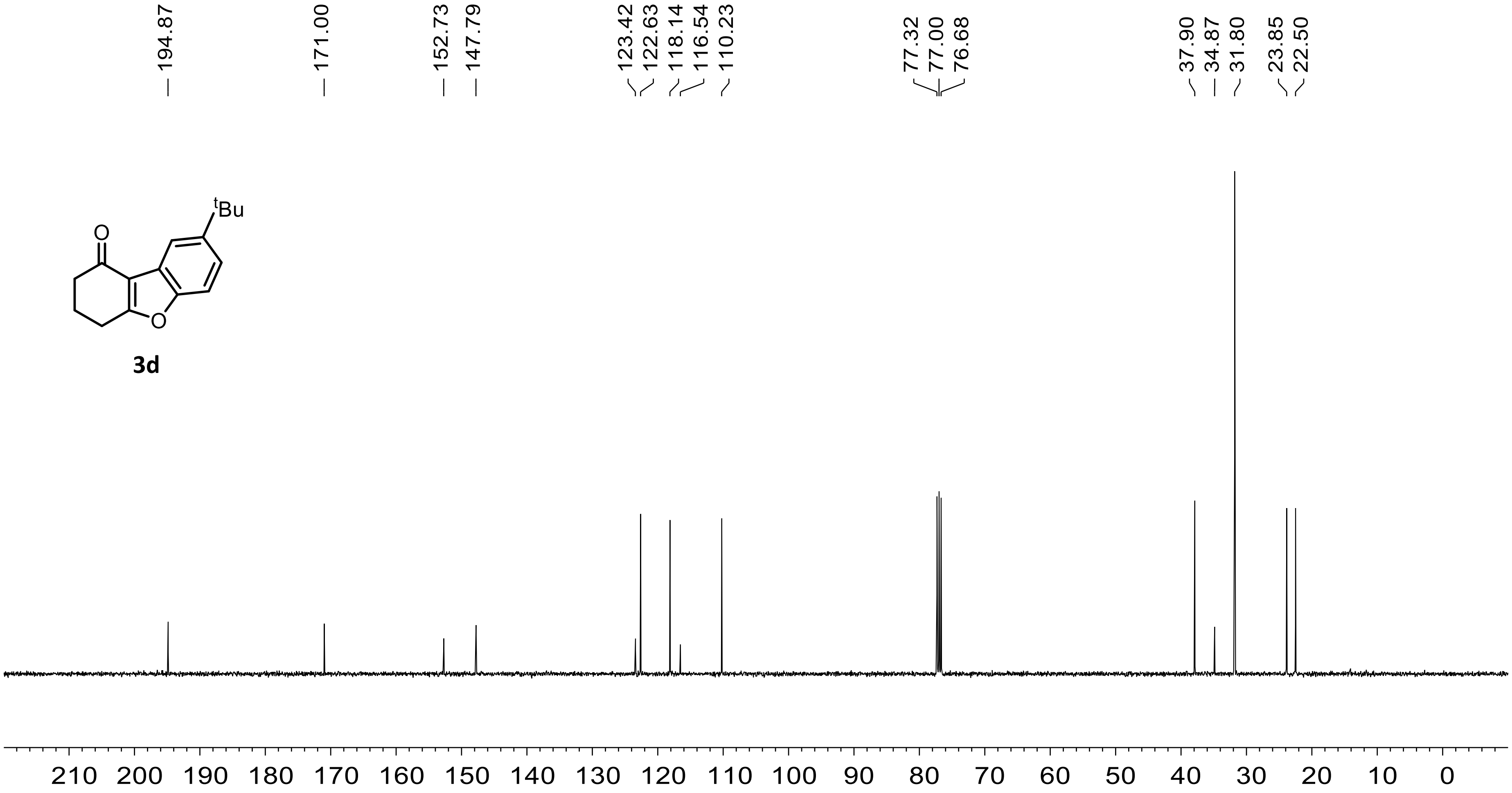

^{1}H NMR (400 MHz, $CDCl_3$) spectrum of compound 3e

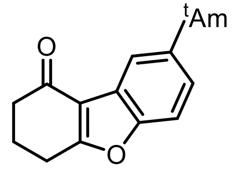

3e

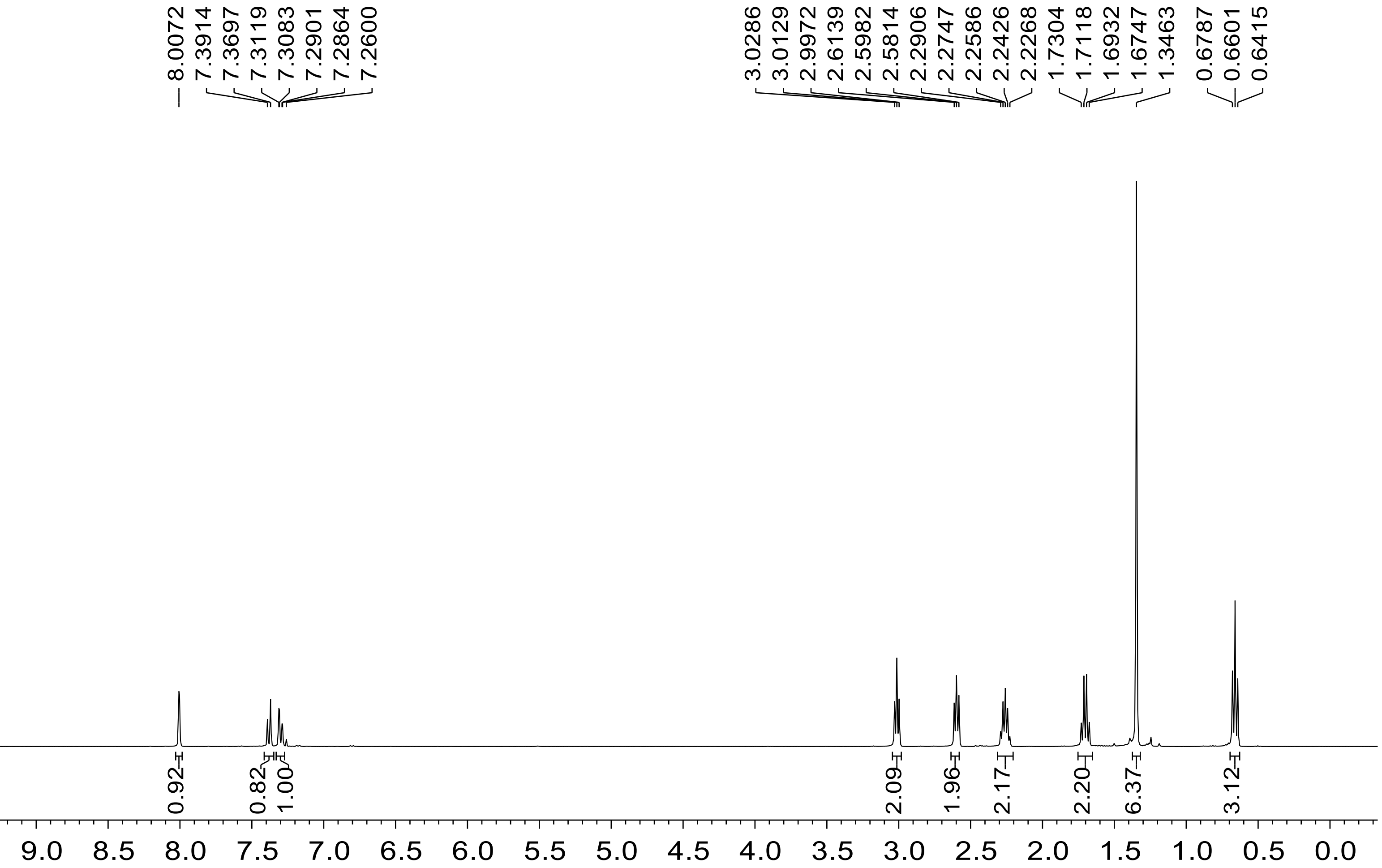

3e

$^{13}C\{^1H\}$ NMR (100 MHz, $CDCl_3$) spectrum of compound 3e

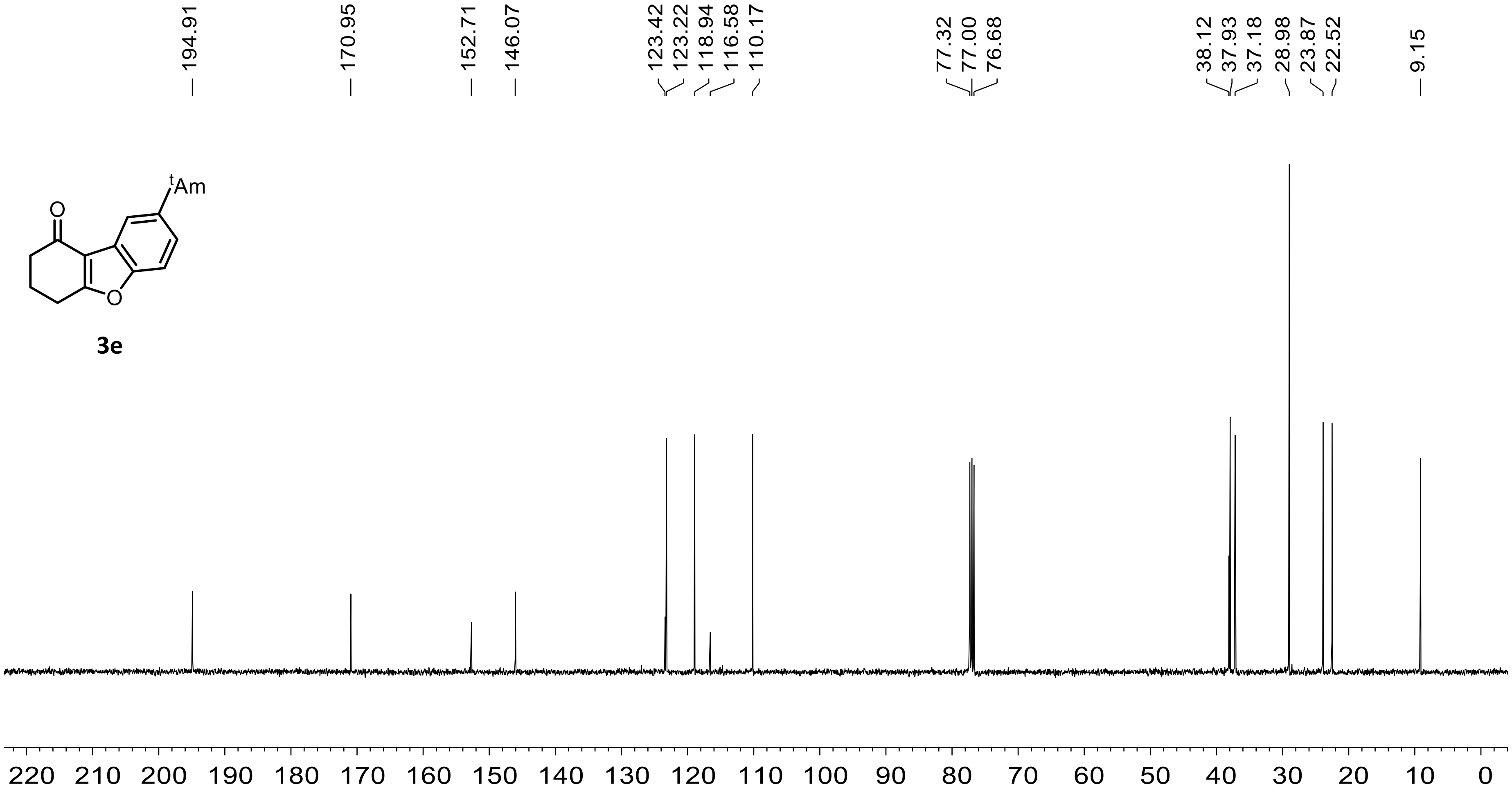

^{1}H NMR (400 MHz, $CDCl_3$) spectrum of compound 3f

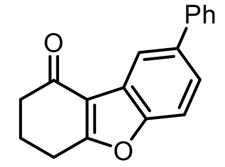

3f

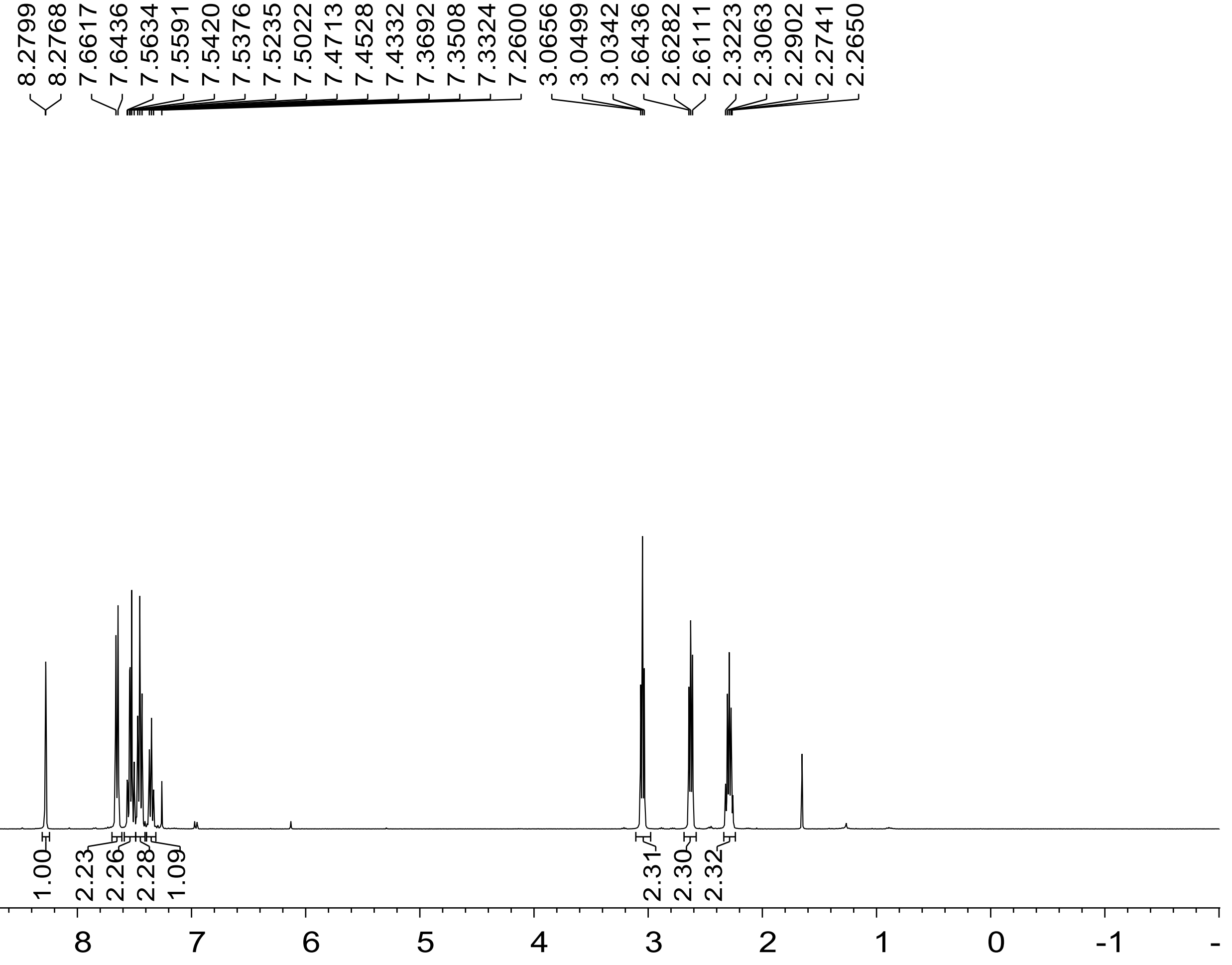

3f

$^{13}C\{^1H\}$ NMR (100 MHz, $CDCl_3$) spectrum of compound 3f

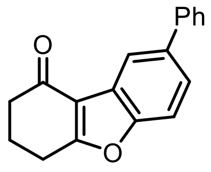

3f

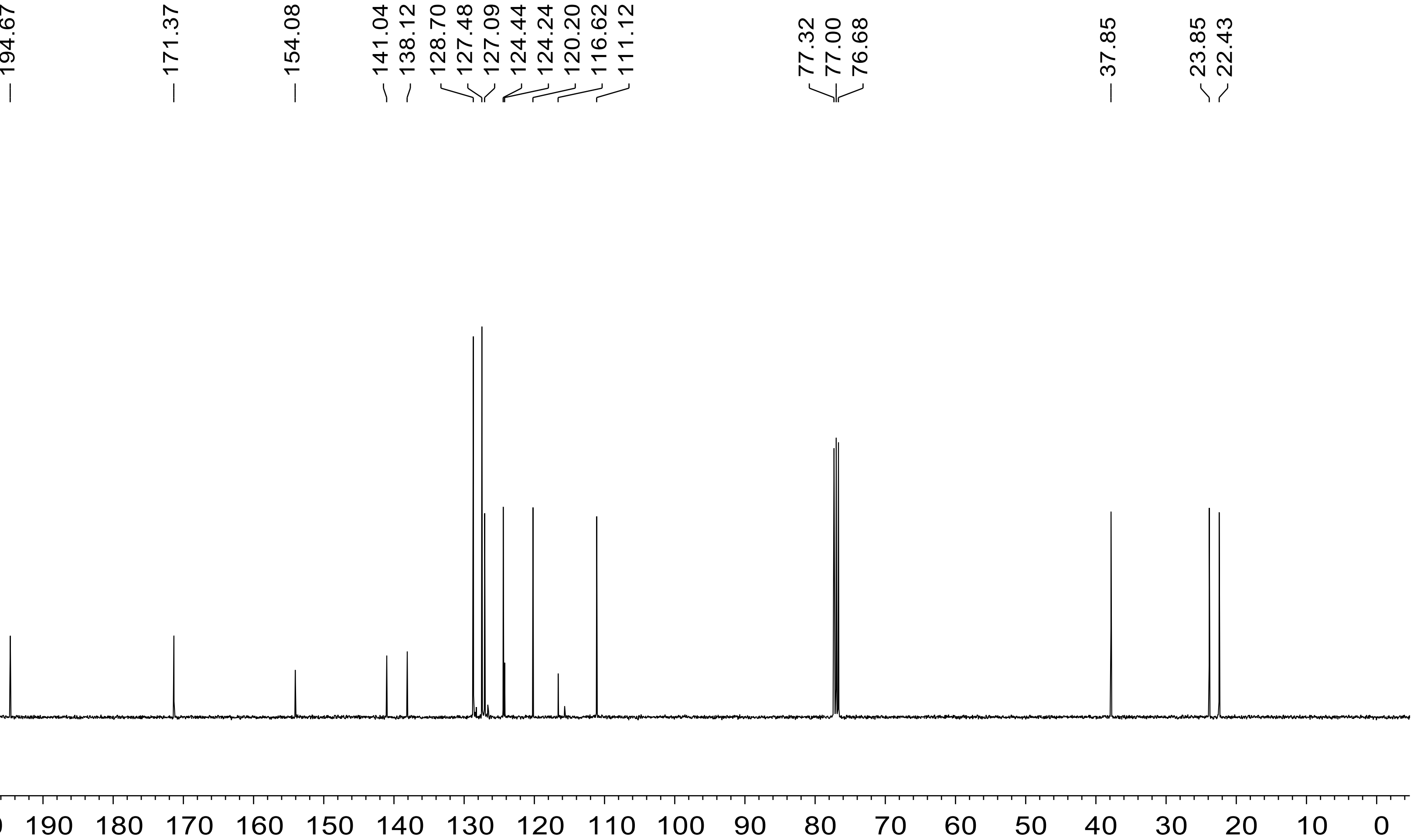

^{1}H NMR (400 MHz, $CDCl_3$) spectrum of compound 3g

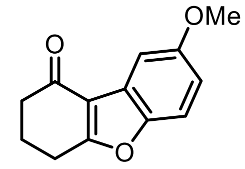

3g

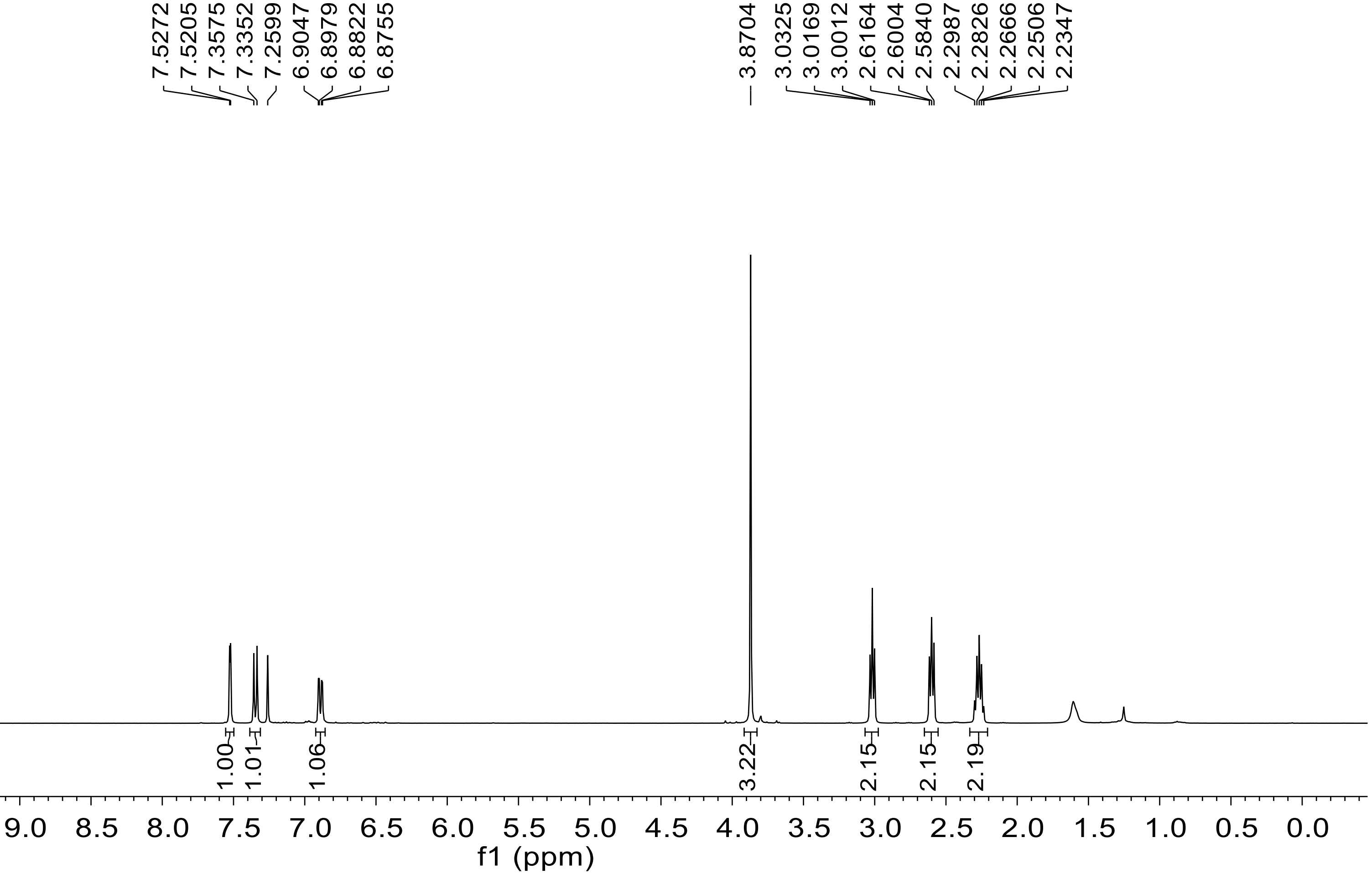

$^{13}C\{^1H\}$ NMR (100 MHz, $CDCl_3$) spectrum of compound 3g

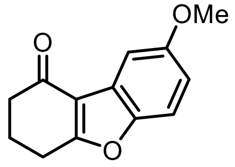

3g

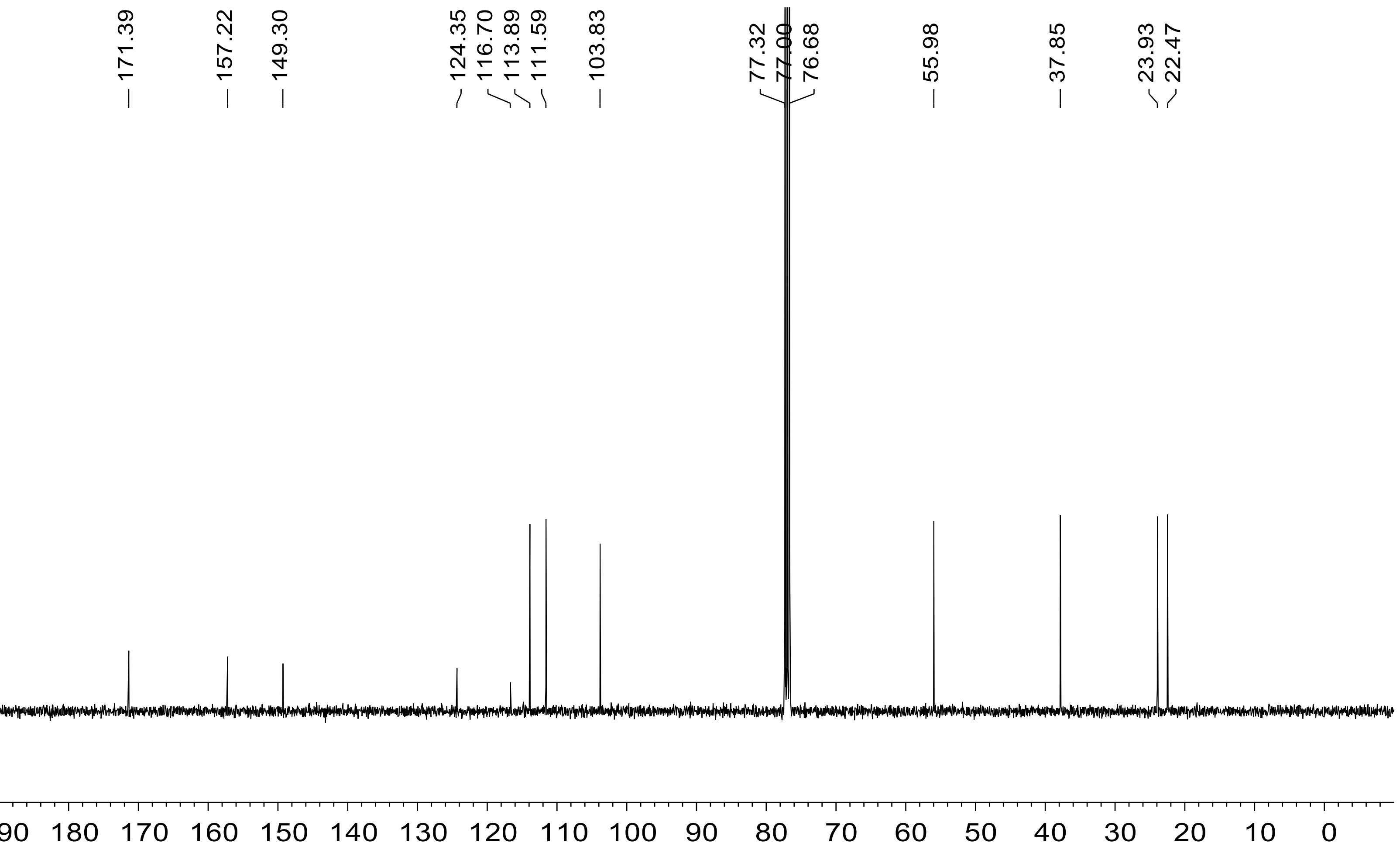

^1H NMR (400 MHz, $CDCl_3$) spectrum of compound 3h

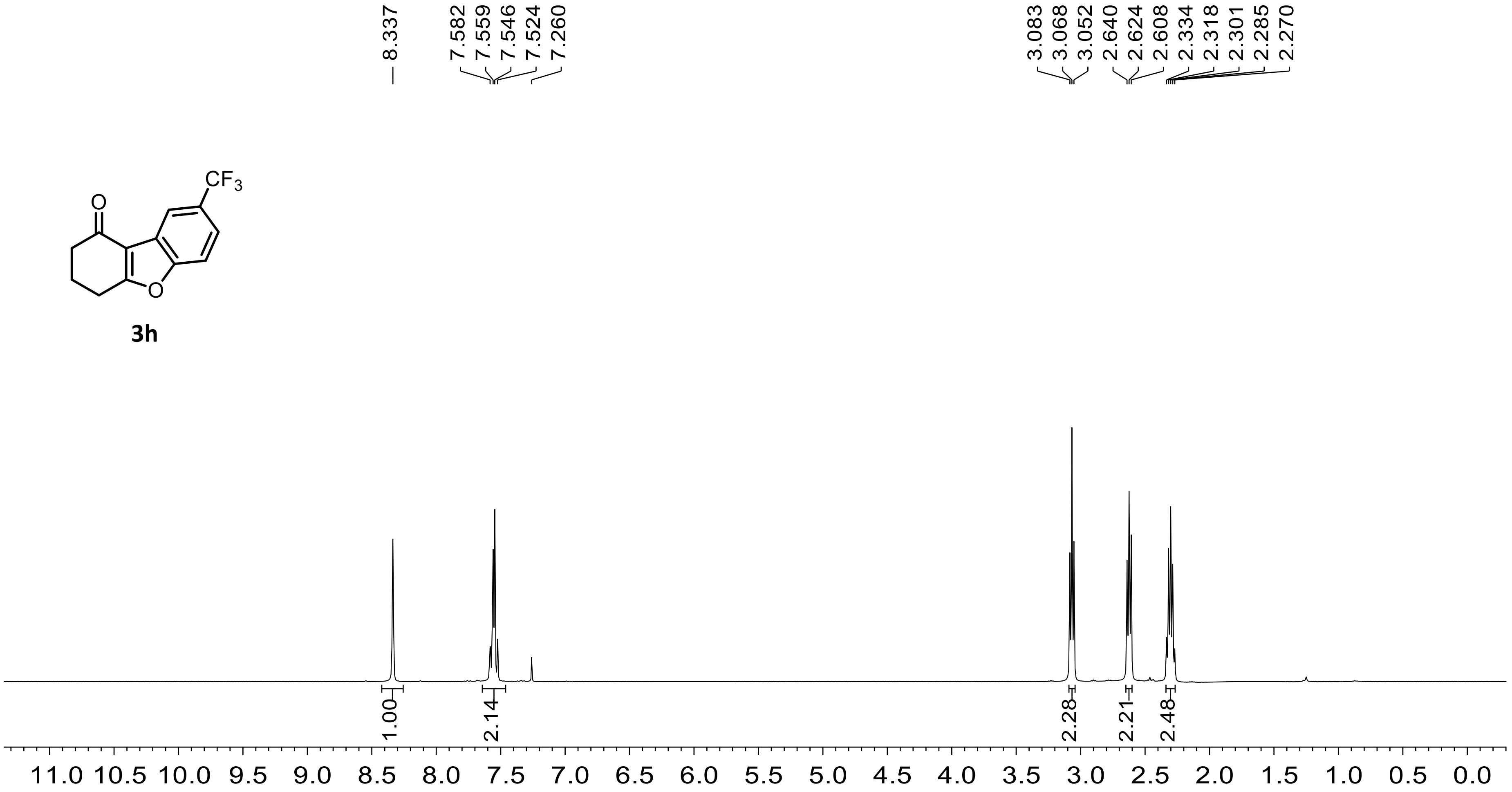

$^{13}C\{^1H\}$ NMR (100 MHz, $CDCl_3$) spectrum of compound 3h

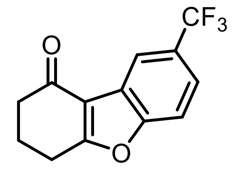

3h

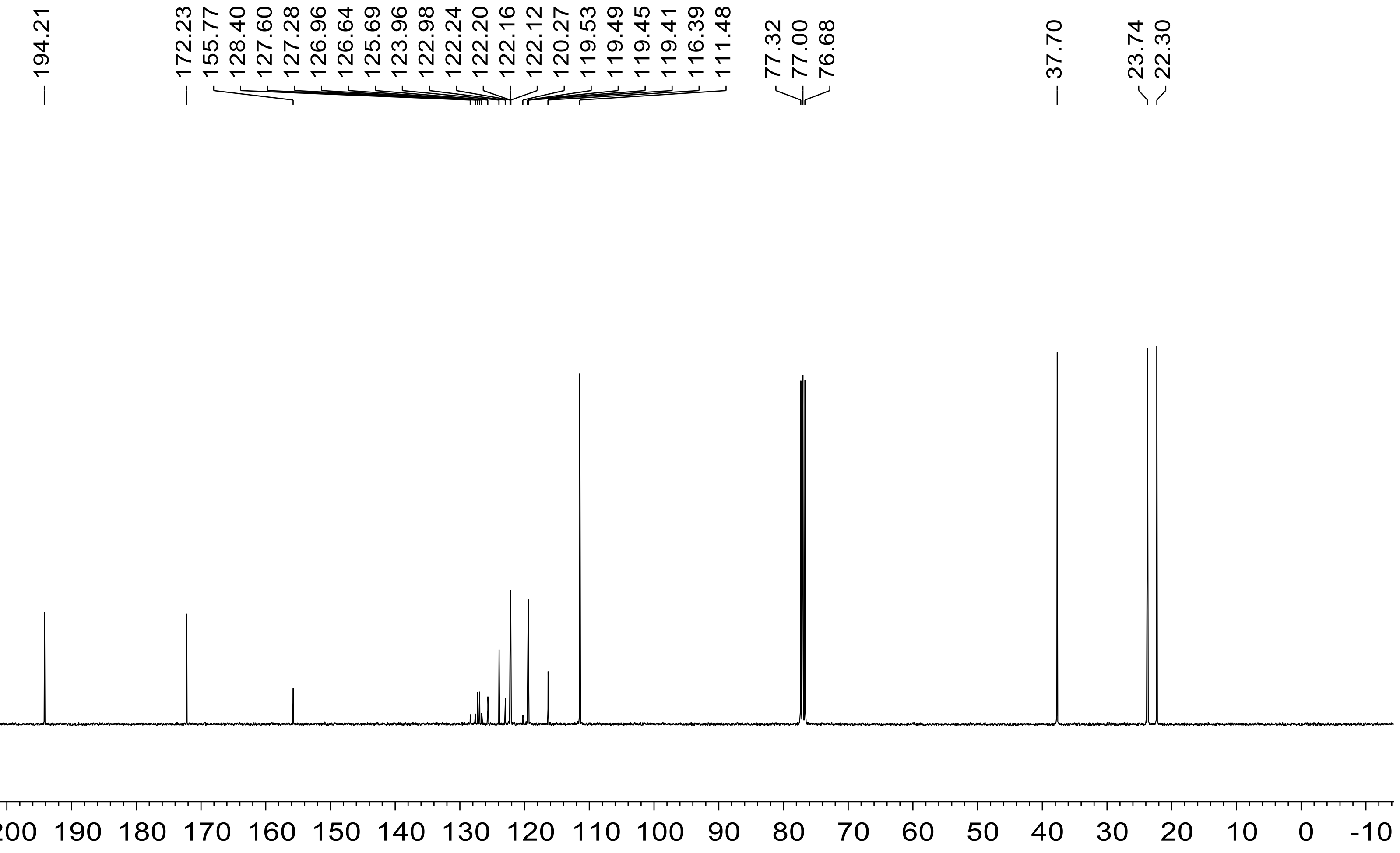

^{1}H NMR (400 MHz, $CDCl_3$) spectrum of compound 3i

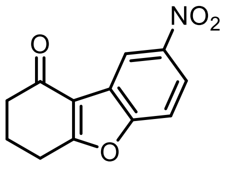

3i

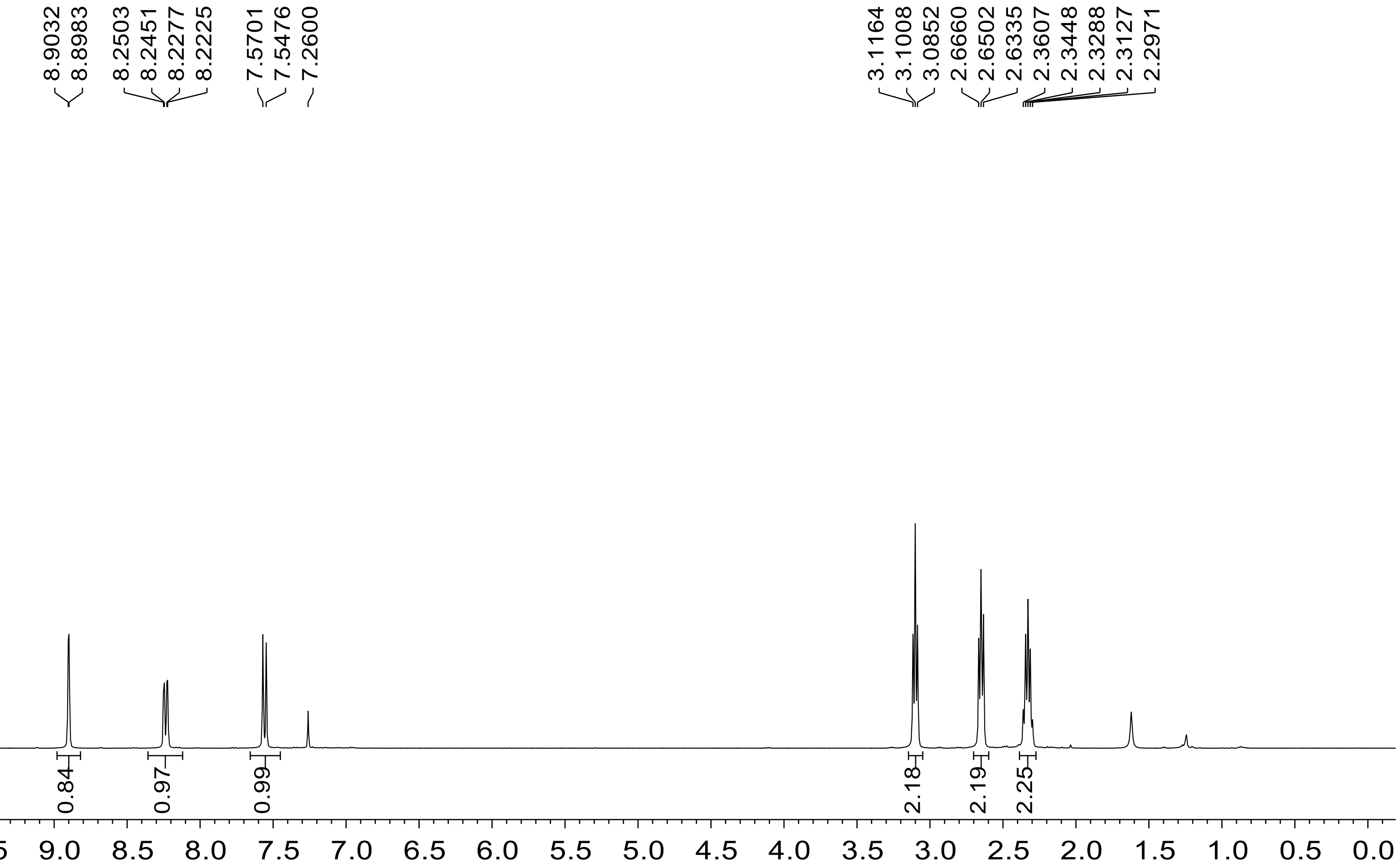

$^{13}C\{^{1}H\}$ NMR (100 MHz, $CDCl_3$) spectrum of compound 3i

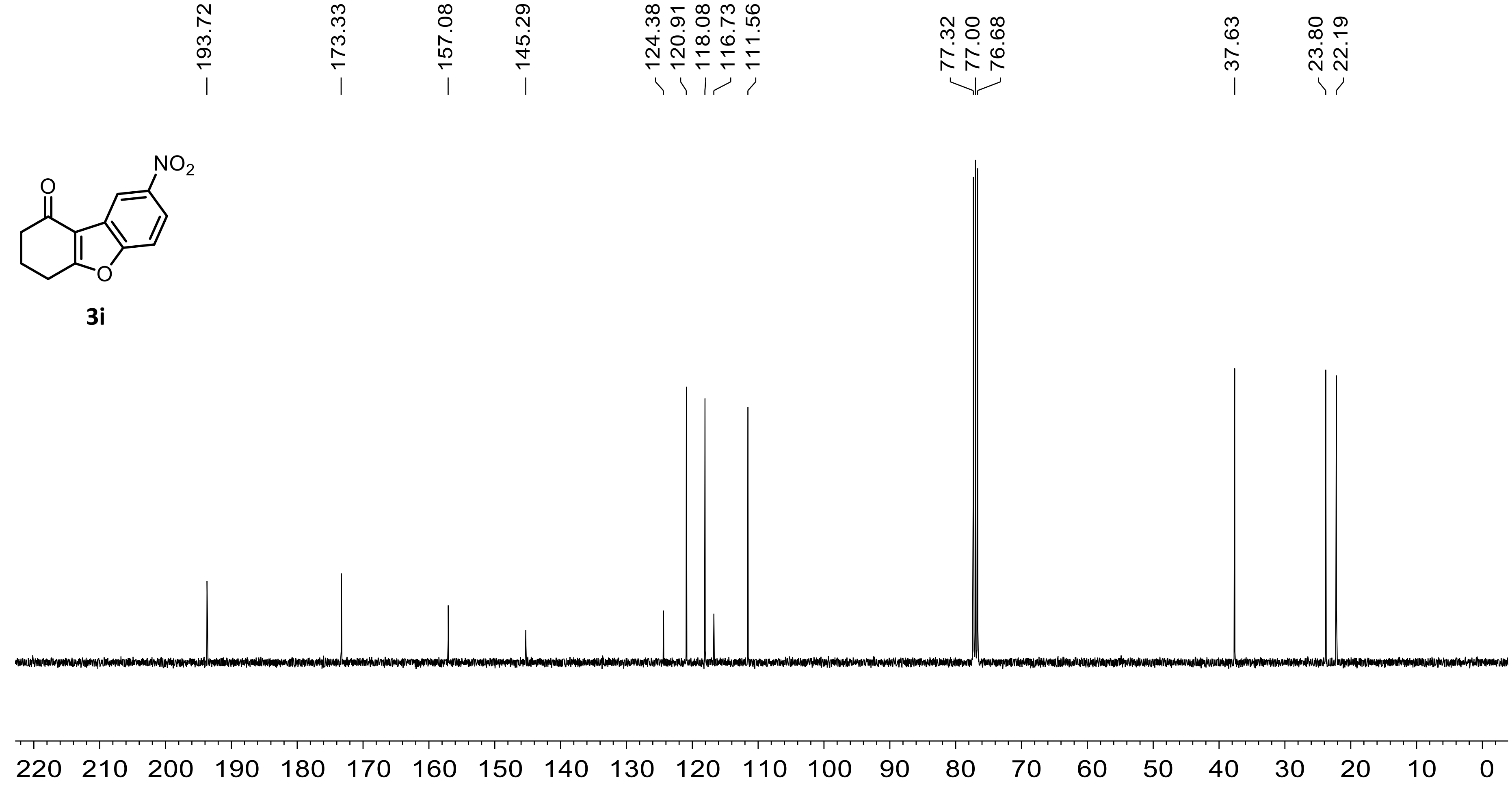

^{1}H NMR (400 MHz, $CDCl_3$) spectrum of compound 3j

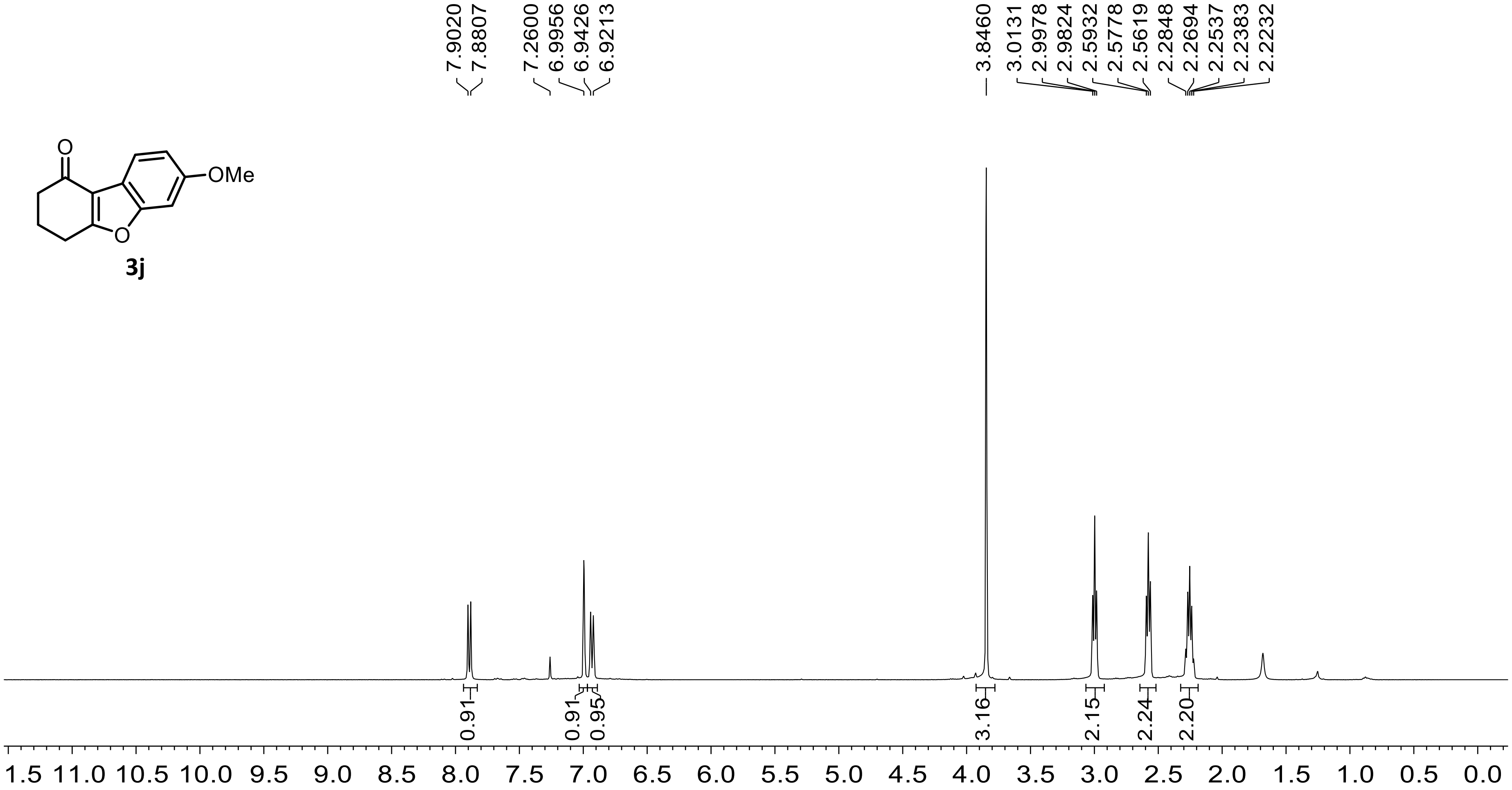

3j

$^{13}C\{^1H\}$ NMR (100 MHz, $CDCl_3$) spectrum of compound 3j

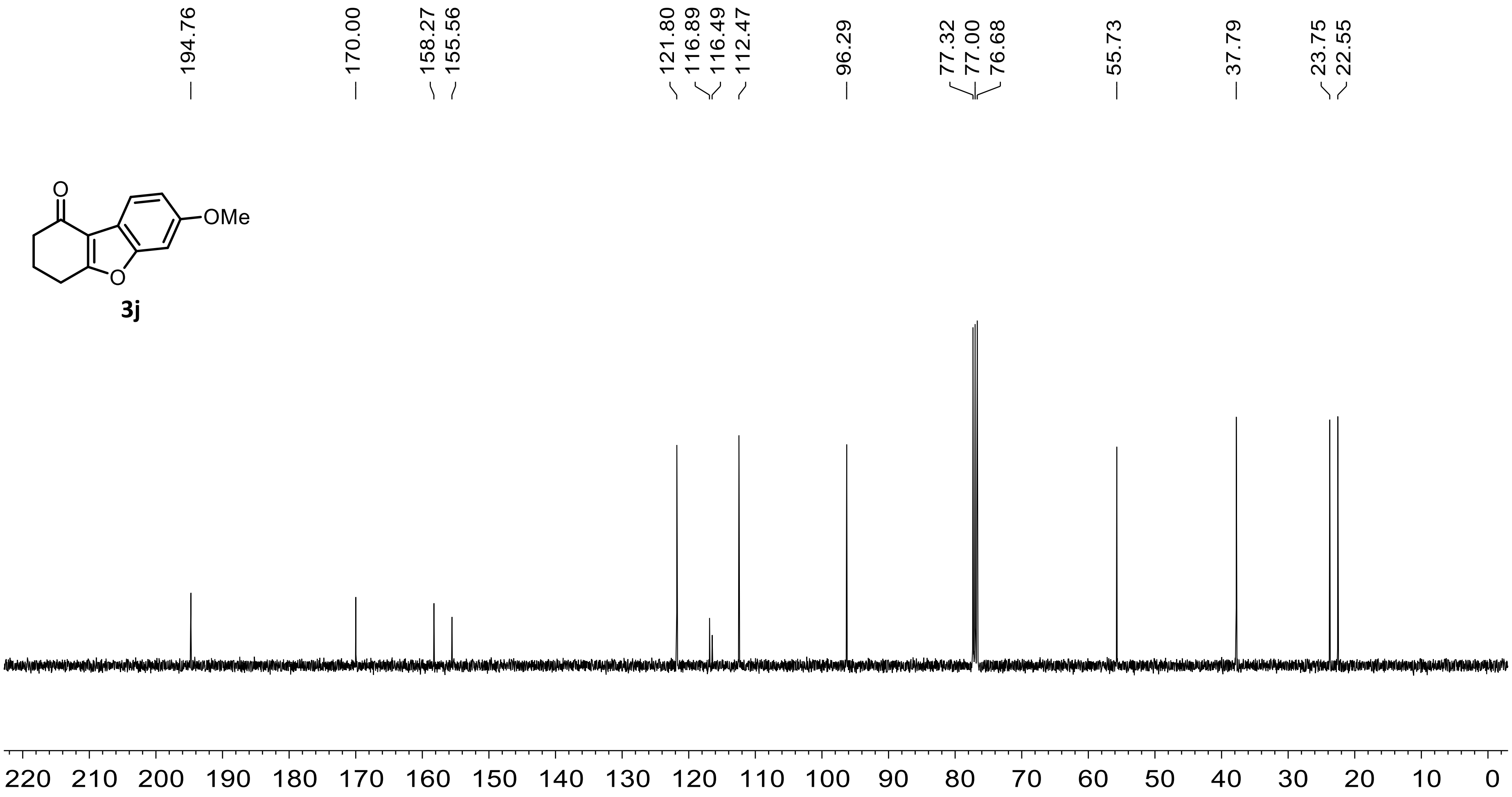

^{1}H NMR (400 MHz, $CDCl_3$) spectrum of compound 3k

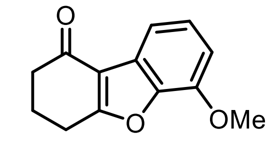

3k

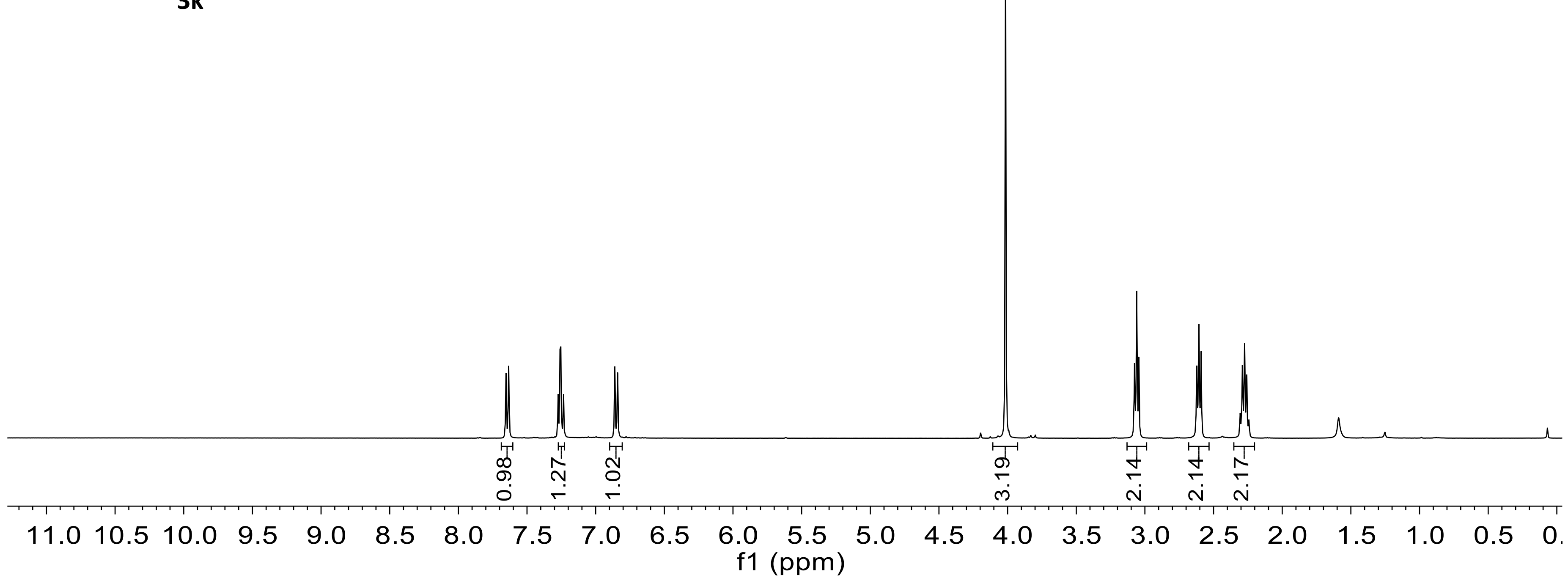

$^{13}C\{^1H\}$ NMR (100 MHz, $CDCl_3$) spectrum of compound 3k

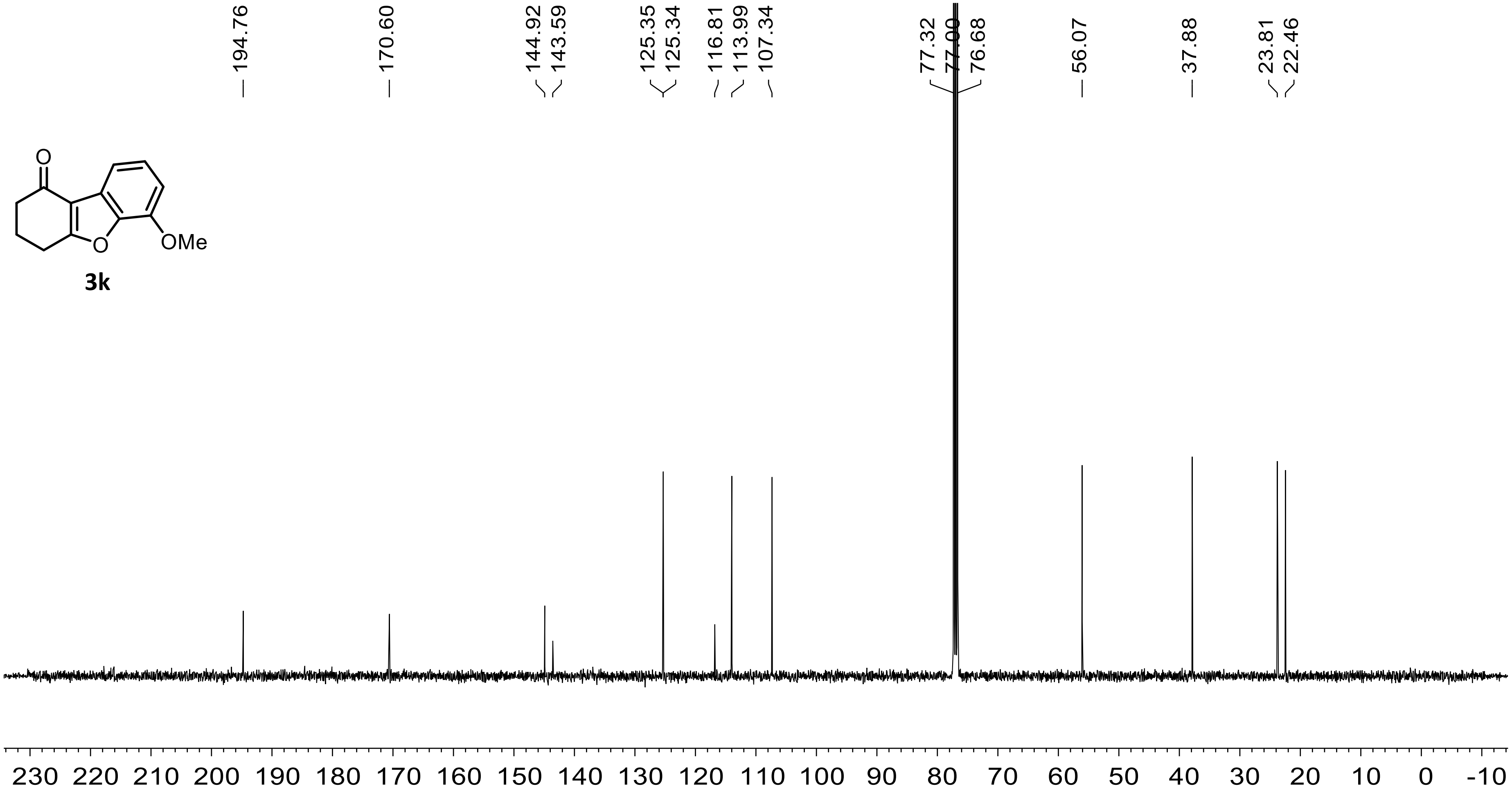

^{1}H NMR (400 MHz, $CDCl_3$) spectrum of compound 3m

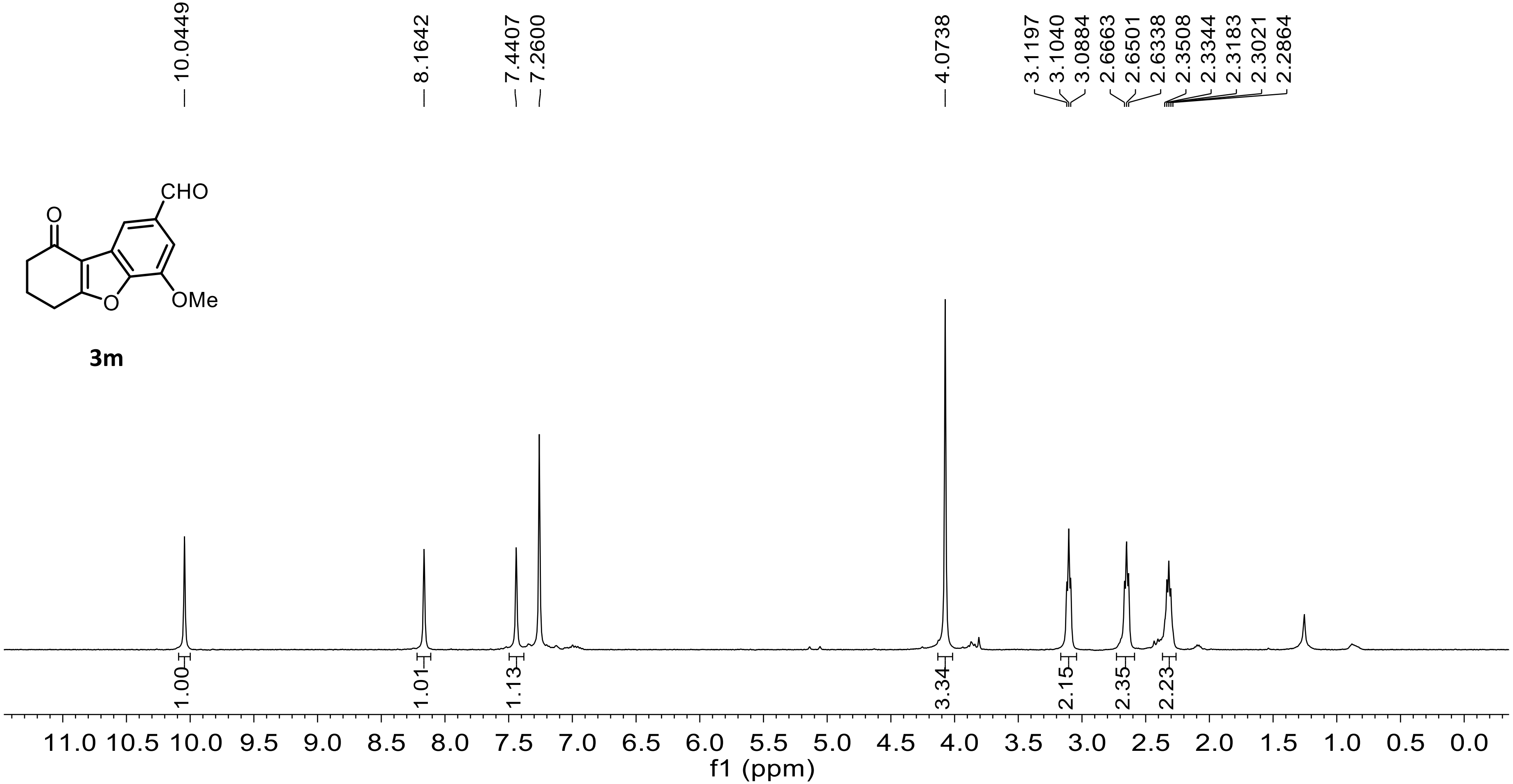

$^{13}C\{^1H\}$ NMR (100 MHz, $CDCl_3$) spectrum of compound 3m

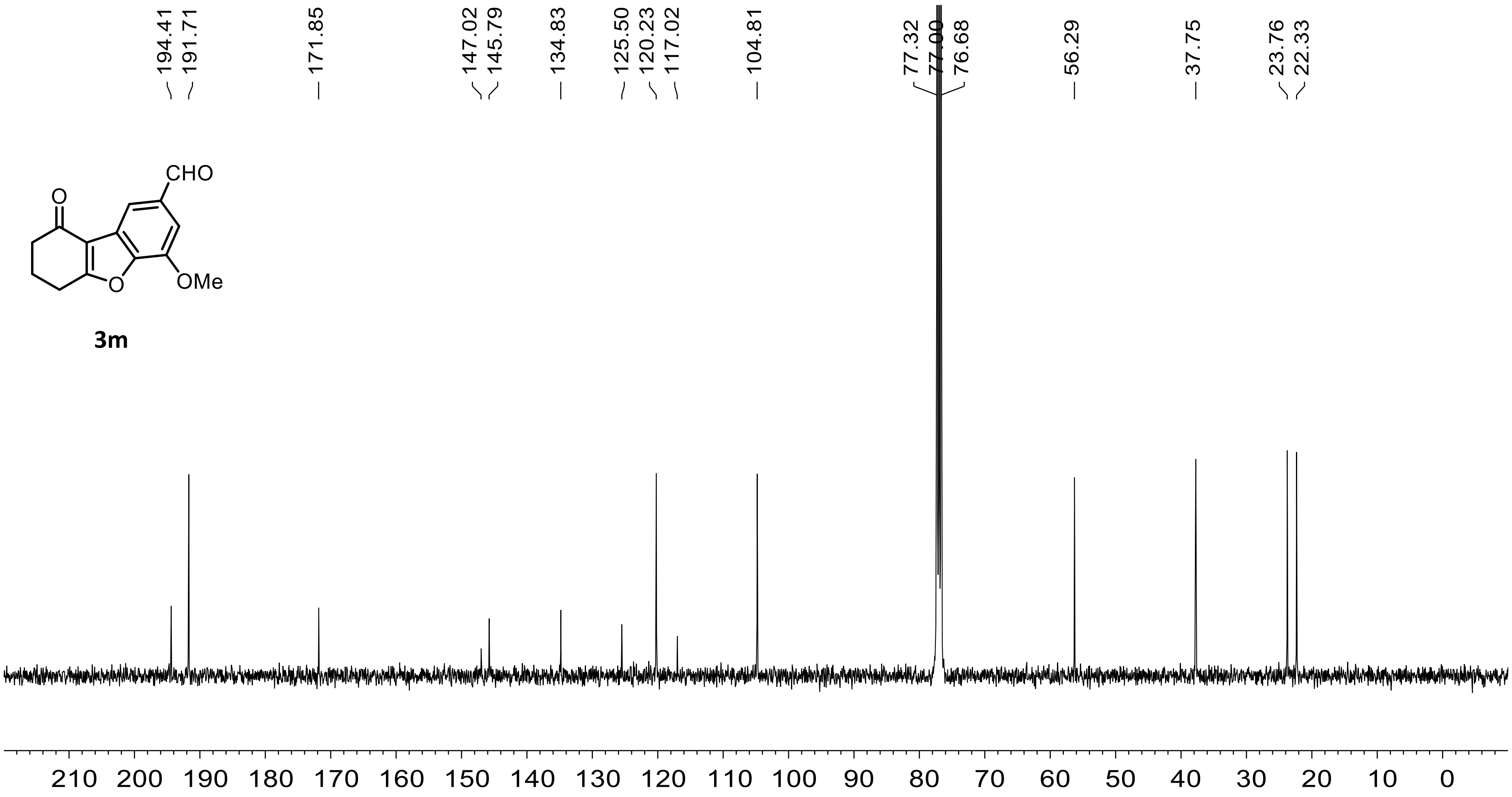

^{1}H NMR (400 MHz, $CDCl_3$) spectrum of compound 3n

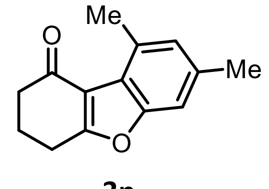

3n

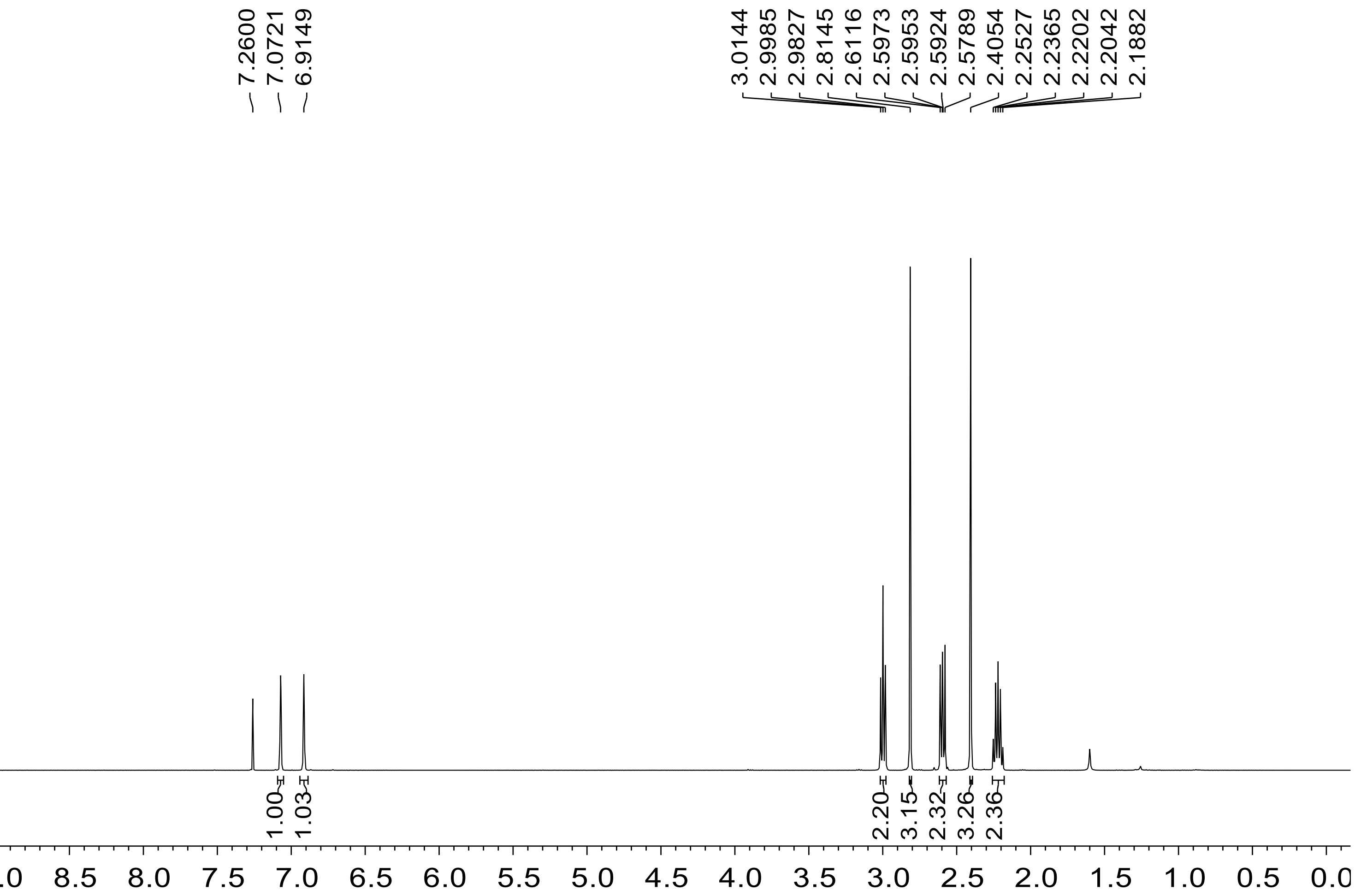

$^{13}C\{^1H\}$ NMR (100 MHz, $CDCl_3$) spectrum of compound 3n

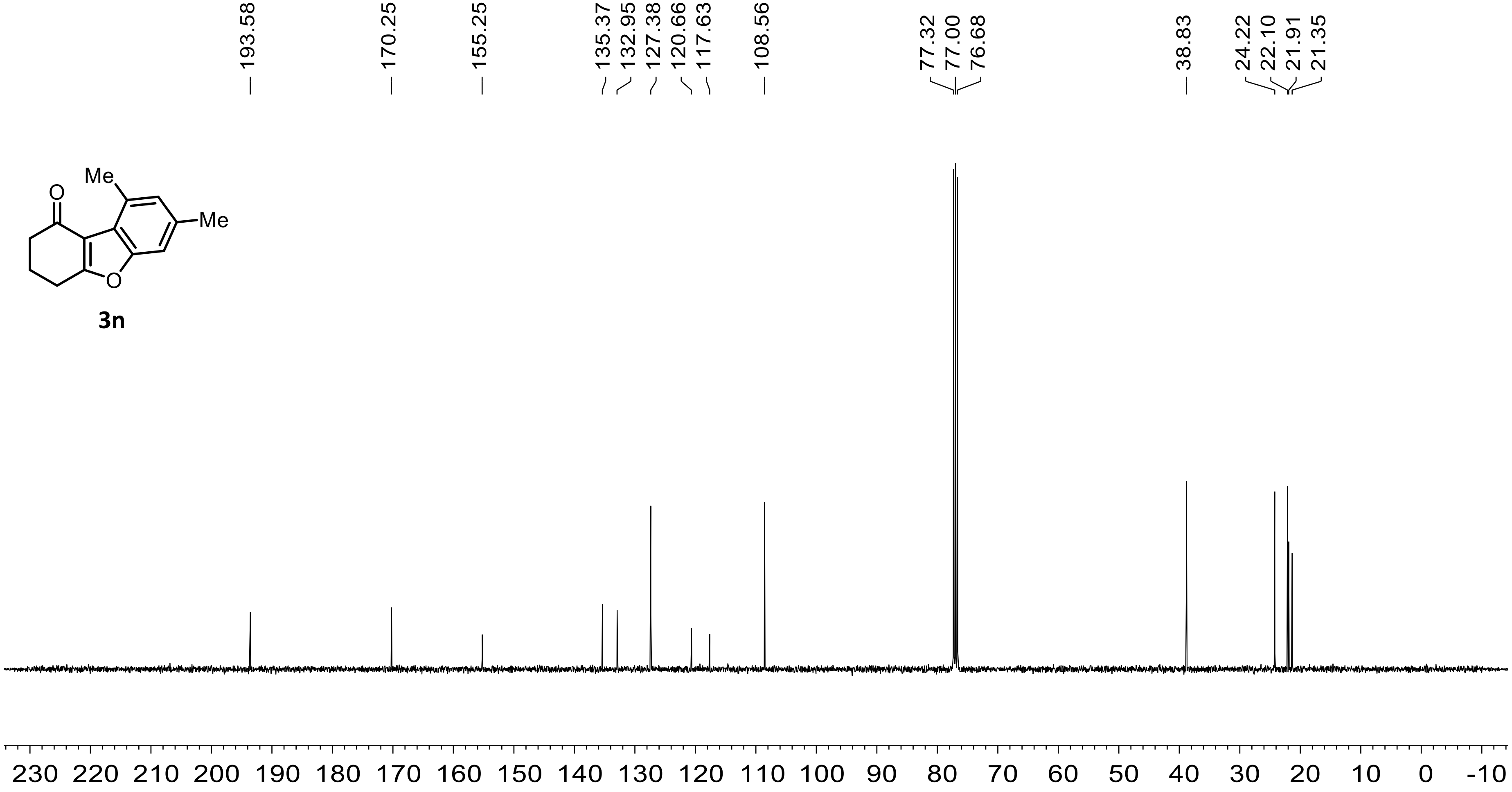

^{1}H NMR (400 MHz, $CDCl_3$) spectrum of compound 3o

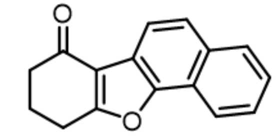

3o

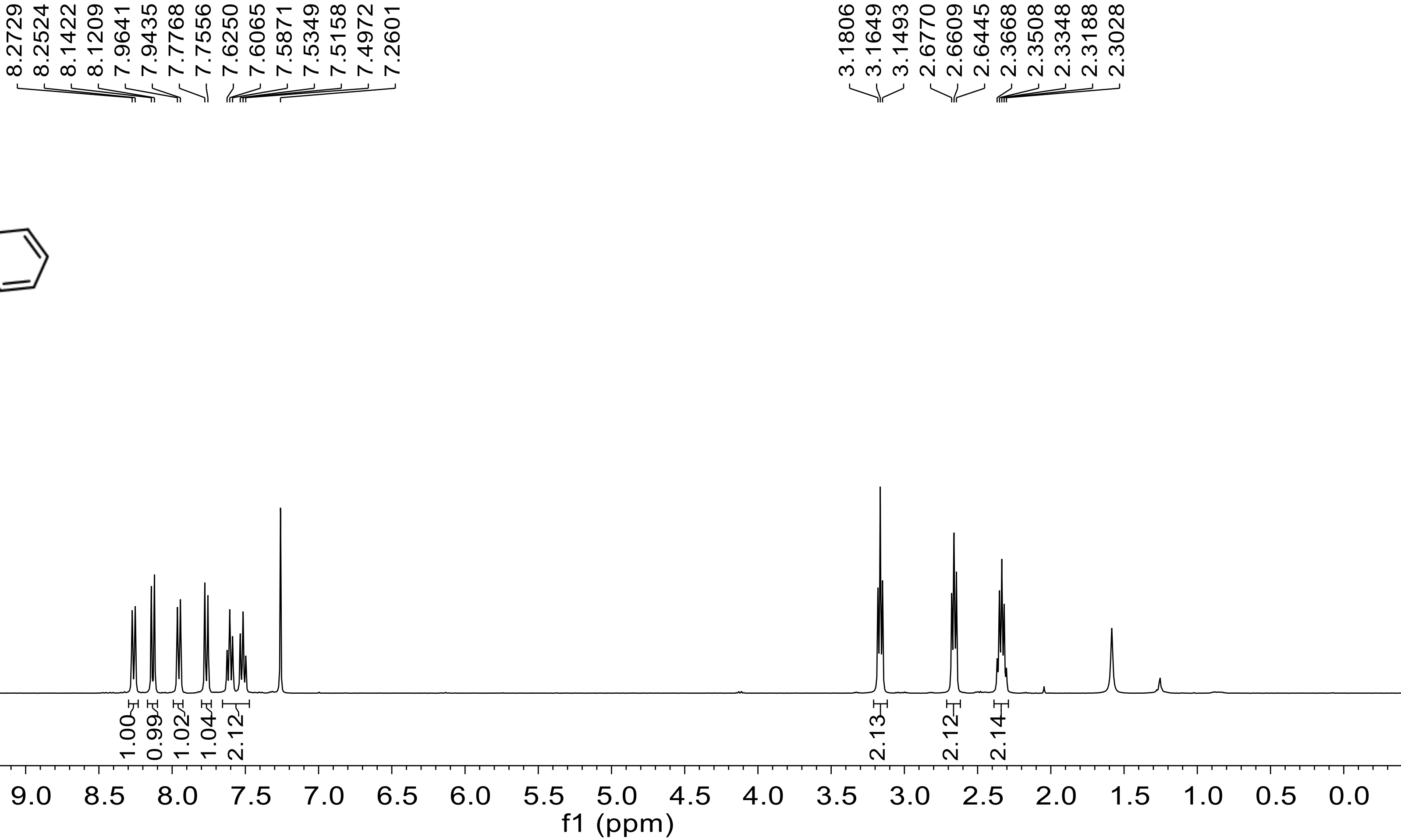

$^{13}C\{^1H\}$ NMR (100 MHz, $CDCl_3$) spectrum of compound 3o

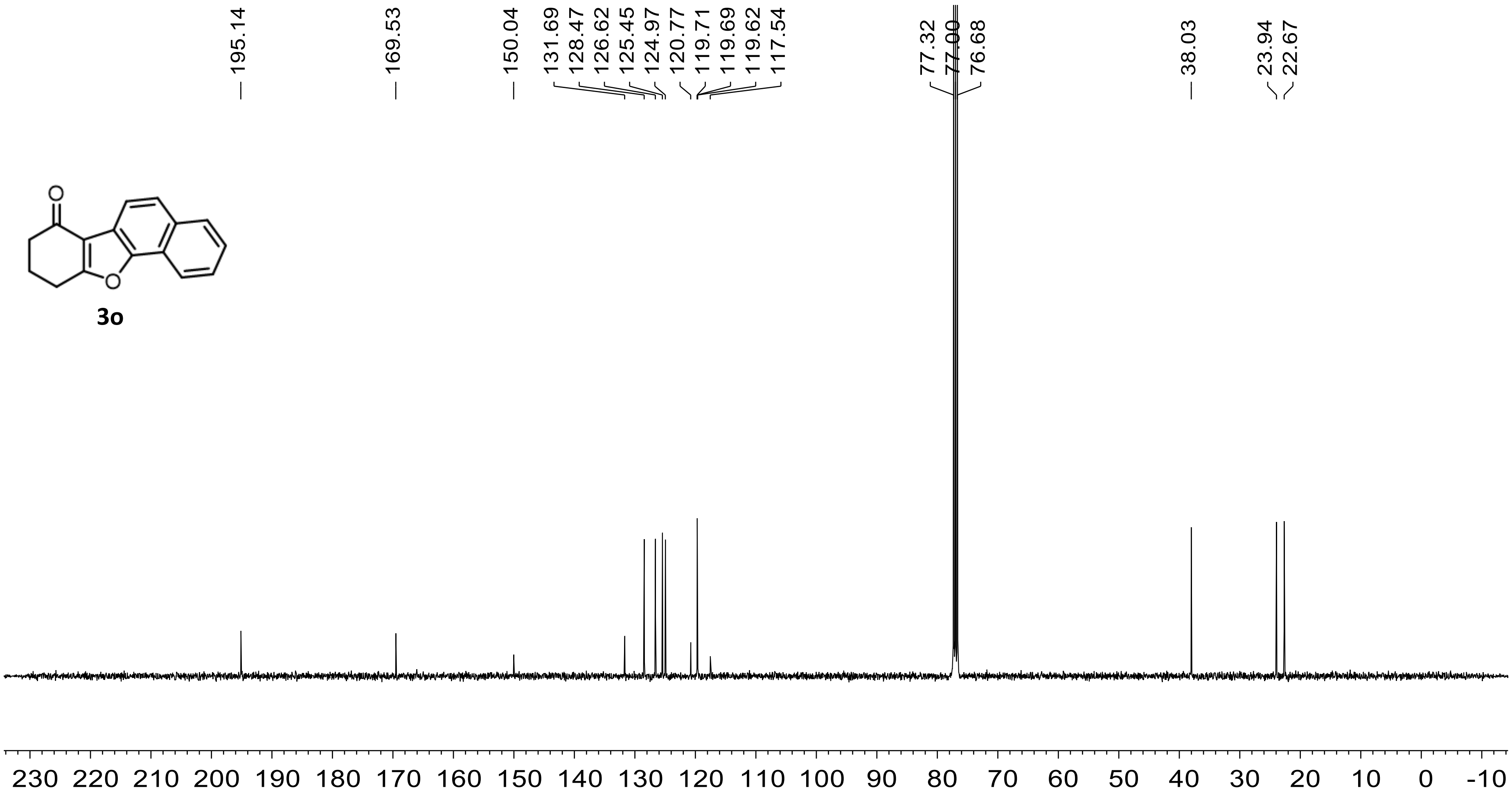

3o

^{1}H NMR (400 MHz, $CDCl_3$) spectrum of compound 3p

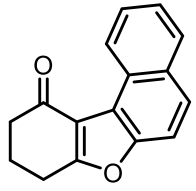

3p

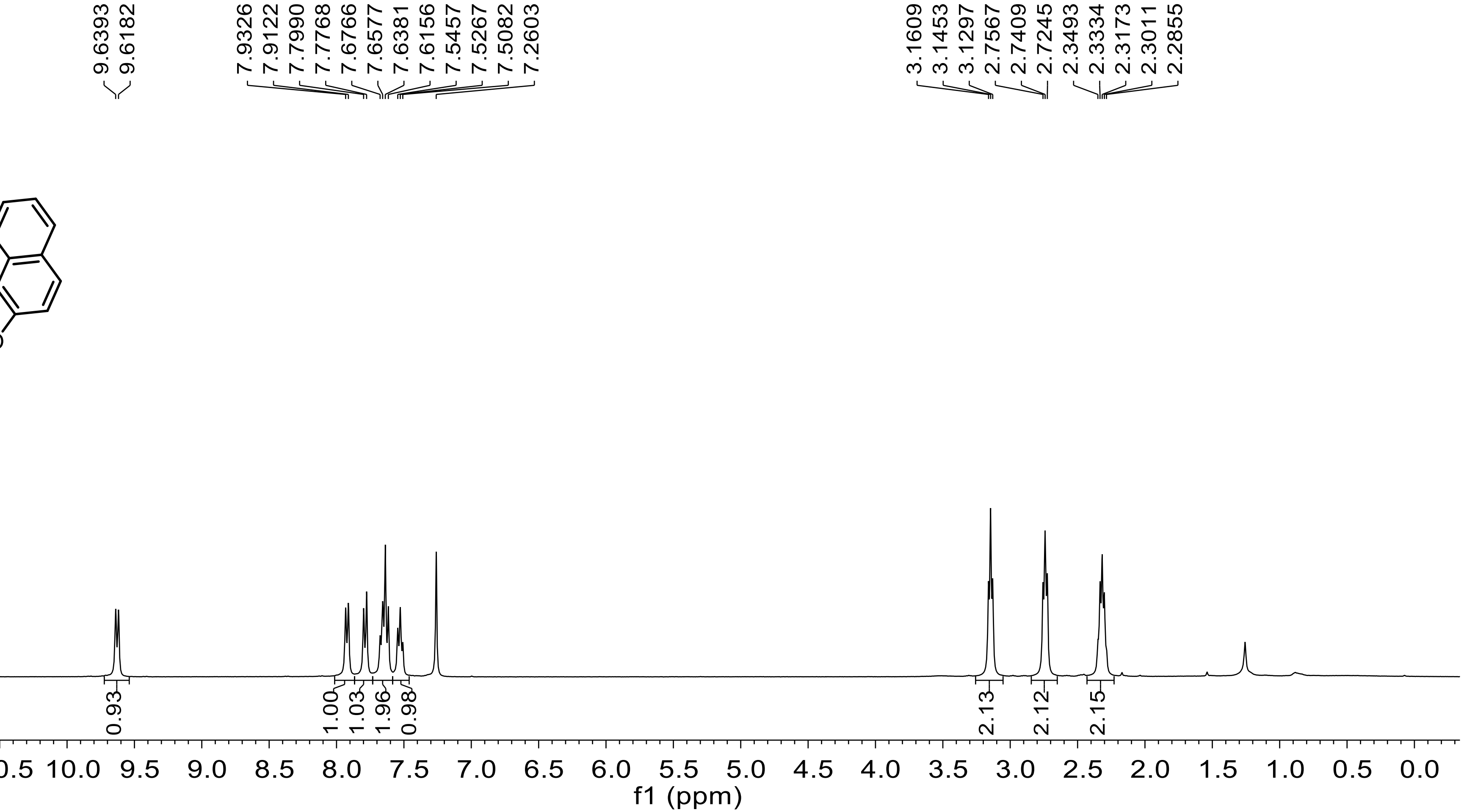

$^{13}C\{^{1}H\}$ NMR (100 MHz, $CDCl_3$) spectrum of compound 3p

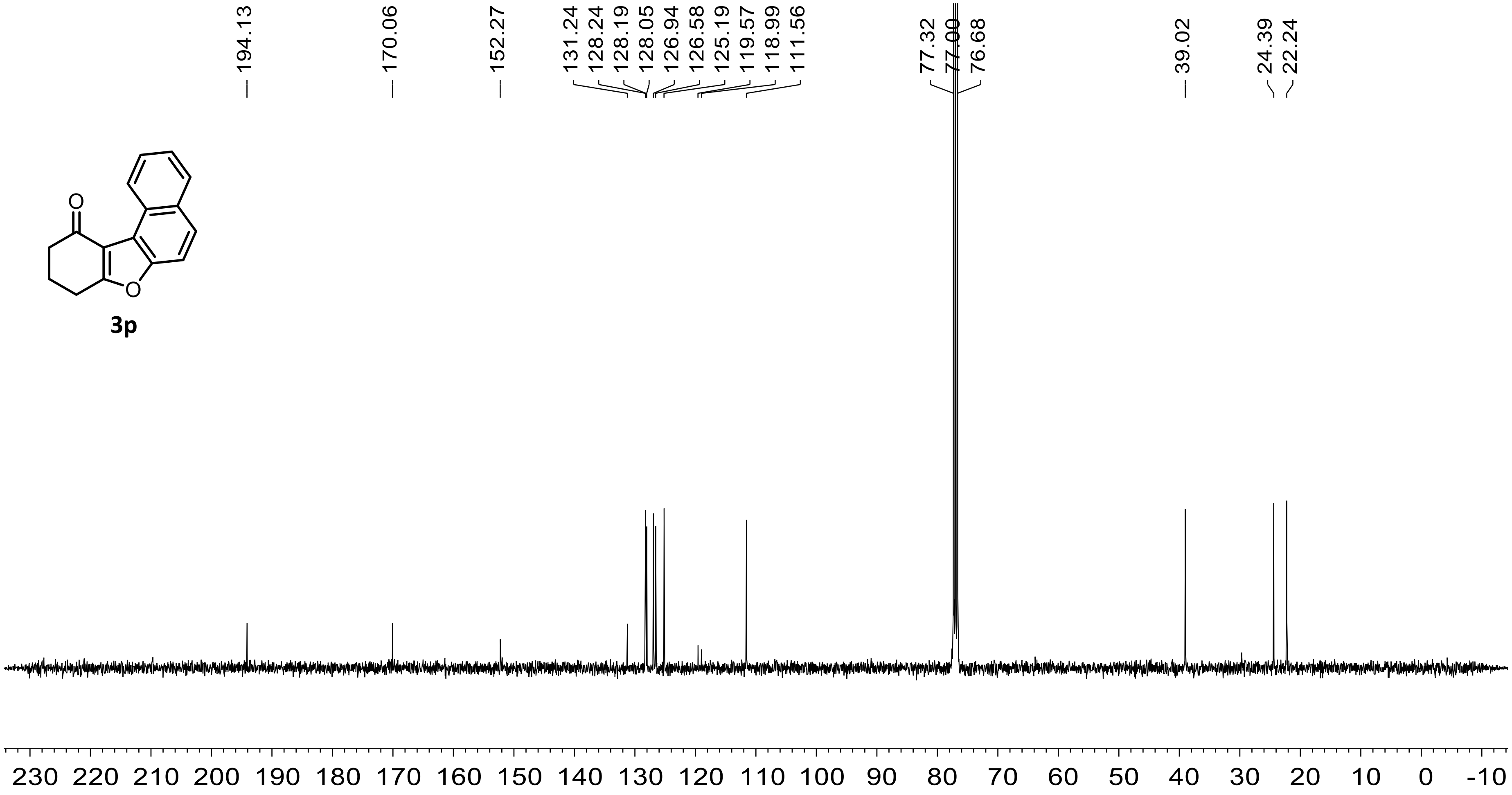

^{1}H NMR (400 MHz, $CDCl_3$) spectrum of compound 3q

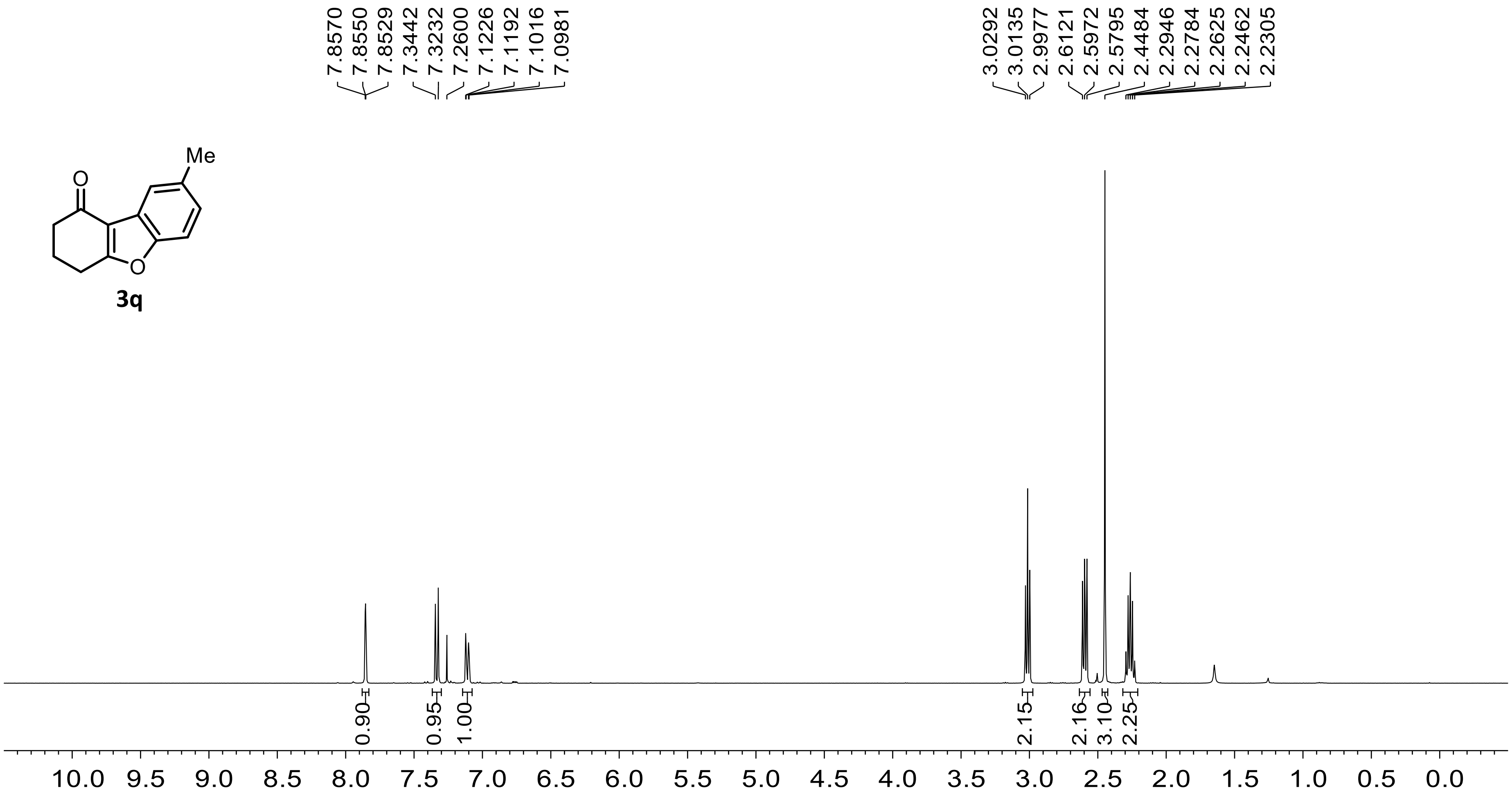

$^{13}C\{^1H\}$ NMR (100 MHz, $CDCl_3$) spectrum of compound 3q

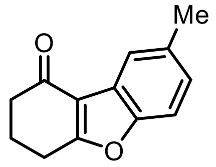

3q

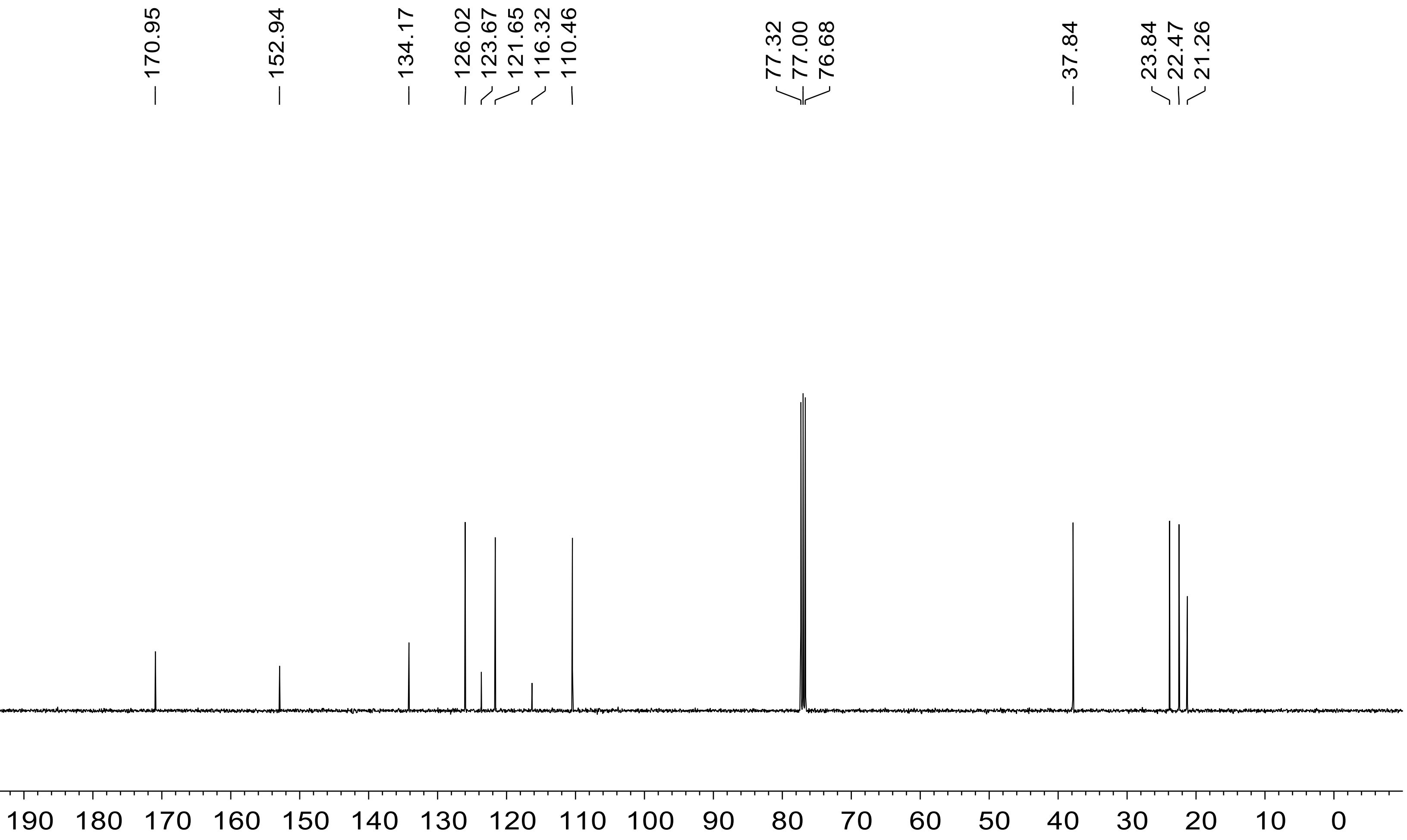

^{1}H NMR (400 MHz, $CDCl_3$) spectrum of compound 3r

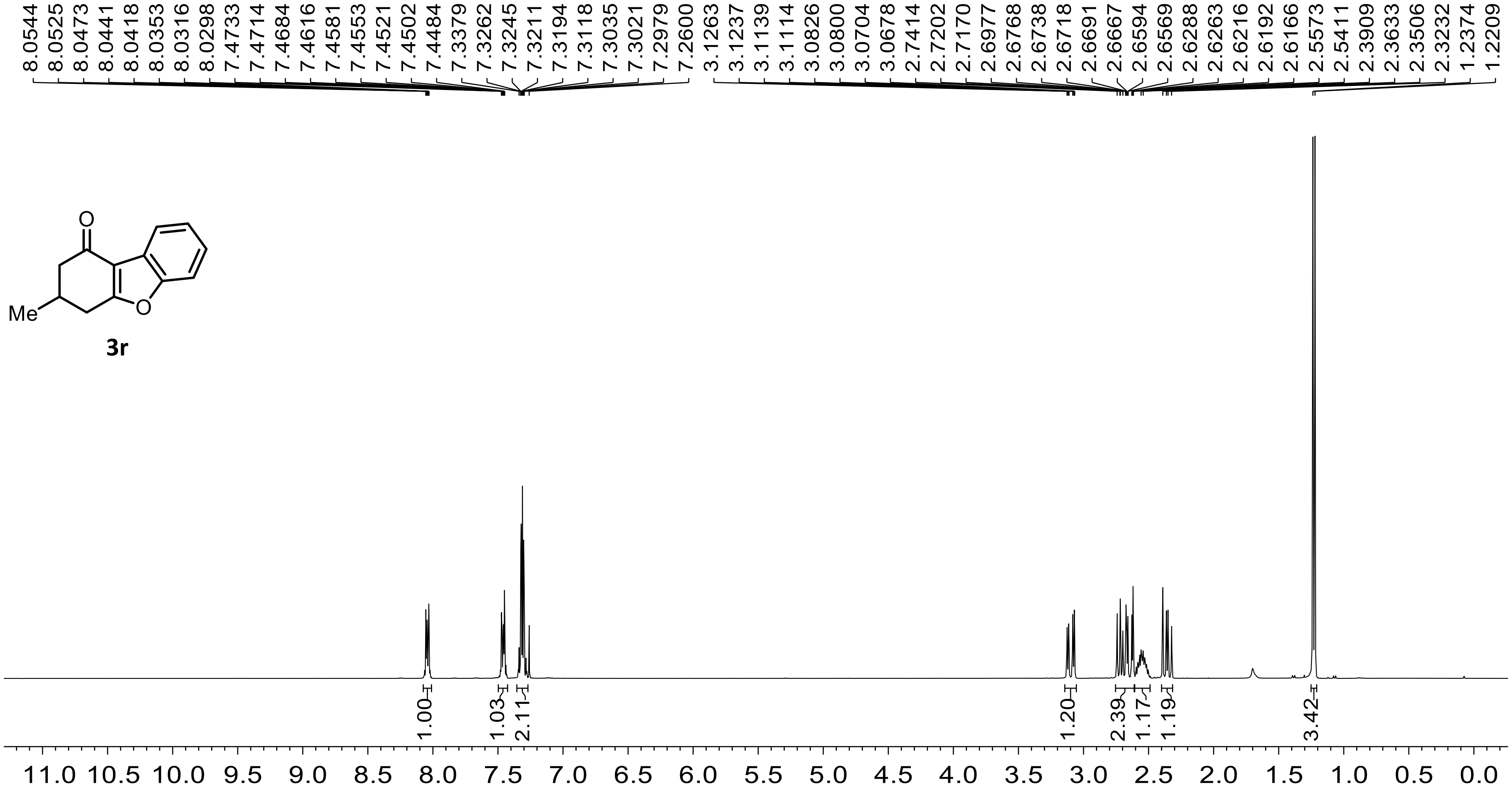

$^{13}C\{^1H\}$ NMR (100 MHz, $CDCl_3$) spectrum of compound 3r

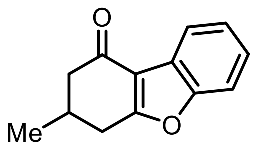

3r

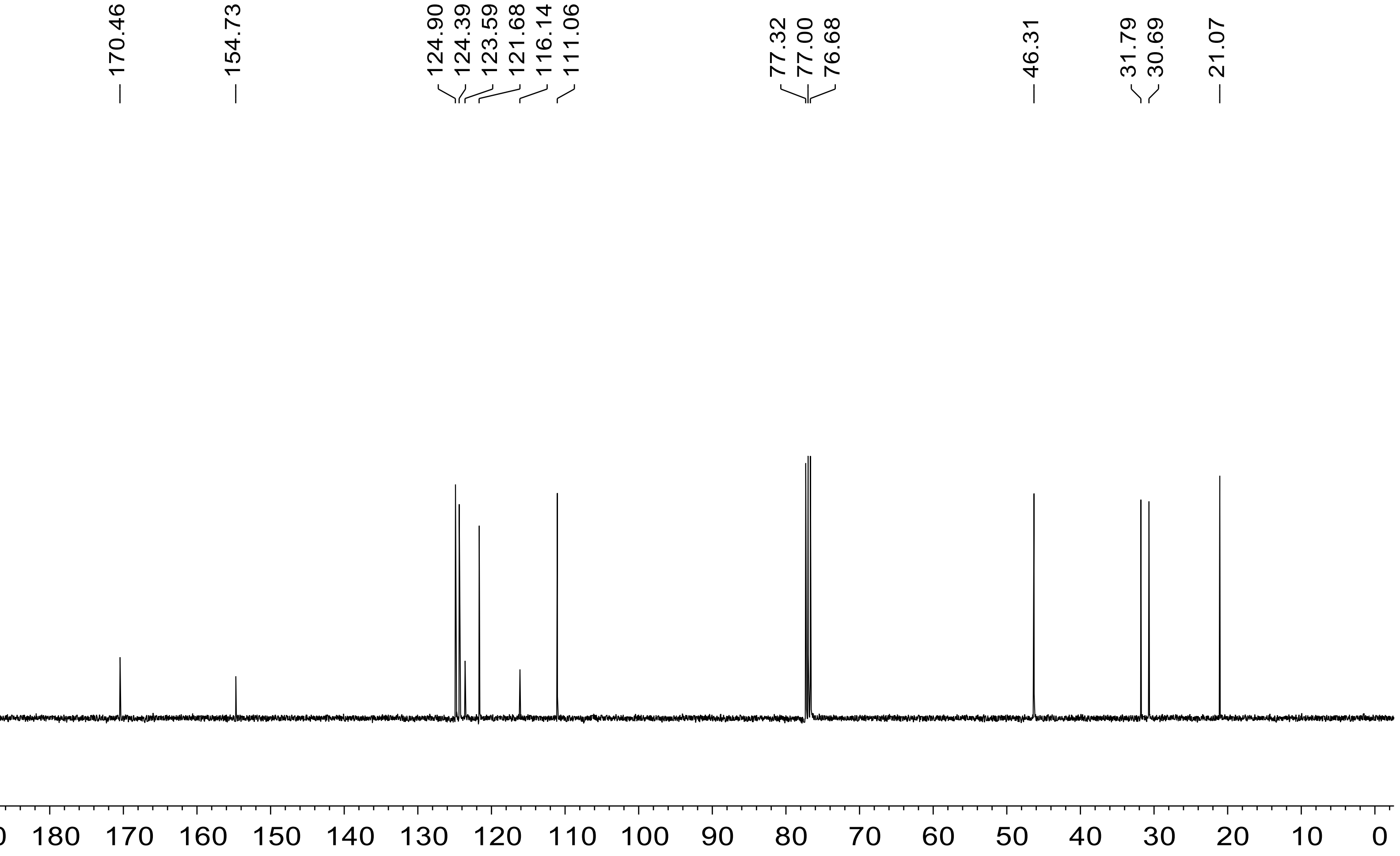

^{1}H NMR (400 MHz, $CDCl_3$) spectrum of compound 3s

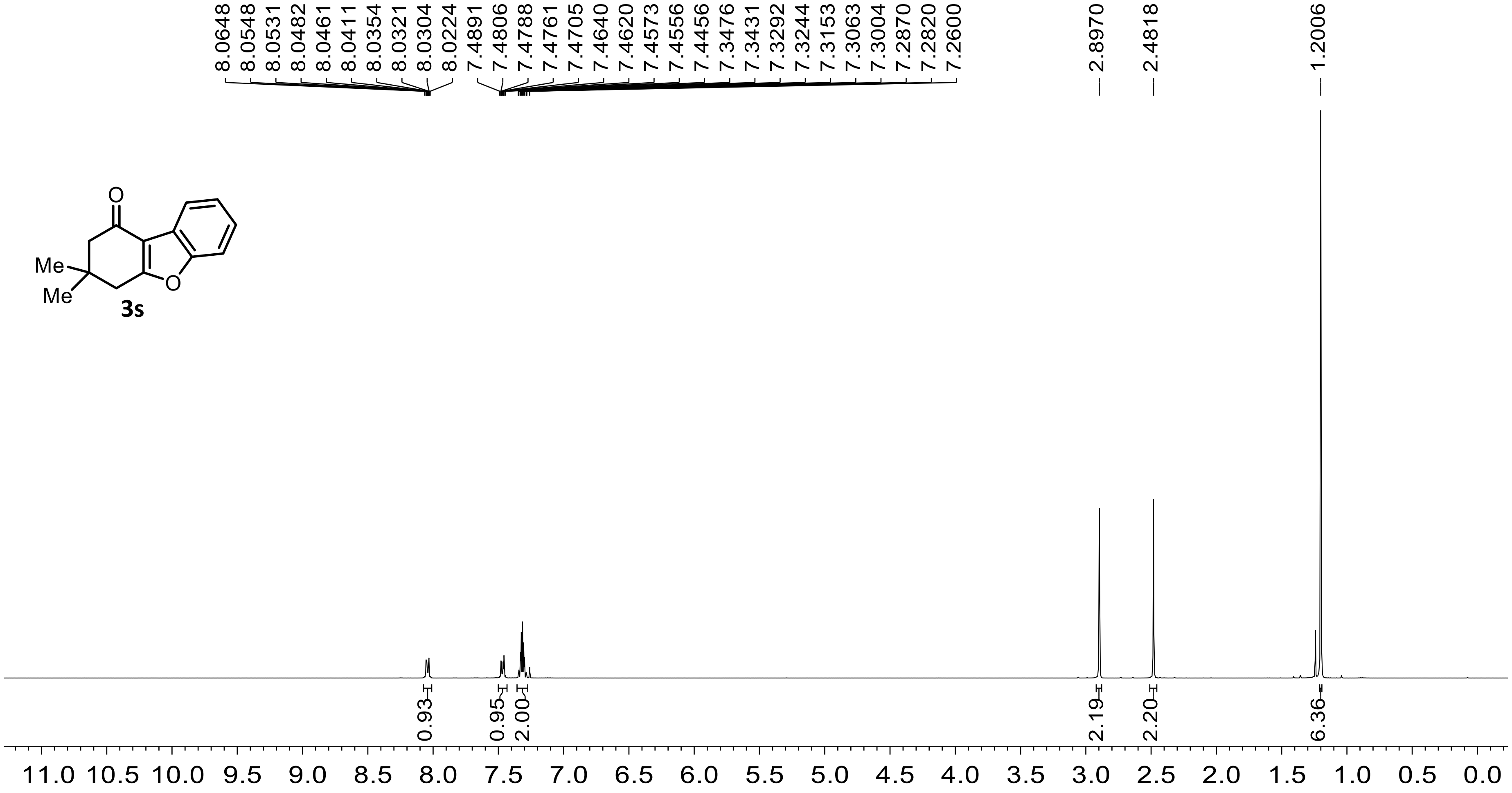

Supplement: Supplementary file 1 — jo4c02167_si_001.pdf [file jo4c02167_si_001.pdf]
